# ﻿Old World *Micropholcus* spiders, with first records of acrocerid parasitoids in Pholcidae (Araneae)

**DOI:** 10.3897/zookeys.1213.133178

**Published:** 2024-09-26

**Authors:** Bernhard A. Huber, Guanliang Meng

**Affiliations:** 1 Zoological Research Museum Alexander Koenig, LIB, Bonn, Germany Zoological Research Museum Alexander Koenig, LIB Bonn Germany

**Keywords:** CO1 barcode, genetic distances, Morocco, Oman, Philippines, Saudi Arabia, species delimitation, taxonomy

## Abstract

*Micropholcus* Deeleman-Reinhold & Prinsen, 1987 is one of only two Pholcidae genera known to occur both in the Old and New Worlds. However, there are major morphological and ecological differences among geographically separate groups of species, and it was mainly molecular data that have resulted in our current view of uniting all these species into a single genus. In the Old World, only four species have previously been described. Here, current knowledge about Old World *Micropholcus* is reviewed, redescribing three of the four previously known species, and describing twelve new species, originating from Saudi Arabia (*M.dhahran* Huber, **sp. nov.**, *M.harajah* Huber, **sp. nov.**, *M.alfara* Huber, **sp. nov.**, *M.abha* Huber, **sp. nov.**, *M.tanomah* Huber, **sp. nov.**, *M.bashayer* Huber, **sp. nov.**, *M.maysaan* Huber, **sp. nov.**), Oman (*M.darbat* Huber, **sp. nov.**, *M.shaat* Huber, **sp. nov.**), Morocco (*M.ghar* Huber, **sp. nov.**, *M.khenifra* Huber, Lecigne & Lips, **sp. nov.**), and the Philippines (*M.bukidnon* Huber, **sp. nov.**). We provide an exploratory species delimitation analysis based on CO1 barcodes, extensive SEM data, and first records of Acroceridae (Diptera) larvae in Pholcidae, extracted from book lungs.

## ﻿﻿Introduction

Pholcid spiders have a worldwide distribution, with the large majority of species restricted to tropical and subtropical regions (Huber and Chao 2019). A few species have followed humans around the world, but in general, distributions of most species and even genera are limited to much smaller geographic regions. Only two of the currently recognised 95 extant genera are known to be present in both the Old and New Worlds: *Pholcus* Walckenaer, 1805 and *Micropholcus* Deeleman-Reinhold & Prinsen, 1987. *Pholcus* is very species-rich and widely distributed in the Old World, but in the New World limited to a few species geographically restricted to the USA. These New World *Pholcus* species are considered to constitute a monophyletic group, and nine of the ten formally described species are known from the TGA area only (Tennessee, Georgia, Alabama), suggesting a relict distribution (Huber 2011).

*Micropholcus* was originally thought be an Old World genus, represented by only two species: the pantropical type species *M.fauroti* (Simon, 1887) and the putatively closely related *M.jacominae* Deeleman-Reinhold & van Harten, 2001 from the Arabian Peninsula (Yemen). Molecular data then suggested that numerous New World species running under the name *Leptopholcus* Simon, 1893 were in fact misplaced and more closely related to *M.fauroti* than to the type species of *Leptopholcus* (Dimitrov et al. 2013). These misplaced species were formally transferred to *Micropholcus* in Huber et al. (2014), making *Micropholcus* appear much more diverse in the New than in the Old World. Geographically, New World *Micropholcus* appear largely restricted to the Greater Antilles and to semi-arid regions in Brazil (Huber et al. 2005; Huber and Wunderlich 2006; Huber et al. 2014).

In 2018, it was again molecular data that showed that Old World *Micropholcus* have a much wider distribution than suggested by the single known autochthonous species from the Arabian Peninsula. Huber et al. (2018) (based on the data in Eberle et al. 2018) transferred the Moroccan *Pholcusagadir* Huber, 2011 to *Micropholcus*, and an undescribed species from the Philippines was also placed in *Micropholcus*, as “*M.* Phi114”. Here we review all the available data about Old World *Micropholcus*, describing the species from the Philippines and several additional new species from the Arabian Peninsula and Morocco, and redescribing previously published species as far as possible.

## ﻿﻿Materials and methods

### ﻿Material examined

This study is based on the examination of 362 adult specimens of Old World *Micropholcus*, deposited in the following institutions: Muséum d’histoire naturelle, Genève (**MHNG**); Museum of Arthropods, College of Food and Agriculture Sciences, King Saud University, Riyadh (**KSMA**); Naturalis Biodiversity Center, Leiden (**RMNH**), and Zoologisches Forschungsmuseum Alexander Koenig, Bonn (**ZFMK**). Further material deposited in Institut Royal des Sciences Naturelles de Belgique (Brussels), Collection Carles Ribera (Barcelona), and Collection Robert Bosmans (Ghent), was examined by the first author in 2010 (Huber 2011) but not re-examined for the present study.

### ﻿Taxonomy and morphology

Taxonomic descriptions follow the style of recent publications on Pholcidae (e.g., Huber 2019; based on Huber 2000). Measurements were done on a dissecting microscope with an ocular grid and are in mm unless otherwise noted; eye measurements are +/- 5 µm. Photos were made with a Canon EOS 2000D digital camera mounted on a Nikon SMZ18 stereo microscope or a Nikon Coolpix 995 digital camera mounted on a Leitz Dialux 20 compound microscope. CombineZP (https://combinezp.software.informer.com/) was used for stacking photos. Drawings are partly based on photos that were traced on a light table and later improved under a dissecting microscope, or they were directly drawn with a Leitz Dialux 20 compound microscope using a drawing tube. Cleared female genitalia were stained with chlorazol black. The number of decimals in coordinates gives a rough indication of the accuracy of the locality data: four decimals means that the collecting site is within ~ 10 m of the indicated spot; three decimals: within ~ 100 m; two decimals: within ~ 1 km. The distribution maps were generated with ArcMap 10.0. For SEM photos, specimens were dried in hexamethyldisilazane (HMDS) (Brown 1993) and photographed with a Zeiss Sigma 300 VP scanning electron microscope. Abbreviations used in the text: **ALE** = anterior lateral eye(s); **ALS** = anterior lateral spinneret(s); **AME** = anterior median eye(s); **L/d** = length/diameter; **PLS** = posterior lateral spinnerets; **PME** = posterior median eye(s); **PMS** = posterior median spinneret(s).

### ﻿Molecular analysis

We newly generated CO1 barcodes of 19 specimens of *Micropholcus* (Table 1). To this we added previously published CO1 barcodes of eight further specimens of *Micropholcus* (from Astrin et al. 2006; Dimitrov et al. 2013; Eberle et al. 2018). We also included the barcode of “*Pholcus* sp.” from Dimitrov et al. (2008) because a separate phylogenetic analysis (pers. obs.; G. Meng, D. Dimitrov, L. Podsiadlowski, pers. comm. 26 July 2024) had suggested that this sequence is in fact based on a representative of *Micropholcus*. Finally, we added one outgroup species belonging to the closely related genus *Micromerys* Bradley, 1877 (from Eberle et al. 2018), and (for rooting the tree) *Artemabahla* Huber, 2019 (from Eberle et al. 2018).

**Table 1.** Geographic origins and GenBank accession numbers of specimens used in molecular analyses. Specimens are sorted as in Fig. 1. Newly sequenced barcodes are in bold.

Code	Genus and species	Vial	Country	Admin	Locality	Lat and Long	CO1	Source
S497	*Artemabahla*	Om33	Oman	Ad Dakhiliya	W of Bahla	22.9340, 57.0840	MG268735	Eberle et al. 2018
S297	*Micromerysyidin*	QMB8	Australia	Queensland	Kings Plains, Cooktown	-15.4850, 145.2560	MG268724	Eberle et al. 2018
GB37	*Micropholcusbaoruco*	DR/100-42	Dom. Rep.	Barahona	near Polo	18.1133, -71.2700	MG268886	Eberle et al. 2018
P0165	*Micropholcushispaniola*	DR/100-15	Dom. Rep.	La Vega	near La Ciénaga	19.0500, -70.8833	JX023558	Dimitrov et al 2013
Mic02	*Micropholcusdalei*	PuR002	Puerto Rico	Rio Grande	El Yunque, Big Trees Trail	18.3087, -65.7752	KF715606	Huber et al. 2014
P0193	*Micropholcusfauroti*	Cam129	Cameroon	Centre Region	Yaoundé	3.8833, 11.5233	JX023574	Dimitrov et al 2013
JA66	*Micropholcusfauroti*	G038	Cuba	La Habana	La Habana	23.1200, -82.4200	DQ667902	Astrin et al. 2006
UH448	*Micropholcuskhenifra*	Mor102	Morocco	Béni Mellal-Khénifra	near Sidi Ben Daoud	32.5347, -6.1285	**PQ066288**	NEW
S02	*Micropholcuskhenifra*	Mor103	Morocco	Béni Mellal-Khénifra	W of El Ksiba	32.5610, -6.0515	**PQ066277**	NEW
UH446	*Micropholcusghar*	Mor100	Morocco	Fès-Meknès	Kef el Ghar	34.4788, -4.2766	**PQ066290**	NEW
PUB6	*Micropholcus* sp.	-	Morocco	-	-	-	EU215669	Dimitrov et al. 2008
S321	*Micropholcusagadir*	Sieg11	Morocco	Souss-Massa-Draa	Agadir	30.4296, -9.6186	MG268754	Eberle et al. 2018
UH034	*Micropholcus* Br15-152	Br15-262	Brazil	Rio Grande do Norte	near Baraúna, Furna Feia cave	-5.0365, -37.5603	**PQ066285**	NEW
UH566	*Micropholcusbukidnon*	Phi250	Philippines	Mindanao	Barangay San Jose, Blue Water Cave	7.7060, 125.0320	**PQ066291**	NEW
S07	*Micropholcusbukidnon*	Phi250	Philippines	Mindanao	Barangay San Jose, Blue Water Cave	7.7060, 125.0320	**PQ066279**	NEW
GB38	*Micropholcuspiaui*	Carv1	Brazil	Piauí	Castelo do Piauí, Parque Municipal da Pedra de Castelo	-5.2017, -41.6875	MG268905	Eberle et al. 2018
UH036	*Micropholcus* Br16-23	Br16-241	Brazil	Pará	Serra Pelada	-5.9310, -49.6740	**PQ066286**	NEW
UH354	*Micropholcusevaluna*?	Ven20-178	Venezuela	Miranda	El Ávila National Park, near La Julia	10.5012, -66.8111	**PQ066287**	NEW
GB39	*Micropholcusubajara*	Carv17	Brazil	Ceará	Parque Nacional de Ubajara, Gruta de Morcego Branco	-3.8325, -40.8998	MG268847	Eberle et al. 2018
UH452	*Micropholcusshaat*	Om137	Oman	Dhofar	Shaat sinkhole	16.7740, 53.5870	**PQ066289**	NEW
S12	*Micropholcustanomah*	SA100	Saudi Arabia	Asir	NW of Tanomah	19.0220, 42.1247	**PQ066282**	NEW
S11	*Micropholcusbashayer*	SA96	Saudi Arabia	Asir	NW of Al Bashayer	19.8194, 41.8824	**PQ066276**	NEW
S09	*Micropholcusmaysaan*	SA88	Saudi Arabia	Al Bahah	NW of Al Bahah	20.2095, 41.3700	**PQ066284**	NEW
S20	*Micropholcusmaysaan*	SA140	Saudi Arabia	Mecca	NW of Maysaan	20.7717, 40.7985	**PQ066278**	NEW
UH565	*Micropholcusdarbat*	Om133	Oman	Dhofar	Wadi Darbat	17.0900, 54.4500	**PQ066292**	NEW
S05	*Micropholcusdarbat*	Om147	Oman	Dhofar	Ain Athoom	17.1185, 54.3667	**PQ066275**	NEW
S14	*Micropholcusabha*	SA111	Saudi Arabia	Asir	N of Abha	18.4168, 42.4646	**PQ066283**	NEW
S16	*Micropholcusharajah*	SA114	Saudi Arabia	Asir	SE of Harajah	17.8681, 43.3943	**PQ066281**	NEW
S15	*Micropholcusalfara*	SA112	Saudi Arabia	Asir	S of Al Fara	18.0487, 42.7096	**PQ066280**	NEW
S17	*Micropholcusdhahran*	SA121	Saudi Arabia	Asir	W of Dhahran Al Janub	17.7010, 43.3891	**PQ066274**	NEW

One or two legs of specimens stored in non-denatured pure ethanol (~ 99%) at -20 °C were used for DNA extraction. Extracted genomic DNA is deposited at and available from the LIB Biobank, Museum Koenig, Bonn. DNA was extracted and amplified as in Huber et al. (2024a). The CO1 primers used were LCO1490-JJ and HCO2198-JJ (Astrin et al. 2016; primer versions JJ2 served as backup). PCR products were sent for bidirectional Sanger sequencing to Macrogen (Amsterdam, The Netherlands).

CO1 barcode assembly, confirmation of source, and barcode alignment was as in Huber et al. (2024a). A neighbor-joining (NJ) tree (Saitou and Nei 1987) and genetic distances among specimens were calculated using the Kimura 2-parameter model (Kimura 1980) in MEGA 11 (Tamura et al. 2021), during which pairwise deletion of gaps in the alignment was applied. The NJ tree was assessed with 5000 bootstrap replications (Felsenstein 1985). The online tool iTOL (v. 6.9) (Letunic and Bork 2021; https://itol.embl.de/) was used for tree visualisation.

The online tool ASAP Web (https://bioinfo.mnhn.fr/abi/public/asap/; Puillandre et al. 2021) was used for species delimitation using the CO1 MSA. The Kimura 2-parameter (K2P) model (Kimura 1980) was applied for genetic distance calculation. Ten delimitation schemes with best scores (the smaller the better) were kept. At every stage of the process, ASAP groups objects (either a node or a specimen) within the same distance range into a node, each node at each stage has its own probability calculated. If a node’s probability falls below the specified threshold, ASAP will adjust the number of potential species, splitting any nodes with probabilities below the threshold (Puillandre et al. 2021). We used the default probability cutoff 0.01. Ten delimitation schemes with the best scores were kept.

## ﻿﻿Results

### ﻿﻿Molecular analysis

**Figure 1.**
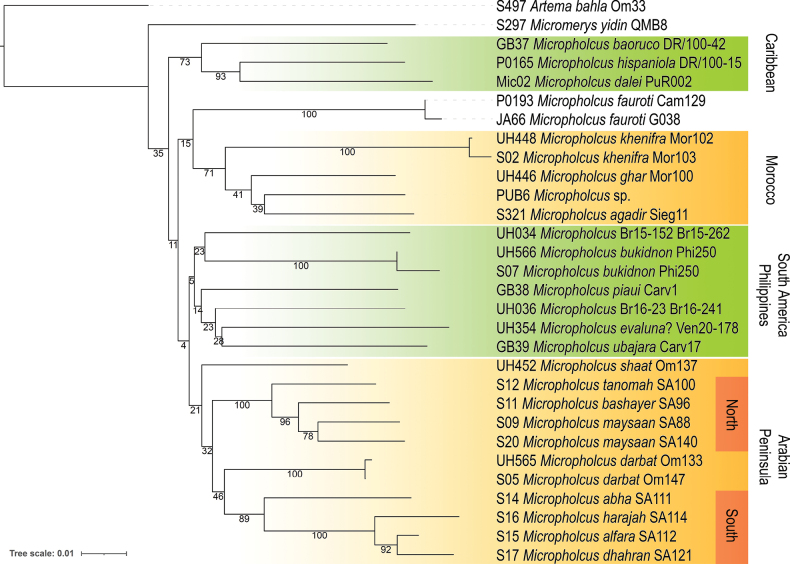
Neighbor-joining tree of analysed CO1 sequences using the Kimura 2-parameter model; numbers on the branches are bootstrap supports from 5000 replications (%). The labels North and South refer to two geographically and morphologically distinct species groups within Saudi Arabia.

**Figure 2.**
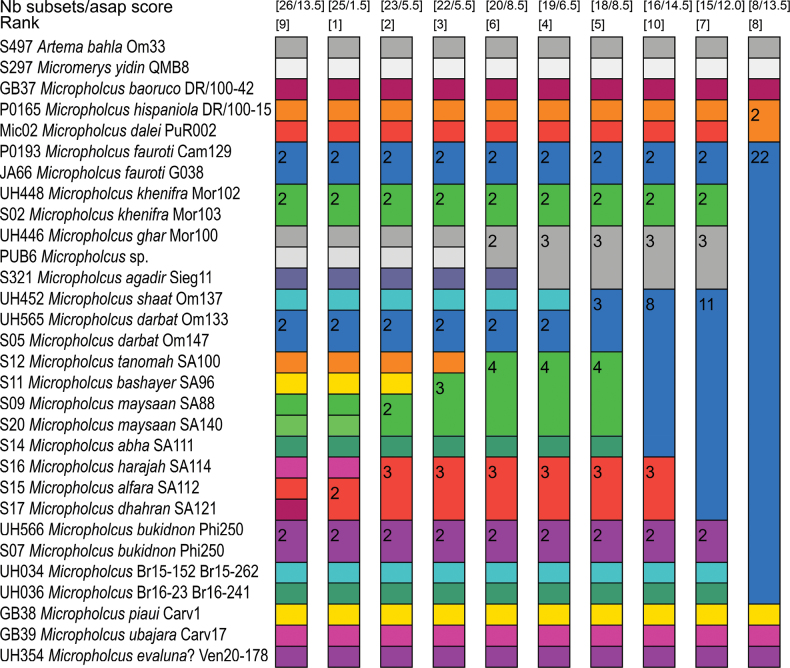
ASAP species delimitation analysis based on CO1 data. The analysis was performed on the web server https://bioinfo.mnhn.fr/abi/public/asap/. The Kimura 2-parameter model was applied. The ten best partitions are shown. The numbers for each column (partition) are: (1) Total number of species as identified by ASAP in the corresponding partition. (2) Score, an indicator of how good a partition is (the lower the score, the better the partition). (3) Rank of the scores. Note that the partition with the lowest (best) score splits *M.maysaan* sp. nov. into two species but joins *M.alfara* sp. nov. and *M.dhahran* sp. nov.

The CO1 NJ-tree (Fig. 1) is primarily intended to illustrate inter- and intraspecific distances. The exact individual values are shown in Table S1. Our sample of intraspecific distances is very low (5), and most distances are below 2%. Only the two sequenced specimens of *M.maysaan* sp. nov. have a higher distance, of 7.4%. This is also reflected in the ASAP analysis (Fig. 2), in which the partition with the best score splits *M.maysaan* sp. nov. into two species.

Interspecific distances within *Micropholcus* have a mean value of 19.8% (3.4%–25.7%). Only seven of the 371 distance values are at or below 10%. All of them refer to species within either the northern or southern Saudi Arabian groups. In the northern Saudi Arabian group, distances between *M.maysaan* sp. nov. and the other two species (*M.tanomah* sp. nov., *M.bashayer* sp. nov.) range from 8.0–10.0%. The distance between *M.tanomah* sp. nov. and *M.bashayer* sp. nov. is only slightly higher (10.2%). Despite these low values, the two partitions with the best scores in the ASAP analysis separate these three species.

In the southern Saudi Arabian group, *M.alfara* sp. nov. and *M.dhahran* sp. nov. are particularly close (3.4%); the distances of these two species to *M.harajah* sp. nov. are slightly higher (6.1–6.7%). Most partitions in the ASAP analysis resolve *M.alfara* sp. nov. and *M.dhahran* sp. nov. as a single species, and only the best (and the worst) resolve *M.harajah* sp. nov. as a distinct species.

### ﻿﻿Taxonomy

**Order Araneae Clerck, 1757**

**Family Pholcidae C.L. Koch, 1850**

#### ﻿﻿Genus Micropholcus

Taxon classificationAnimaliaAraneaePholcidae

Deeleman-Reinhold & Prinsen, 1987

81B7731B-688B-55EB-AF9F-FE4698E7EAA2

Micropholcus Deeleman-Reinhold & Prinsen, 1987: 73; type species: Pholcusfauroti Simon, 1887.
Micropholcus – Deeleman-Reinhold and van Harten 2001: 199. Huber 2011: 24. Huber et al. 2014: 435.
Mariguitaia González-Sponga, 2004: 66; type species: Mariguitaiadivergentis Gonzalez-Sponga 2004. Synonymised in Huber 2009.

##### Diagnosis.

**Figure 3.**
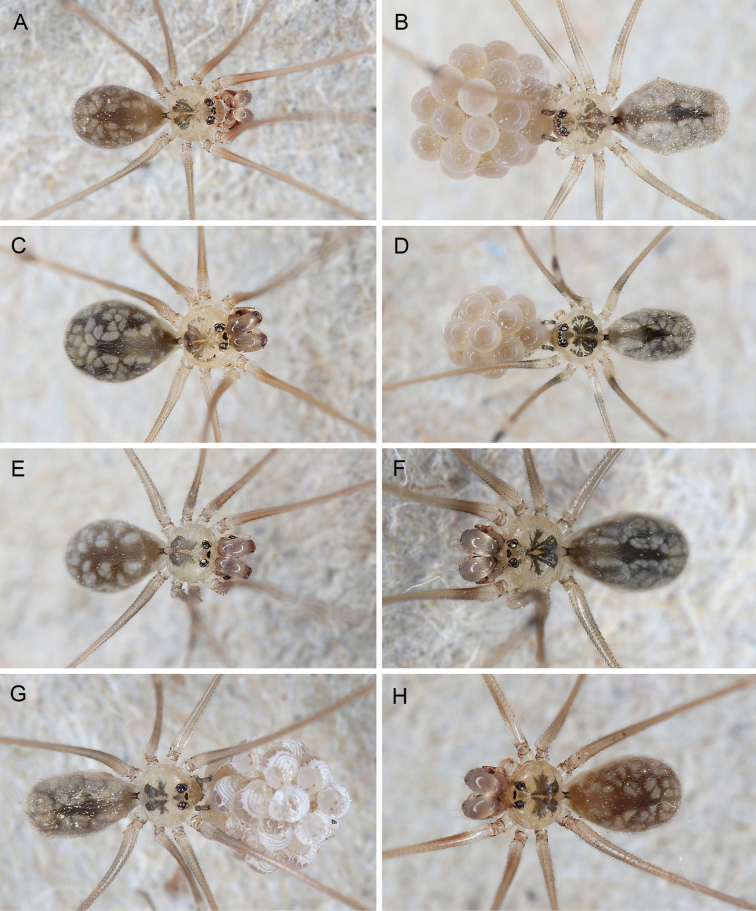
*Micropholcus* Deeleman-Reinhold & Prinsen; live specimens from Saudi Arabia **A***M.dhahran* Huber, sp. nov., male from ‘Asir, W of Dhahran Al Janub **B***M.harajah* Huber, sp. nov., female with egg-sac from ‘Asir, SE of Harajah **C, D***M.alfara* Huber, sp. nov., male and female with egg-sac from ‘Asir, S of Al Fara **E***M.abha* Huber, sp. nov., male from ‘Asir, N of Abha **F***M.tanomah* Huber, sp. nov., male from ‘Asir, NW of Tanomah **G***M.bashayer* Huber, sp. nov., female with egg-sac from ‘Asir, NW of Al Bashayer **H***M.maysaan* Huber, sp. nov., male from Mecca, NW of Maysaan. Photographs BAH.

**Figure 4.**
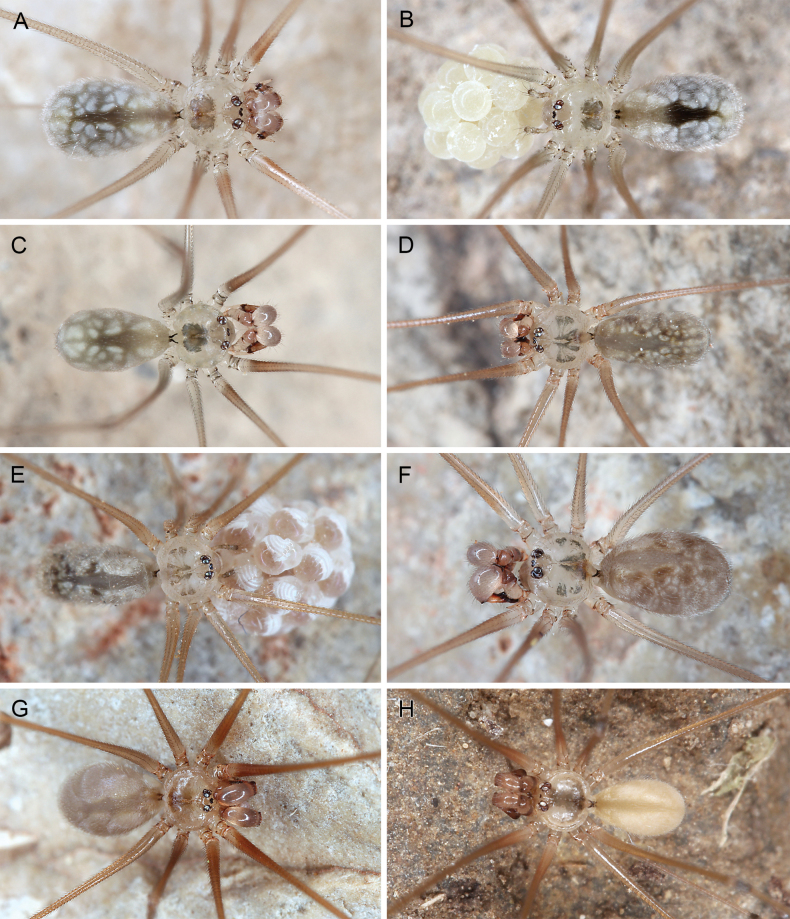
*Micropholcus* Deeleman-Reinhold & Prinsen; live specimens from Oman, Morocco, and the Philippines **A, B***M.darbat* Huber, sp. nov., male and female with egg-sac from Oman, Dhofar, near Qairoon Hairitti **C***M.shaat* Huber, sp. nov., male from Oman, Dhofar, Shaat sinkhole **D, E***M.agadir* (Huber), male and female with egg-sac from Morocco, Souss-Massa, Paradise Valley **F***M.ghar* Huber, sp. nov., male from Morocco, Fès-Meknès, Kef El Ghar **G***M.khenifra* Huber, Lecigne & Lips, sp. nov., male from Morocco, Béni Mellal-Khénifra, near Sidi Ben Daoud **H***M.bukidnon* Huber, sp. nov., male from Philippines, Mindanao, Blue Water Cave. Photographs BAH.

Old World species are long-legged, eight-eyed pholcids with an oval abdomen (Figs 3, 4); New World species are more variable, sometimes without AME, sometimes with elongate to worm-shaped abdomens. Most known species (except *M.bukidnon* sp. nov.) with unique modified hair at tip of male palpal trochanter apophysis (Fig. 9A–D; see also Huber 2000: figs 105, 106; Huber and Wunderlich 2006: figs 4h, 8d; Huber 2011: fig. 95; Huber et al. 2014: figs 32, 57). Male chelicerae with frontal apophyses with modified hairs (Fig. 6), i.e., similar to putative sister genus *Cantikus* Huber but different from other close relatives (*Leptopholcus* Simon, *Pehrforsskalia* Deeleman-Reinhold & van Harten, and *Micromerys* Bradley). Procursus and bulb morphology highly variable and not diagnostic at genus level (contra Huber 2011). Females not diagnosable morphologically at genus level (highly variable and similar to closely related genera).

##### Note.

Most parts in this general section about *Micropholcus* refer to the entire genus. The following description is limited to Old World taxa because they are relatively homogeneous, while some New World species (in particular those on the Caribbean islands) are superficially extremely different from South American (in particular Brazilian) and Old World species.

##### Description

**(Old World taxa). Male. *Measurements*.** Total body length ~ 2.3–3.9. Carapace width 0.8–1.5. Diameter PME 60–100 µm; diameter AME usually 35–55 µm, in *M.bukidnon* sp. nov. only 15 µm. Tibia 1 length 5.0–10.2. Tibia 1 L/d: 57–85. Leg formula 1243. Diameters of leg femora (at half length) 80–150 µm, of leg tibiae 75–120 µm.

***Colour*** (in ethanol). Prosoma and legs pale ochre-yellow to grey, carapace with dark pattern, legs with darker patellae and tibia-metatarsus joints; abdomen ochre-grey to whitish, monochromous or with whitish marks. Live specimens (Figs 3, 4) similar in colour but slightly darker.

**Figure 5.**
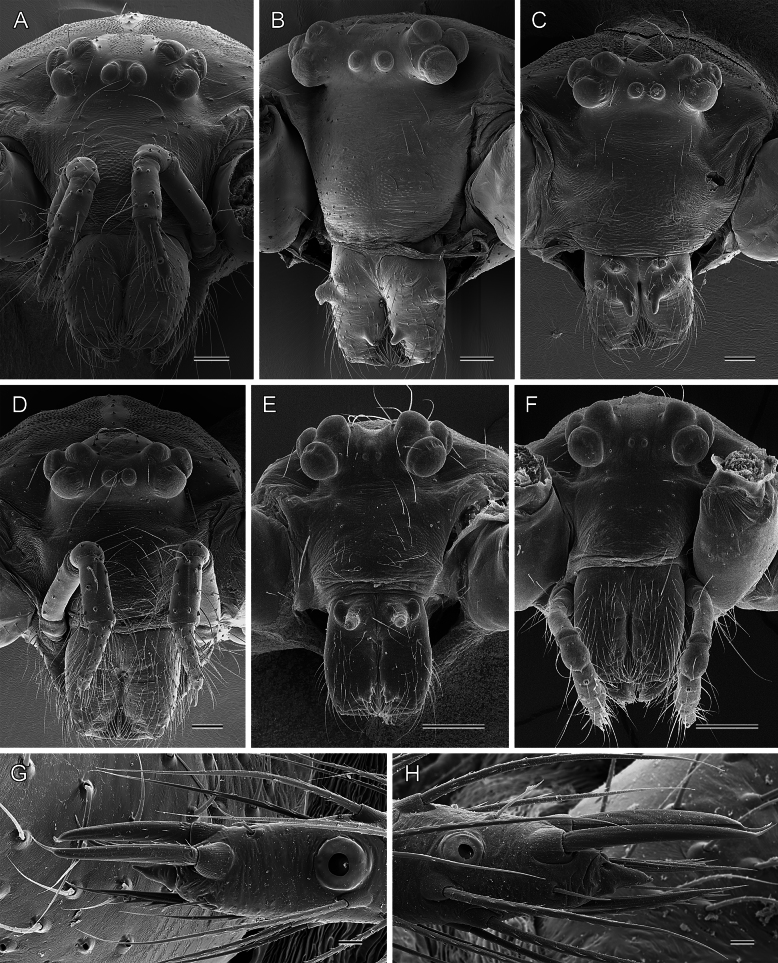
*Micropholcus* Deeleman-Reinhold & Prinsen; SEM images of prosomata (frontal views) and tips of female palps (dorsal views) **A***M.tanomah* Huber, sp. nov.; female **B***M.darbat* Huber, sp. nov.; male **C, D***M.ghar* Huber, sp. nov.; male and female **E, F***M.bukidnon* Huber, sp. nov.; male and female; note small AME**G***M.tanomah* Huber, sp. nov., left palp **H***M.ghar* Huber, sp. nov., right palp. Scale bars: 100 µm (**A–D**); 200 µm (**E, F**); 10 µm (**G, H**).

***Body*.** Ocular area slightly raised (distinct in frontal view; Fig. 5). Carapace without thoracic groove. Clypeus unmodified. Sternum slightly wider than long, unmodified. Abdomen approximately twice as long as wide. Gonopore with four (rarely five) epiandrous spigots (Fig. 10; see also Huber 2000: fig. 123; Huber 2011: fig. 99). ALS with one strongly widened spigot, one long pointed spigot, and six cylindrical spigots (Fig. 9; see also Huber 2000: figs 158, 159; Huber 2011: fig. 100); PMS with two conical spigots; PLS without spigots.

**Figure 6.**
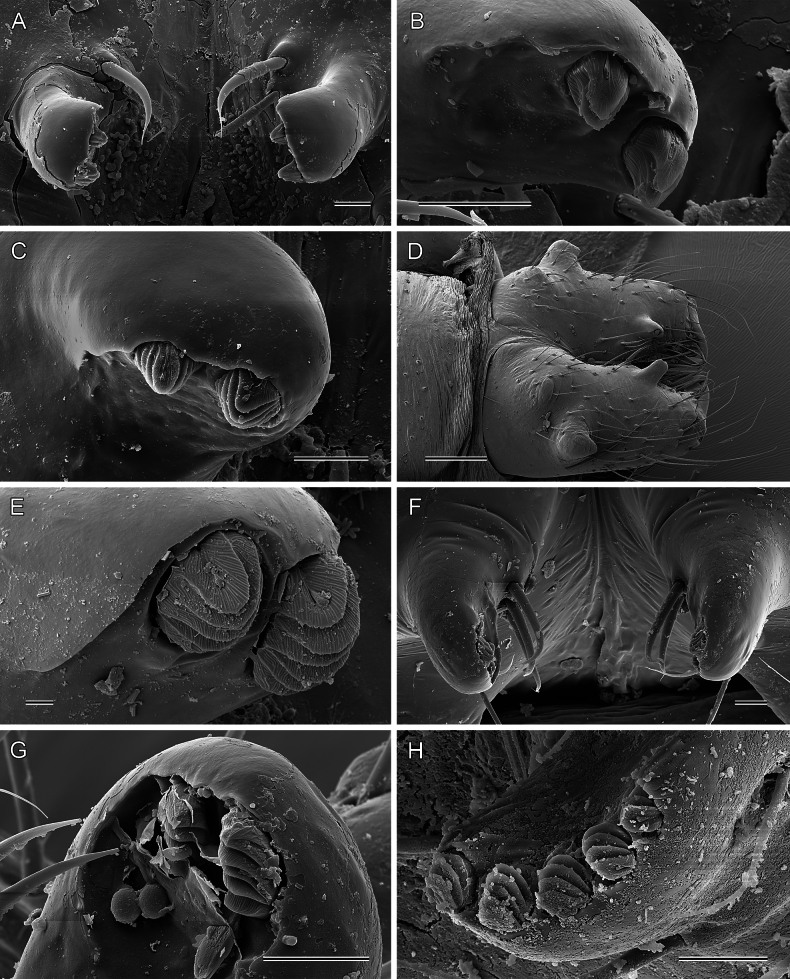
*Micropholcus* Deeleman-Reinhold & Prinsen; SEM images of male chelicerae: distal apophyses with modified hairs and total view (**D**) **A, B***M.alfara* Huber sp. nov. **C***M.tanomah* Huber, sp. nov. **D, E***M.darbat* Huber, sp. nov. **F, G***M.ghar* Huber, sp. nov. **H***M.bukidnon* Huber, sp. nov. Scale bars: 10 µm (**A–C, F–H**); 100 µm (**D**); 2 µm (**E**).

***Chelicerae*.** Chelicerae with pair of strong frontal apophyses provided with conical or globular, strongly sculptured modified hairs (Fig. 6; see also Huber 2011: fig. 98), usually with one or two pairs of proximal processes; without stridulatory files.

**Figure 7.**
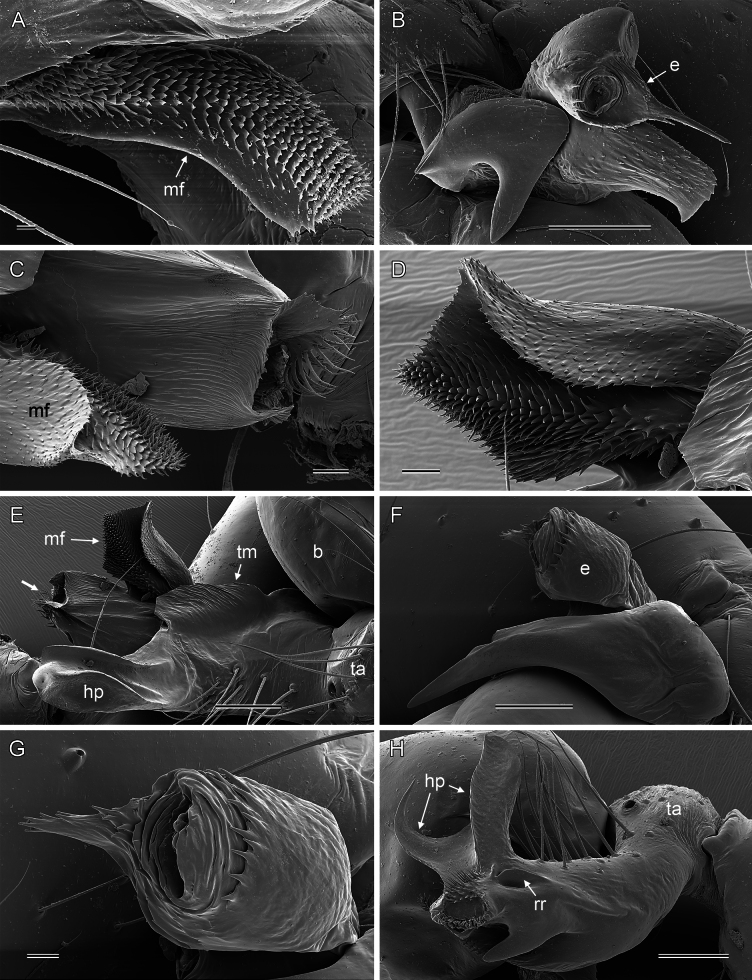
*Micropholcus* Deeleman-Reinhold & Prinsen; SEM images of male palpal structures **A, B***M.alfara* Huber, sp. nov.; prolateral membranous flap on left procursus and right bulbal processes, prolateral view **C, D***M.tanomah* Huber, sp. nov., tip of left procursus and prolateral membranous flap on left procursus **E***M.tanomah* Huber, sp. nov., left procursus, dorsal view (bold arrow points at tip of procursus) **F, G***M.tanomah* Huber, sp. nov., left bulbal processes, prolateral view, and embolus of same palp in slightly more distal view **H***M.darbat* Huber, sp. nov., left procursus, retrolateral view. Abbreviations: b, genital bulb; e, embolus; hp, dorsal hinged process; mf, membranous prolateral flap; rr, retrolateral ridge; ta, tarsus; tm, transparent membrane. Scale bars: 10 µm (**A**); 100 µm (**B, E, F, H**); 20 µm (**C, D, G**).

**Figure 8.**
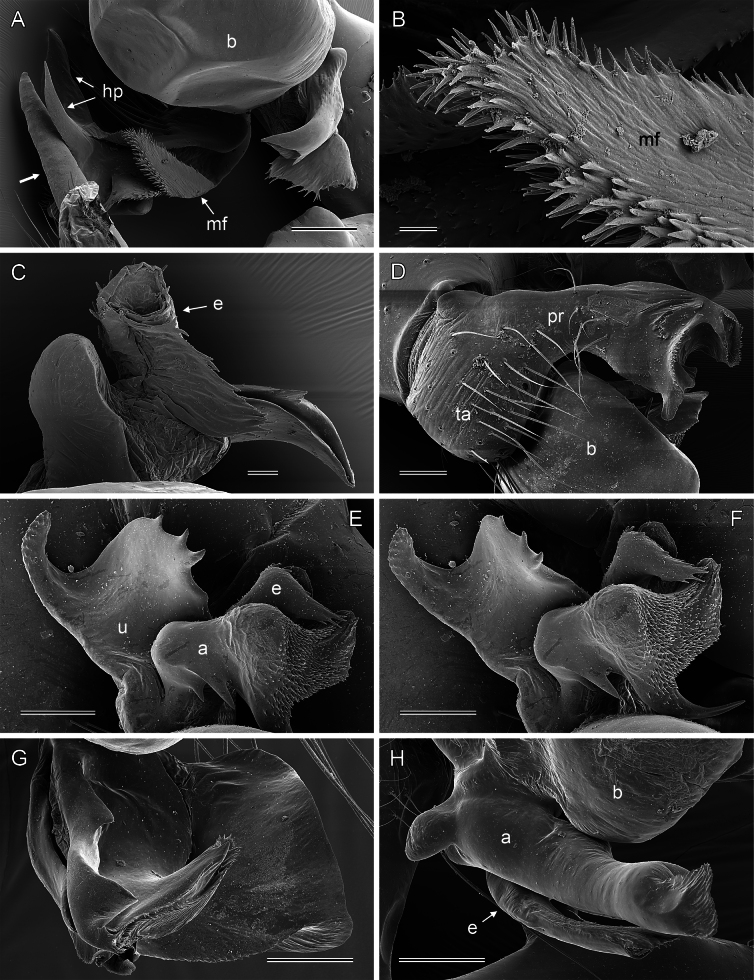
*Micropholcus* Deeleman-Reinhold & Prinsen; SEM images of male palpal structures **A, B***M.darbat* Huber, sp. nov.; right bulb and procursus, prolateral view (bold arrow in A points at trochanter apophysis), and prolateral membranous flap of procursus at higher magnification **C***M.darbat* Huber, sp. nov., left bulbal processes **D***M.ghar* Huber, sp. nov., left procursus, retrolateral view **E, F***M.ghar* Huber, sp. nov., right bulbal processes, prolateral and prolateral-ventral views **G***M.bukidnon* Huber, sp. nov., left procursus, prolateral-distal view **H***M.bukidnon* Huber, sp. nov., left bulbal processes, prolateral distal view. Abbreviations: a, putative appendix; b, genital bulb; e, embolus; hp, dorsal hinged process; mf, membranous prolateral flap; pr, procursus; ta, tarsus; u, putative uncus. Scale bars: 100 µm (**A, D–H**); 10 µm (**B**); 20 µm (**C**).

**Figure 9.**
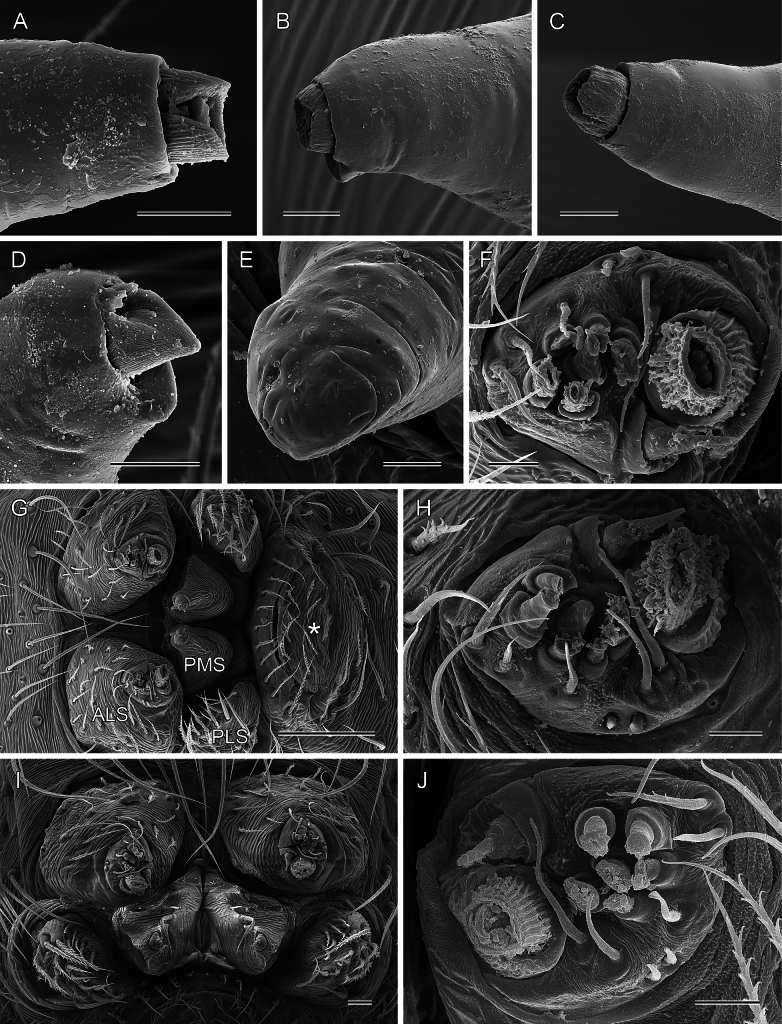
*Micropholcus* Deeleman-Reinhold & Prinsen; SEM images of male palpal trochanter tips and of spinnerets **A***M.alfara* Huber, sp. nov. **B***M.tanomah* Huber, sp. nov. **C***M.darbat* Huber, sp. nov. **D***M.ghar* Huber, sp. nov. **E***M.bukidnon* Huber, sp. nov. **F, G***M.tanomah* Huber, sp. nov., male ALS, and male spinnerets and anal cone (asterisk) **H, I***M.ghar* Huber, sp. nov., male ALS and male spinnerets **J***M.bukidnon* Huber, sp. nov., male ALS. Scale bars: 10 µm (**A–F, H, J**), 100 µm (**G**), 20 µm (**I**).

***Palps*.** Palpal coxa unmodified. Trochanter with retrolateral-ventral apophysis usually with distinctive modified (short cylindrical) hair at tip (Fig. 9A–D), modified hair absent in *M.bukidnon* sp. nov. Femur variable in shape, often with rounded processes (usually ventrally and retrolaterally, sometimes also dorsally). Femur-patella joints shifted towards prolateral side. Tibia larger than femur, with two trichobothria. Tibia-tarsus joints shifted towards retrolateral side. Palpal tarsus with large capsulate tarsal organ (Fig. 11H, I; see also Huber 2011: fig. 97), outer diameter 30–35 µm, diameter of opening 15–20 µm; more open (almost exposed) in *M.bukidnon* sp. nov. (Fig. 11J). Procursus complex, often with distinct dorsal hinged process (e.g., Figs 16C, 43C, 52C), in most species with transparent prolateral membranous flap densely set with teeth (Figs 7A, D, 8B). Genital bulb with distinct proximal sclerite connecting to tarsus, membranous or partly sclerotised embolus, and variably complex set of sclerotised apophyses, sometimes likely homologues of the *Pholcus* ‘appendix’ and ‘uncus’, sometimes of uncertain homology (Figs 7B, F, 8C, E, F, H).

***Legs*.** Without spines and curved hairs. Without slender metatarsal hairs (cf. Huber et al. 2023a). Without sexually dimorphic short vertical hairs. Chemoreceptive hairs ~ 20–25 µm long, with few side branches (Fig. 12A, H), mostly near leg tips. Retrolateral trichobothrium of tibia 1 at 5–10% of tibia length. Prolateral trichobothrium absent on tibia 1, present on tibiae 2–4. Base of trichobothria evenly rounded, without proximal ridge (cf. Fig. 11D). Legs with roundish cuticular plates (Fig. 12E; diameter ~ 6–8 µm) and rimmed pores (Fig. 12B; outer diameter 2 µm, diameter of opening 0.2 µm) apparently on all leg segments. Tarsus 1 with ~ 20–30 pseudosegments, distally usually fairly distinct. Leg tarsal organs capsulate (Figs 11E–G, 12F; diameter 12–17 µm, diameter of opening 5–7 µm). Tarsus 4 with single row of comb-hairs on prolateral side (Fig. 12G, H; see also Huber 2011: fig. 96). Main tarsal claws with ~ 10 teeth (Fig. 12 C, D).

**Figure 10.**
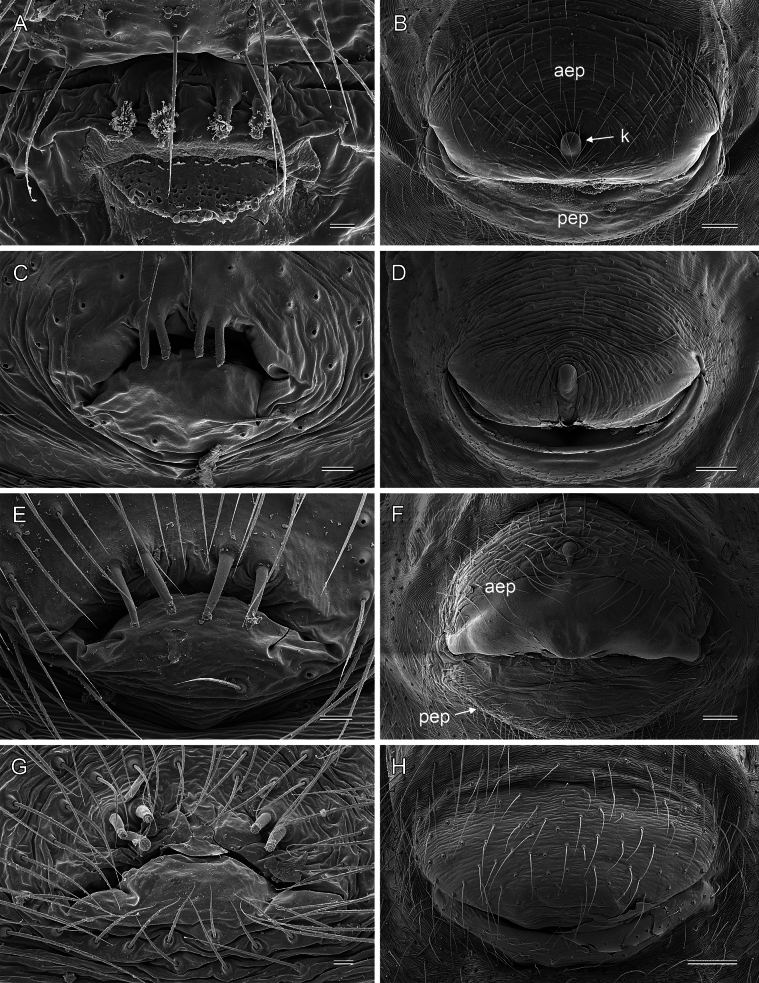
*Micropholcus* Deeleman-Reinhold & Prinsen; SEM images of male gonopores with epiandrous spigots and of female epigyna **A, B***M.tanomah* Huber, sp. nov. **C, D***M.darbat* Huber, sp. nov. **E, F***M.ghar* Huber, sp. nov. **G, H***M.bukidnon* Huber, sp. nov. Abbreviations: aep, anterior epigynal plate; k, epigynal ‘knob’; pep, posterior epigynal plate. Scale bars: 10 µm (**A, G**); 100 µm (**B, D, F, H**); 20 µm (**C, E**).

**Figure 11.**
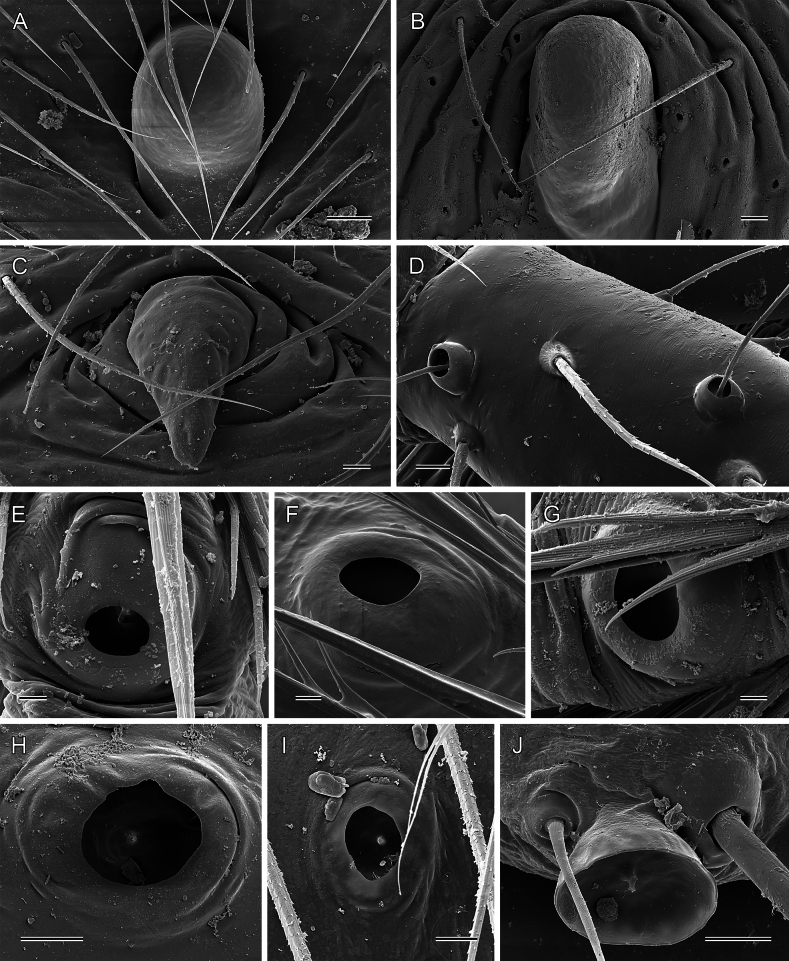
*Micropholcus* Deeleman-Reinhold & Prinsen; SEM images of epigynal knobs (**A–C**), trichobothria (**D**), and tarsal organs (**E–J**) **A***M.tanomah* Huber, sp. nov. **B***M.darbat* Huber, sp. nov. **C***M.ghar* Huber, sp. nov. **D***M.alfara* Huber, sp. nov., female left palpal tibia **E***M.alfara* Huber, sp. nov., male right tarsus 2 **F***M.tanomah* Huber, sp. nov., female left tarsus 2 **G, H***M.darbat* Huber, sp. nov., female right tarsus 2 and male palpal tarsus **I***M.ghar* Huber, sp. nov., male palpal tarsus **J***M.bukidnon* Huber, sp. nov., male palpal tarsus. Scale bars: 20 µm (**A**); 10 µm (**B–D, H–J**); 2 µm (**E–G**).

**Figure 12.**
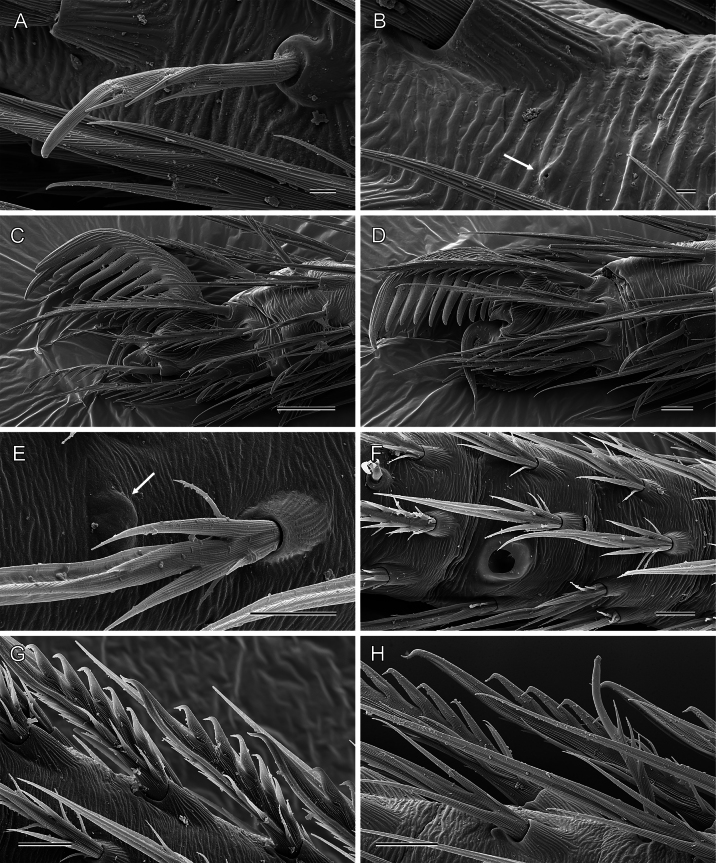
*Micropholcus* Deeleman-Reinhold & Prinsen, SEM images of leg structures **A***M.tanomah* Huber, sp. nov., putative chemoreceptor **B***M.tanomah* Huber, sp. nov., rimmed pore (arrow) on left tarsus 3 **C, D***M.tanomah* Huber, sp. nov., tarsal claws of left legs 1 and 3 **E***M.ghar* Huber, sp. nov., cuticular plate (arrow) and regular mechanoreceptor on right metatarsus 3 **F***M.ghar* Huber, sp. nov., pseudosegmentation (and tarsal organ) of right tarsus 3 **G***M.ghar* Huber, sp. nov., comb-hairs on male tarsus 4 **H***M.bukidnon* Huber, sp. nov., comb-hairs on male tarsus 4. Scale bars: 2 µm (**A**); 1 µm (**B**); 20 µm (**C**); 10 µm (**D–H**).

**Figure 13.**
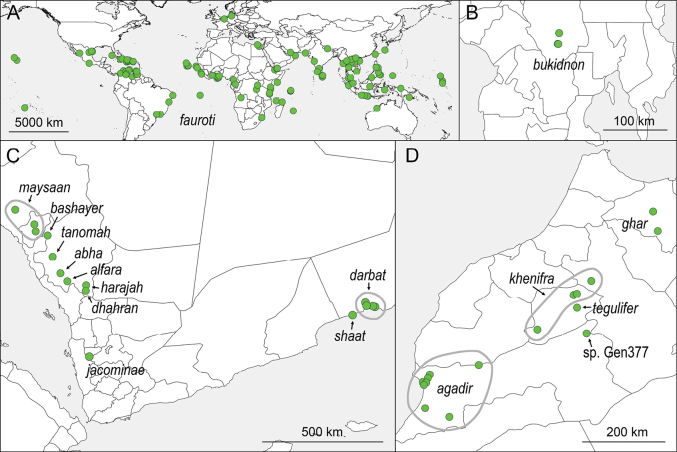
Known distribution of *Micropholcusfauroti* (Simon) (**A**) and of Old World *Micropholcus* in Mindanao (**B**), on the Arabian Peninsula (**C**) and in Morocco (**D**).

**Female.** In general, very similar to males (Figs 3, 4). Legs slightly shorter than in males (male / female tibia 1 length: ~ 1.1–1.4, but sample sizes mostly small); tibia 1 length 4.3–9.1. Palpal tarsal organ smaller than in males (outer diameter: 18–25 µm, diameter of opening: 6–10 µm). Palpal tarsus ending distally in pointed tip and pair of strong dorsal hairs (Fig. 5G, H). Spinnerets, leg hairs, cuticular plates, rimmed pores, comb-hairs, leg tarsal organs, and tarsal claws as in male. Epigynum anterior plate usually weakly sclerotised, with rounded process (‘knob’; Fig. 11A–C) in varying position; posterior epigynal plate short and indistinct. Internal genitalia often complex, highly variable, with distinct pair of pore plates.

##### Distribution.

The type species *Micropholcusfauroti* has attained a circumtropical distribution, with most records from between 25°S and 30°N (Fig. 5A).

*Micropholcus* is one of only two Pholcidae genera (together with *Pholcus*) with autochthonous species in both the Old and New Worlds. New World species are mostly known from the Greater Antilles and from semi-arid regions in Brazil; the genus seems to be largely absent from the humid regions of the Amazon basin. Old World species are currently known from the Arabian Peninsula, Morocco, and the Philippines (Fig. 5B–D). Specimens have been collected from sea level to 2370 m. In Saudi Arabia, all new species described herein were collected above 1200 m. Several of the localities visited below 1000 m had suitable habitats but no *Micropholcus*.

##### Natural history.

**Figure 14.**
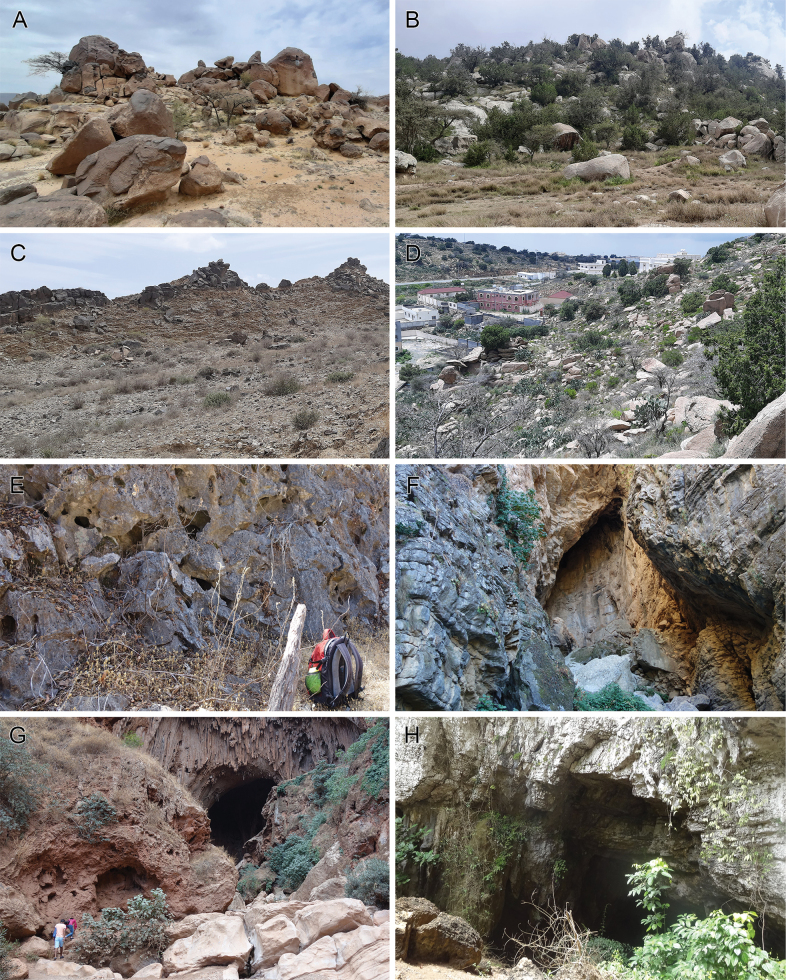
Typical habitats of *Micropholcus* Deeleman-Reinhold & Prinsen in the Old World **A** Saudi Arabia, ‘Asir, SE of Harajah (type locality of *M.harajah* Huber, sp. nov.) **B** Saudi Arabia, ‘Asir, S of Al Fara (type locality of *M.alfara* Huber, sp. nov.) **C** Saudi Arabia, ‘Asir, N of Abha (type locality of *M.abha* Huber, sp. nov.) **D** Saudi Arabia, Mecca, NW of Maysaan (type locality of *M.maysaan* Huber, sp. nov.) **E** Oman, Dhofar, near Shaat sinkhole (type locality of *M.shaat* Huber, sp. nov.) **F** Morocco, Fès-Meknès, Kef El Ghar (type locality of *M.ghar* Huber, sp. nov.) **G** Morocco, Béni Mellal-Khénifra, Imi n’Ifri (*M.khenifra* Huber, Lecigne & Lips, sp. nov.) **H** Philippines, Mindanao, Kabyaw Cave (*M.bukidnon* Huber, sp. nov.). Photos BAH.

Old World *Micropholcus* seem to be very homogeneous with respect to their preferred microhabitats. Most species have been collected from rocks: in caves and at cave entrances, in small caverns of rock walls, and on the undersides of large boulders (Fig. 14). They share this type of microhabitat with most South American species, and with the majority of species of the putative sister genus (*Cantikus*), suggesting that this might be the plesiomorphic microhabitat. The unusual microhabitat reported for *M.jacominae* (dry plant debris in an irrigated banana plantation) needs confirmation. The synanthropic *M.fauroti* is usually found in the upper corners and edges of rooms. Caribbean species have shifted to the undersides of leaves, probably explaining their significantly different general body shape and colour (see Huber and Wunderlich 2006; Huber et al. 2014).

**Figure 15.**
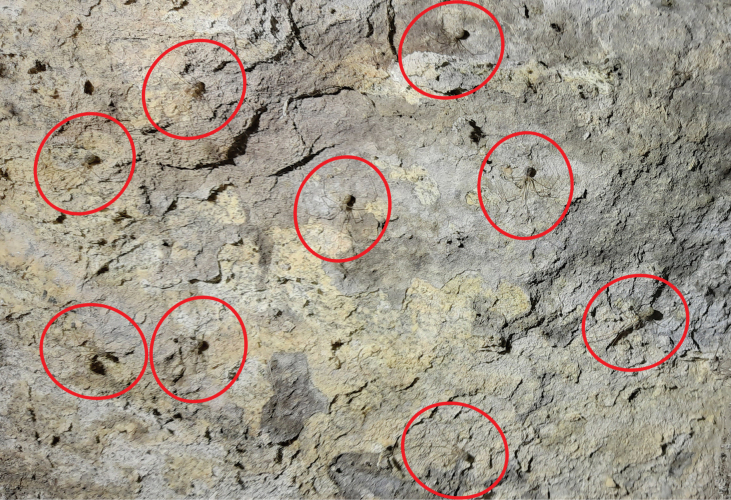
Section of cave ceiling (16 × 11 cm), showing nine adult specimens of *M.tanomah* Huber, sp. nov.; from NW of Tanomah, ‘Asir, Saudi Arabia.

Old World and Brazilian *Micropholcus* spiders build fine dome-shaped webs but during the day, most species (except those deeper in caves, e.g., *M.ghar* sp. nov.) sit flat on the rock surface (Fig. 15). The webs of leaf-dwelling Caribbean species have not yet been described. Upon disturbance, the spiders show a range of reactions, from refusing to move, bouncing, walking or running away, to dropping out of the web. Egg sacs are round (in Caribbean species elongated), covered by a barely visible sparse layer of silk (Figs 3, 4), and contain up to ~ 35 eggs; egg diameters range from 0.54 to 0.71 mm. For more detailed observations on Brazilian species, see Huber et al. (2014). For detailed life history data of *M.fauroti* under lab conditions see Ahmad and Abou-Setta (2017). For anecdotal observations on further Old World species, see individual species descriptions below.

##### Relationships.

The molecular analysis of Eberle et al. (2018) placed *Micropholcus* as sister to the South-East Asian genus *Cantikus* Huber, 2018, within a clade that included three further Old World genera: *Leptopholcus* Simon, 1893; *Pehrforsskalia* Deeleman-Reinhold & van Harten, 2001; and *Micromerys* Bradley, 1877. That analysis included ten species of *Micropholcus*: the type species *M.fauroti*, two further Old World species (*M.agadir* and *M.bukidnon* sp. nov.) and seven New World species. The monophyly of the genus received maximum support. New World species were nested within a paraphyletic Old World group.

Our NJ tree (Fig. 1) is not expected to reliably reflect phylogenetic relationships. However, some clades that receive reasonable to high support are either congruent with the results of Eberle et al. (2018) (Caribbean clade) or include geographically neighbouring species: the Moroccan clade, the southern Saudi Arabian clade, and the northern Saudi Arabian clade. The latter two are also supported by several morphological similarities each, but it is not clear which of these are synapomorphies and which not. Thus, relationships within *Micropholcus* are largely unresolved and Fig. 1 should not be misinterpreted in a phylogenetic context.

##### Composition.

The genus now includes 30 described species: the Dominican amber fossil *M.kiskeya* (Huber & Wunderlich, 2006) and 29 extant species. Of the latter, seven occur in South America, six on the Caribbean islands, and 16 in the Old World. All Old World species are treated below except for *M.tegulifer* Barrientos, 2019 (a loan request was denied by the curator of arthropods, Museu de Ciències Naturals de Barcelona). Numerous undescribed New World species are available in collections, in particular from Brazil (L.S. Carvalho, pers. comm. 2 July 2020). At least one further undescribed species is known to occur in Morocco, represented by a single male specimen deposited in the Muséum d’histoire naturelle, Genève, Switzerland (“sp. Gen377” in Fig. 13D). It resembles *M.tegulifer* but has a very different uncus. It originates from the Gorges du Dades area in the Drâa-Tafilalet Region, ~ 31.535°N, 5.918°W. Our molecular data indicate that the “*Pholcus* sp.” CO1 barcode published in Dimitrov et al. (2008) is also from a *Micropholcus*, different from *M.agadir*, *M.ghar* sp. nov., and *M.khenifra* sp. nov. (Figs 1, 2). It could be *M.tegulifer*, *M.* sp. Gen377, or a different new species. Its geographic origin cannot be reconstructed, and the only available (juvenile) specimen is lost (D. Dimitrov and C. Ribera, pers. comm. 20 Mar. and 19 Apr. 2024).

#### ﻿﻿ Micropholcus fauroti

Taxon classificationAnimaliaAraneaePholcidae

(Simon, 1887)

A9029B97-B4FE-59F4-BCD3-6BEB888A7F05

Figs 16 , 17 , 18

##### Notes.

For synonymy, type information, and redescription, see Huber (2011). Numerous further records and an updated distribution map were published in Huber et al. (2017). Since then, new records have been published for Egypt (Ahmad and Abou-Setta 2017), Sri Lanka (Huber 2019), Venezuela (Huber and Villarreal 2020) and India (Vishnudas and Sudhikumar (2021). The map in Fig. 13A summarises all the previous records plus the new records below.

**Figure 16.**
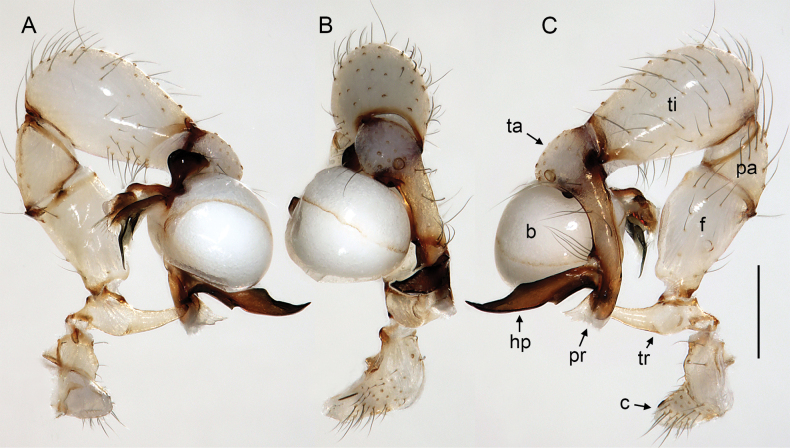
*Micropholcusfauroti* (Simon, 1887); male from Mexico, Nuevo León, Santiago (ZFMK Ar 24655). Left palp in prolateral (**A**), dorsal (**B**), and retrolateral (**C**) views. Abbreviations: b, genital bulb; c, coxa; f, femur; hp, dorsal hinged process; pa, patella; pr, procursus; ta, tarsus; ti, tibia; tr, trochanter. Scale bar: 0.3 mm.

##### New records.

Colombia: **La Guajira** • 3 ♂♂, 5 ♀♀; Palomino; 11.2451°N, 73.5619°W; 10 m a.s.l.; in building; 17 Sep. 2022; B.A. Huber leg.; ZFMK Ar 24653.

Mexico: **Guerrero** • 1 ♂, 3 ♀♀; Coyuca de Benitez; 17.0075°N, 100.0893°W; 20 m a.s.l.; in building; 3 Oct. 2019; B.A. Huber leg.; ZFMK Ar 24654. **Nuevo León** • 1 ♂, 8 ♀♀, 1 juv.; Santiago; 25.4237°N, 100.1463°W; 450 m a.s.l.; in building; 14 Oct. 2019; B.A. Huber leg.; ZFMK Ar 24655 • 5 ♂♂, 5 ♀♀, in pure ethanol (one male palp and two female abdomens transferred to ZFMK Ar 24655); same collection data as for preceding; ZFMK Mex286.

United Arab Emirates: **Dubai** • 2 ♂♂, 1 ♀ (“Micropholcuscf.fauroti” in Feulner and Roobas 2016); Dubai, near Emirates Towers; 25.219°N, 55.282°E; 5 m a.s.l.; 3 Jun. 2015; G.R. Feulner leg.; ZFMK Ar 24680.

Oman: **Ash Sharqiyah South** • 3 ♂♂, 5 ♀♀, 2 juvs; Wadi Tiwi; 22.801°N, 59.240°E; on banana leaves; 60 m a.s.l.; 22 Mar. 2017; B.A. Huber leg.; ZFMK Ar 24656 • 1 ♂, 2 juvs, in pure ethanol; same collection data as for preceding; ZFMK Om27.

##### Diagnosis.

**Figure 17.**
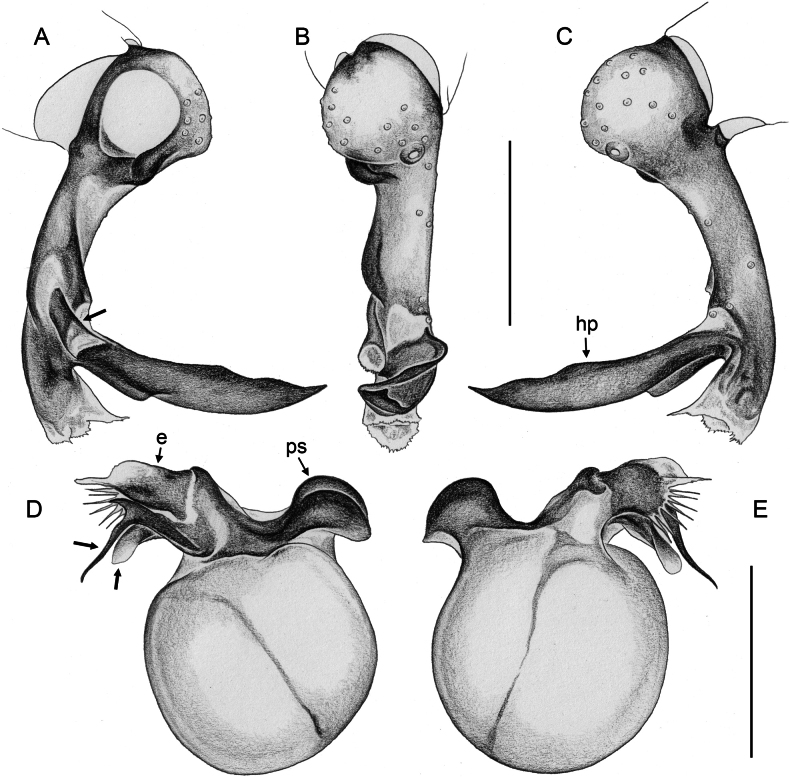
*Micropholcusfauroti* (Simon, 1887); male from Mexico, Nuevo León, Santiago (ZFMK Ar 24655) **A–C** left procursus in prolateral, dorsal, and retrolateral views; arrow in A points at distinctive prolateral process **D, E** left genital bulb in prolateral and retrolateral views; bold arrows in D point at distinctive processes of unclear homology. Abbreviations: e, embolus; hp, dorsal hinged process; ps, proximal bulbal sclerite. Scale bars: 0.3 mm.

**Figure 18.**
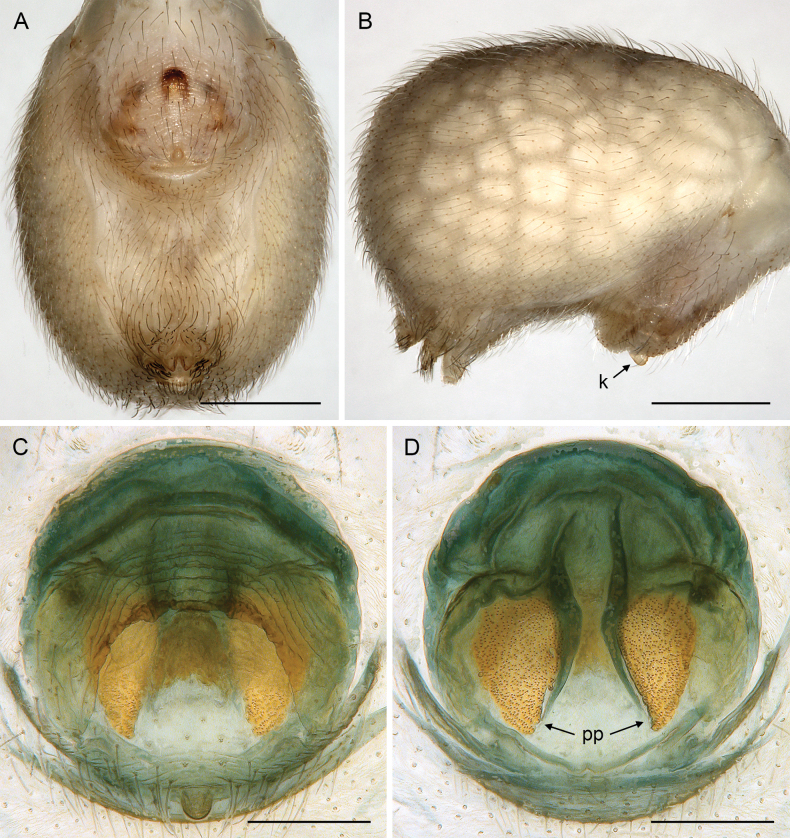
*Micropholcusfauroti* (Simon, 1887); female from Mexico, Nuevo León, Santiago (ZFMK Ar 24655) **A, B** abdomen, ventral and lateral views **C, D** cleared genitalia, ventral and dorsal views. Abbreviations: k, epigynal ‘knob’; pp, pore plate. Scale bars: 0.5 mm (**A, B**); 0.2 mm (**C, D**).

Males are easily distinguished from known congeners by long and slender dorsal hinged process on procursus (Fig. 17C; similar but relatively shorter in some Saudi Arabian species, cf. Figs 24C, 40C, etc.); also by unique prolateral process on procursus (arrowed in Fig. 17A) and by unique shapes of processes of genital bulb (Fig. 17D, E). Females are distinguished by distinct U-shaped internal structure visible through epigynal plate in uncleared specimens (Fig. 18A); similar dark internal structures occur in some Saudi Arabian species (e.g., Figs 26A, 30A); also by very large pore plates and large anterior membranous element of internal genitalia (Fig. 18D).

#### ﻿﻿ Micropholcus jacominae

Taxon classificationAnimaliaAraneaePholcidae

Deeleman-Reinhold & van Harten, 2001

0F814DD4-2AED-5AD9-9433-8E32E18DD5E8

**Figure 19.**
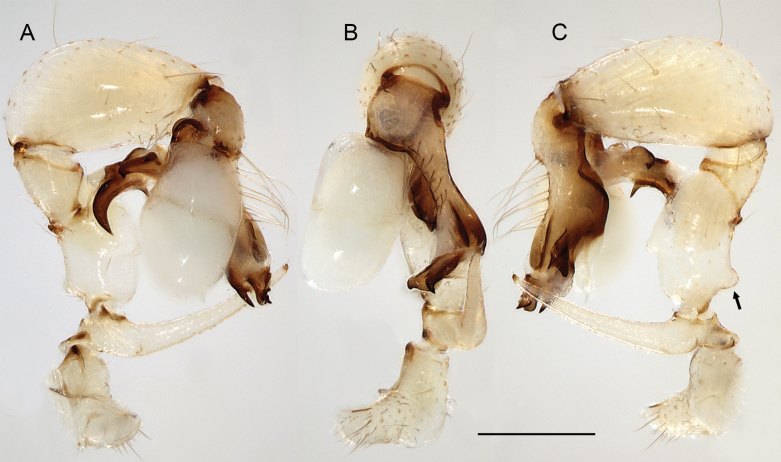
*Micropholcusjacominae* Deeleman-Reinhold & van Harten, 2001; male paratype from Yemen, Al Mahwit, Khamis Bani Sa’d (RMNH). Left palp in prolateral (**A**), dorsal (**B**), and retrolateral (**C**) views; arrow in C points at distinctive dorsal process on femur. Scale bar: 0.3 mm.

**Figure 20.**
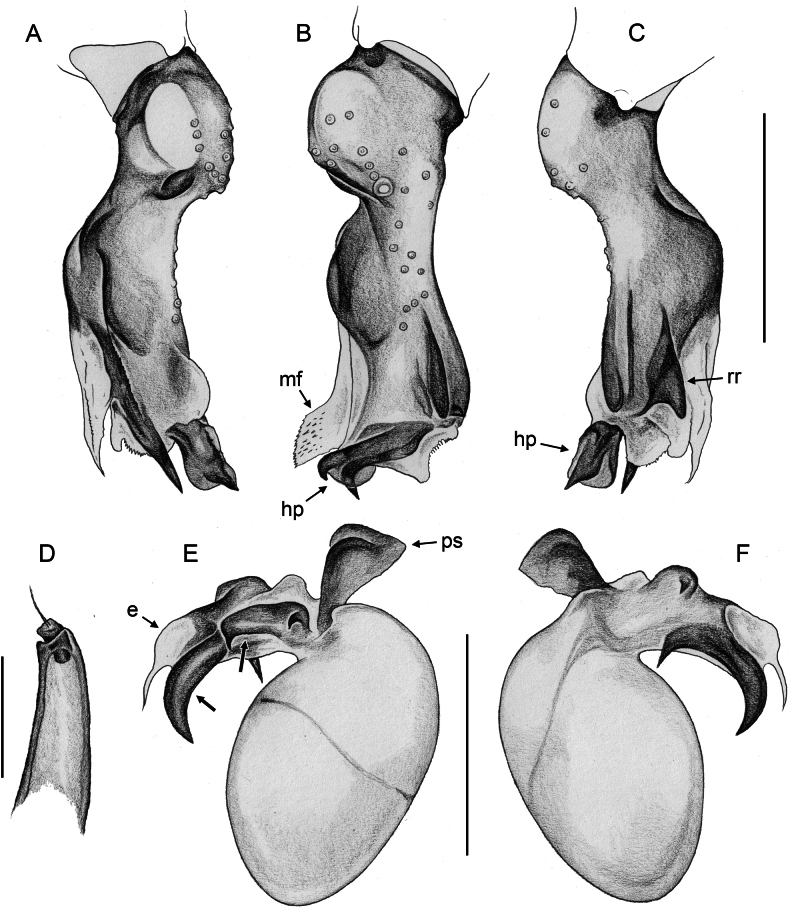
*Micropholcusjacominae* Deeleman-Reinhold & van Harten, 2001; male paratype from Yemen, Al Mahwit, Khamis Bani Sa’d (RMNH) **A–C** left procursus in prolateral, dorsal, and retrolateral views **D** tip of left trochanter apophysis **E, F** left genital bulb in prolateral and retrolateral views; bold arrows in E point at distinctive processes of unclear homology. Abbreviations: e, embolus; hp, dorsal hinged process; mf, membranous prolateral flap; ps, proximal bulbal sclerite; rr, retrolateral ridge; Scale bars: 0.3 mm (**A–C, E, F**); 0.05 mm (**D**).

**Figure 21.**
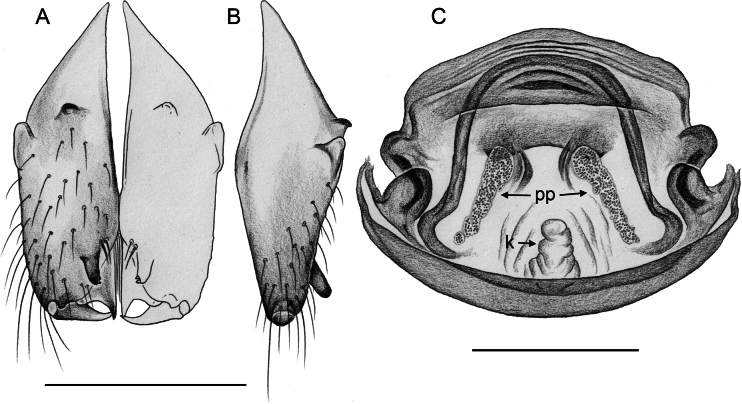
*Micropholcusjacominae* Deeleman-Reinhold & van Harten, 2001; paratypes from Yemen, Al Mahwit, Khamis Bani Sa’d (RMNH) **A, B** male chelicerae, frontal and lateral views **C** cleared female genitalia, dorsal view. Abbreviations: k, epigynal ‘knob’; pp, pore plates. Scale bars: 0.3 mm.

**Figure 22.**
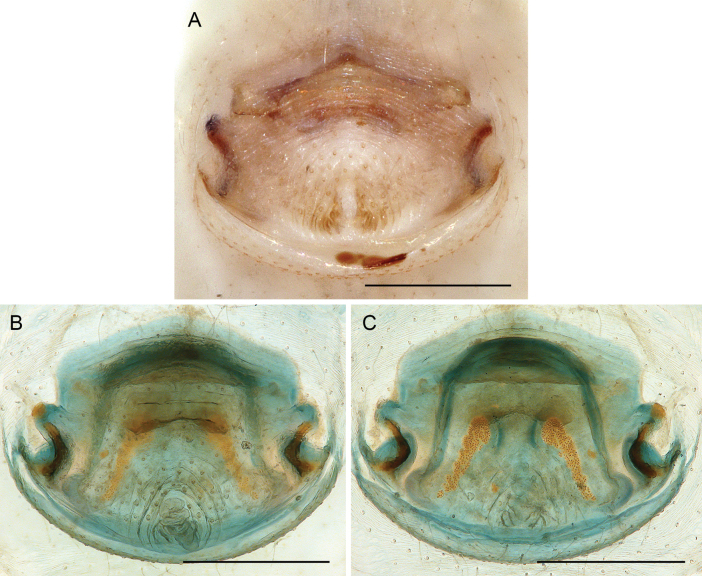
*Micropholcusjacominae* Deeleman-Reinhold & van Harten, 2001; female paratype from Yemen, Al Mahwit, Khamis Bani Sa’d (RMNH) **A** epigynum, ventral view **B, C** cleared female genitalia, ventral and dorsal views. Scale bars: 0.3 mm.

Figs 19 , 20 , 21 , 22

Micropholcus jacominae Deeleman-Reinhold & van Harten, 2001: 199, figs 17, 18, 21–26 (♂♀).

##### Material examined.

Yemen – **Al Mahwit** • 1 ♂, 1 ♀, paratypes; Khamis Bani Sa’d; 15.185°N, 43.510°E (see Note below); 490 m a.s.l.; 11 Oct. 1999; A. van Harten leg.; RMNH ARA 15019.

##### Note.

The coordinates in Deeleman-Reinhold and van Harten (2001) (15°11'N, 43°25'E) mark a spot 10 km W of Khamis Bani Sa’d, at ~ 440 m a.s.l. (rather than 550 m as reported in the original description). We suspect that our coordinates above are closer to the actual collecting site that could only be reconstructed as being “close to Khamis Bani Sa’d, but not in the village” (A. van Harten, pers. comm. 22 Apr. 2021, 13 Mar. 2024).

##### Diagnosis.

Easily distinguished from known congeners by numerous details of male palp: long ventral apophysis on trochanter (Fig. 19C; similar in some Saudi Arabian species, cf. Figs 27C, 31C); distinct dorsal process on femur (arrowed in Fig. 19C; similar only in *M.abha* sp. nov., cf. Fig. 35C); dorsal-distal hinged process on procursus short and directed towards prolateral (Fig. 20A–C); unique processes of genital bulb (Fig. 20E, F), and by female epigynum and internal genitalia (Figs 21C, 22): extensible knob in posterior position; pair of strongly curved lateral internal sclerites; long pore plates widening and only slightly converging anteriorly.

##### Redescription.

**Male. *Measurements*.** Total body length 2.3, carapace width 0.85. Distance PME-PME 200 µm; diameter PME 65 µm; distance PME-ALE 20 µm; distance AME-AME 25 µm; diameter AME 45 µm. Leg 1: 23.3 (5.9 + 0.4 + 5.7 + 10.2 + 1.1), tibia 2: 3.5, tibia 3: 2.1, tibia 4: 2.9; tibia 1 L/d: 76; diameters of leg femora (at half length) 0.08–0.09; of leg tibiae 0.07–0.08.

***Colour*** (in ethanol). Prosoma and legs pale ochre-whitish, carapace with complex brown median mark similar to Saudi Arabian species (cf. Fig. 3); legs with darkened patellae and tibia-metatarsus joints; abdomen pale grey to whitish.

***Body*.** Habitus similar to Saudi Arabian species (cf. Fig. 3). Ocular area raised (distinct in frontal view). Carapace without thoracic groove. Clypeus unmodified. Sternum wider than long (0.50/0.40), unmodified. Abdomen oval, almost twice as long as wide.

***Chelicerae*.** As in Fig. 21A, B; with pair of relatively long distal apophyses near laminae, each with two modified (cone-shaped) hairs; pair of proximal lateral processes weakly sclerotised and directed towards proximal; and pair of small but distinct proximal frontal processes.

***Palps*.** As in Fig. 19; coxa unmodified; trochanter with long ventral apophysis with very indistinct retrolateral hump proximally and modified hair at tip (Fig. 20D); femur with weakly sclerotised but distinct processes retrolateral-dorsally and prolateral-ventrally; femur-patella joints shifted toward prolateral side; tibia-tarsus joints shifted toward retrolateral side; tarsus with large tarsal organ. Procursus (Fig. 20A–C) proximally with sclerotised prolateral hump; at half length with prolateral sclerotised ridge transforming distally into transparent membrane, and brush of dorsal hairs; distally with retrolateral ridge, membranous ventral-prolateral flap, pointed prolateral process, and distinctive dorsal hinged process. Genital bulb (Fig. 20E, F) with strong proximal sclerite; two sclerites of unclear homology, with two pointed processes each; and mostly semi-transparent short embolus, proximally sclerotised, distally membranous with pointed transparent extension.

***Legs*.** Without spines, without curved hairs, without sexually dimorphic short vertical hairs (many hairs missing); retrolateral trichobothrium of tibia 1 at 8%; prolateral trichobothrium absent on tibia 1; tarsus 1 with > 20 pseudosegments, distally distinct.

**Female.** In general very similar to male but ocular area slightly less raised and triads closer together (PME-PME 170 µm), carapace pattern more fragmented than in male. Tibia 1: 4.4. Epigynum (Fig. 22A) anterior plate oval, protruding, with membranous, possibly expandable knob in posterior position, tip directed towards posterior; lateral internal sclerites clearly visible in untreated specimens; posterior epigynal plate very short and indistinct. Internal genitalia (Figs 21C, 22B, C) with pair of long pore plates converging and widening anteriorly, pair of lateral sclerites, and transversal ventral sclerotised band; with sclerotised anterior arc continued to posterior margin.

##### Distribution.

Known from type locality only, in western Yemen (Fig. 13C).

##### Natural history.

Deeleman-Reinhold and van Harten (2001) report that the spiders were shaken from dry plant debris in an irrigated banana plantation. This microhabitat is unusual for *Micropholcus* on the Arabian Peninsula and needs confirmation.

#### ﻿﻿ Micropholcus dhahran

Taxon classificationAnimaliaAraneaePholcidae

Huber sp. nov.

E77B9A4C-0BBE-57AC-923C-A1DBFA32542C

https://zoobank.org/2D3B13EE-4099-47C8-A36F-6DC154FA3742

**Figure 23.**
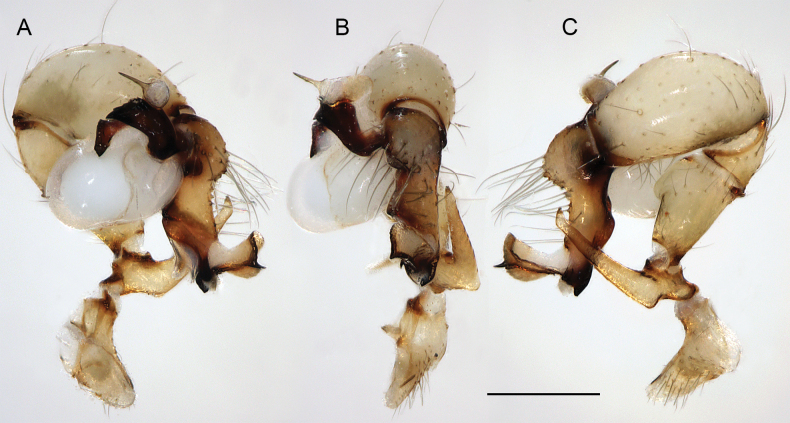
*Micropholcusdhahran* Huber, sp. nov.; male from Saudi Arabia, ‘Asir, W of Dhahran Al Janub (ZFMK Ar 24657). Left palp in prolateral (**A**), dorsal (**B**), and retrolateral (**C**) views. Scale bar: 0.3 mm.

**Figure 24.**
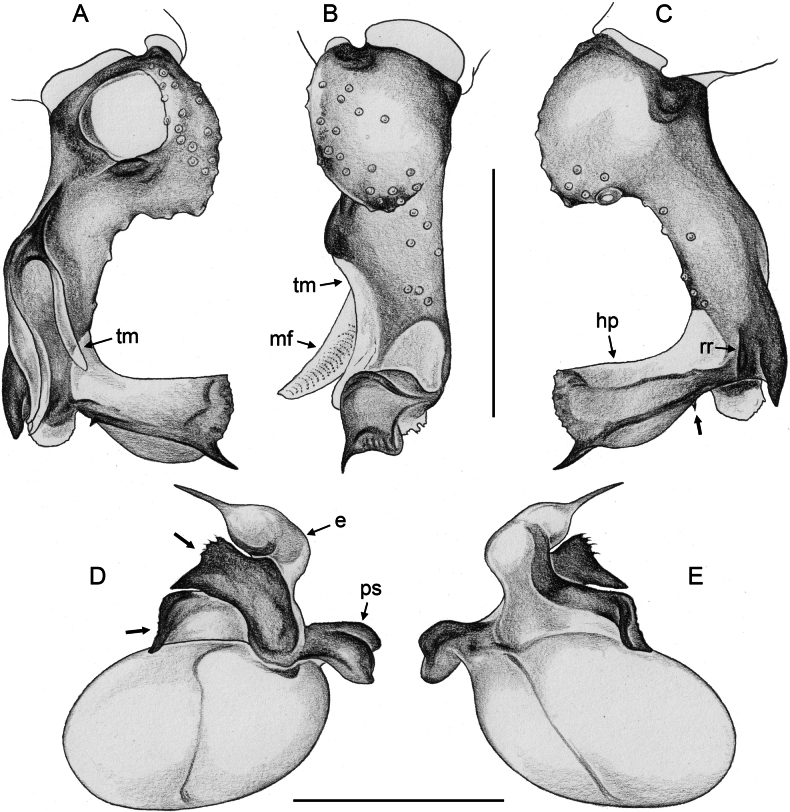
*Micropholcusdhahran* Huber, sp. nov.; male from Saudi Arabia, ‘Asir, W of Dhahran Al Janub (ZFMK Ar 24657) **A–C** left procursus in prolateral, dorsal, and retrolateral views; bold arrow in C points at proximal spine on hinged process **D, E** left genital bulb in prolateral and retrolateral views; bold arrows in D point at distinctive processes of unclear homology. Abbreviations: e, embolus; hp, dorsal hinged process; mf, membranous prolateral flap; ps, proximal bulbal sclerite; rr, retrolateral ridge; tm, transparent membrane; Scale bars: 0.3 mm.

**Figure 25.**
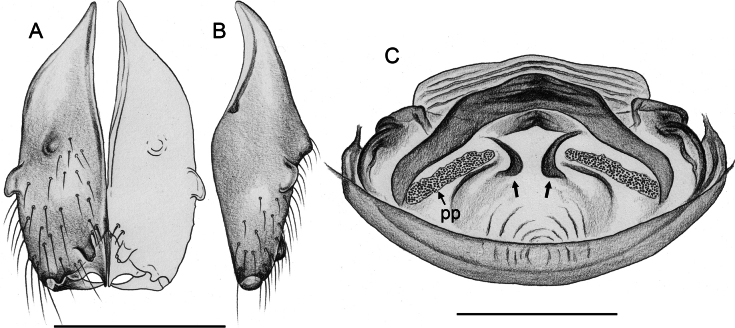
*Micropholcusdhahran* Huber, sp. nov.; from Saudi Arabia, ‘Asir, W of Dhahran Al Janub (ZFMK Ar 24657) **A, B** male chelicerae, frontal and lateral views **C** cleared female genitalia, dorsal view; bold arrows point at crescent-shaped structures. Abbreviation: pp, pore plate. Scale bars: 0.3 mm.

**Figure 26.**
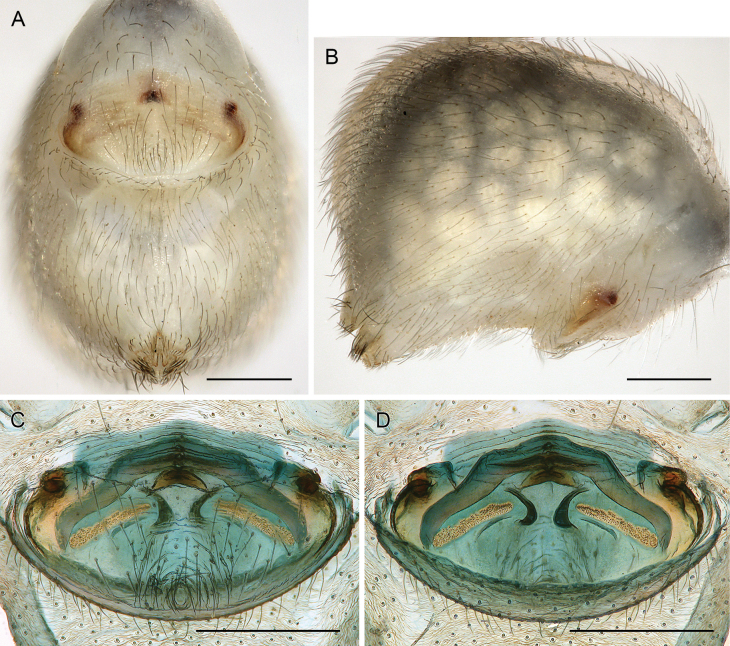
*Micropholcusdhahran* Huber, sp. nov.; female from Saudi Arabia, ‘Asir, W of Dhahran Al Janub (ZFMK Ar 24657) **A, B** abdomen, ventral and lateral views **C, D** cleared genitalia, ventral and dorsal views. Scale bars: 0.3 mm.

Figs 3A , 23 , 24 , 25 , 26

##### Type material.

***Holotype*.** Saudi Arabia – ‘**Asir** • ♂; W of Dhahran Al Janub, ‘site 2'; 17.7010°N, 43.3891°E; 2000 m a.s.l.; 24 Mar. 2024; B.A. Huber leg.; KSMA. ***Paratypes*.** Saudi Arabia – ‘**Asir** • 2 ♂♂, 1 ♀, and 1 cleared ♀ abdomen; same collection data as for holotype; ZFMK Ar 24657.

##### Other material.

Saudi Arabia – ‘**Asir** • 1 ♀ (abdomen cleared and transferred to ZFMK Ar 24657), in pure ethanol; same collection data as for holotype; ZFMK SA121.

##### Diagnosis.

Distinguished from known congeners by unique shapes of bulbal processes (Fig. 24D, E; similar only in *M.abha* sp. nov. but without strong retrolateral spine; without distinctive pointed prolateral sclerite as in geographically closest species, *M.harajah* sp. nov. and *M.alfara* sp. nov.); from most congeners (except *M.harajah* sp. nov. and *M.alfara* sp. nov.) by rectangular dorsal hinged process of procursus with obtuse tip and ventral terminal pointed process and small proximal spine (in other Saudi Arabian species procursus wider and more curved dorsally, without proximal spine; in *M.fauroti* relatively longer and without spine); most congeners (except for three species above and *M.jacominae* and *M.darbat* sp. nov.) also by long trochanter apophysis (Fig. 23C; longer than palpal femur). Female with pair of distinct internal crescent-shaped structures (arrows in Fig. 25C), similar only in *M.harajah* sp. nov. and *M.alfara* sp. nov., but without external pockets as in *M.harajah* sp. nov., crescent-shaped structures farther apart than in *M.alfara* sp. nov., and anterior arc wider and with different curvature than in *M.harajah* sp. nov. and *M.alfara* sp. nov.

##### Description.

**Male** (holotype). ***Measurements*.** Total body length 2.5, carapace width 0.8. Distance PME-PME 180 µm; diameter PME 85 µm; distance PME-ALE 20 µm; distance AME-AME 20 µm; diameter AME 55 µm. Leg 1: 27.1 (6.9 + 0.4 + 6.7 + 12.0 + 1.1), tibia 2: 4.0, tibia 3: 2.1, tibia 4: 3.1; tibia 1 L/d: 84; diameters of leg femora (at half length) 0.09–0.10; of leg tibiae 0.08.

***Colour*** (in ethanol). Carapace pale ochre-yellow with distinct brown mark, ocular area and clypeus without darker pattern; sternum monochromous whitish; legs ochre-yellow, patellae brown, tibia-metatarsus joints with indistinct brown ring, femur 1 proximally barely darkened; abdomen pale ochre-grey, dorsally and laterally with whitish internal marks.

***Body*.** Habitus as in Fig. 3A. Ocular area slightly raised. Carapace without thoracic groove. Clypeus unmodified. Sternum wider than long (0.62/0.50), unmodified. Abdomen oval, approximately twice as long as wide.

***Chelicerae*.** As in Fig. 25A, B; with pair of distal apophyses near laminae, each with two cone-shaped hairs; with pair of low proximal frontal humps; with proximal lateral processes in relatively distal position and directed towards distal.

***Palps*.** As in Fig. 23; coxa unmodified; trochanter with long ventral apophysis with distinct proximal retrolateral hump and modified hair on distal tip; femur distally widened, with distinct ventral hump; femur-patella joints shifted toward prolateral side; tibia-tarsus joints shifted toward retrolateral side; tarsus with large tarsal organ. Procursus (Fig. 24A–C) proximally with sclerotised prolateral hump; at half-length with prolateral sclerotised ridge transforming distally into transparent membrane, and brush of dorsal hairs; distally with small retrolateral ridge and strong ventral apophysis, large bifid membranous ventral-prolateral flap, and distinctive dorsal hinged process. Genital bulb (Fig. 24D, E) with strong proximal sclerite; with two sclerotised processes of unclear homology: prolateral process with dorsal row of teeth with short hair-like processes, retrolateral process originating from basis of embolus, S-shaped and distally slightly spiralling; and mostly semi-transparent short embolus with distinct pointed extension.

***Legs*.** Without spines, without curved hairs, without sexually dimorphic short vertical hairs; retrolateral trichobothrium of tibia 1 at 7%; prolateral trichobothrium absent on tibia 1; tarsus 1 with > 20 pseudosegments, only distally distinct.

***Variation*** (male). Tibia 1 in two other males: 6.5, 6.7.

**Female.** In general very similar to male. Tibia 1 in one female: 4.3 (missing in second female). Epigynum (Fig. 26A, B) barely protruding, anterior plate oval, with indistinct knob-shaped process posteriorly; with pair of lateral and median internal dark structures visible through cuticle; posterior epigynal plate very short and indistinct. Internal genitalia (Figs 25C, 26C, D) with pair of elongated pore plates in transversal position; with pair of lateral sclerites, median crescent-shaped structures, and large membranous anterior arc.

##### Etymology.

The species name is derived from the type locality; noun in apposition.

##### Distribution.

Known from type locality only, in Saudi Arabia, ‘Asir Province (Fig. 13C).

##### Natural history.

The spiders were found sitting on the undersides of large boulders, in small cave-like spaces between ground and boulder.

#### ﻿﻿ Micropholcus harajah

Taxon classificationAnimaliaAraneaePholcidae

Huber sp. nov.

291E5312-3546-5CCA-9EBF-FA4254C7AF0F

https://zoobank.org/249E0B68-DBB7-4601-81CF-FD7CCFD414AF

**Figure 27.**
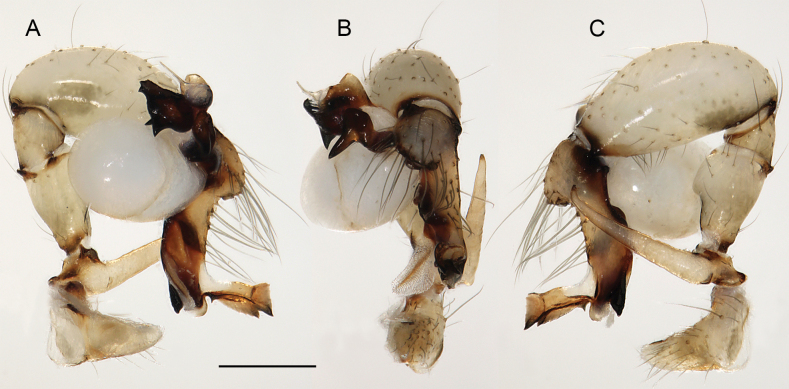
*Micropholcusharajah* Huber, sp. nov.; male from Saudi Arabia, ‘Asir, SE of Harajah (ZFMK Ar 24658). Left palp in prolateral (**A**), dorsal (**B**), and retrolateral (**C**) views. Scale bar: 0.3 mm.

**Figure 28.**
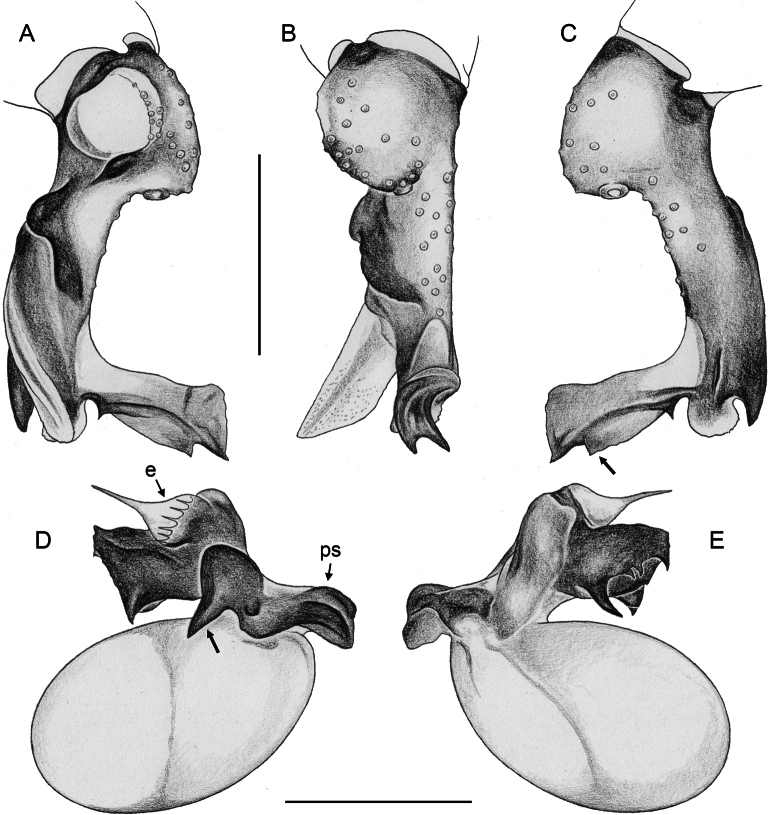
*Micropholcusharajah* Huber, sp. nov.; male from Saudi Arabia, ‘Asir, SE of Harajah (ZFMK Ar 24658) **A–C** left procursus in prolateral, dorsal, and retrolateral views; bold arrowed in C points at distinctive subdistal projection on hinged process **D, E** left genital bulb in prolateral and retrolateral views; bold arrowed in D points at distinctive cone on prolateral bulbal process. Abbreviations: e, embolus; ps, proximal bulbal sclerite. Scale bars: 0.3 mm.

**Figure 29.**
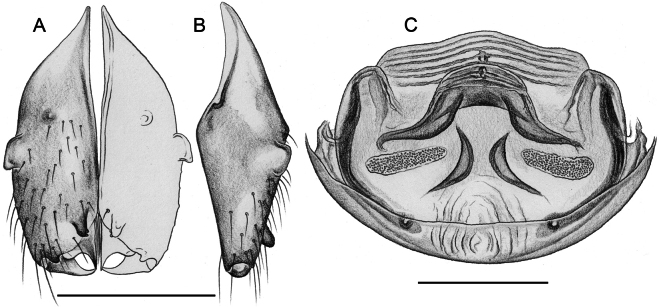
*Micropholcusharajah* Huber, sp. nov.; from Saudi Arabia, ‘Asir, SE of Harajah **A, B** male chelicerae, frontal and lateral views (ZFMK Ar 24658) **C** cleared female genitalia, dorsal view (ZFMK Ar 24659). Scale bars: 0.3 mm.

**Figure 30.**
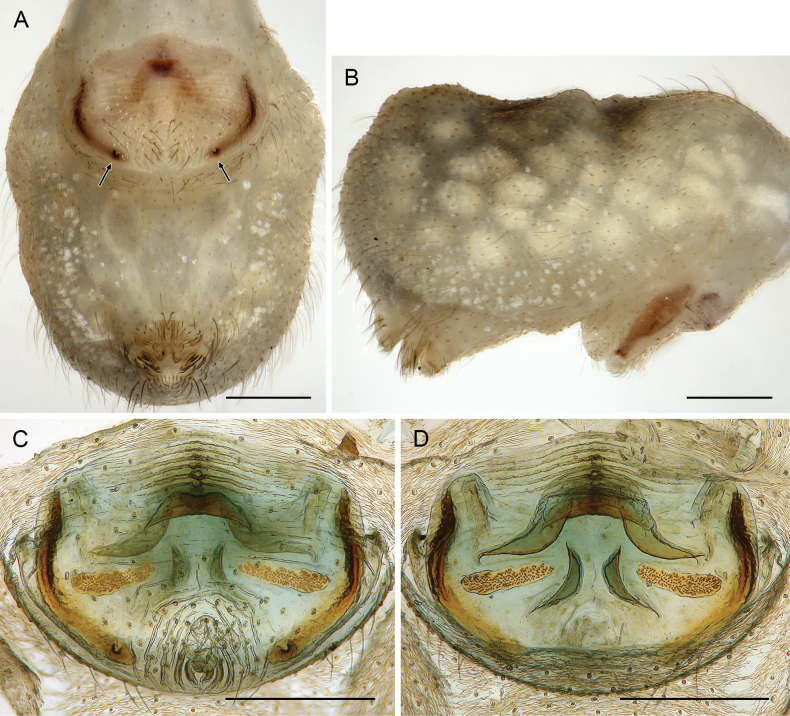
*Micropholcusharajah* Huber, sp. nov.; female from Saudi Arabia, ‘Asir, SE of Harajah (ZFMK Ar 24659) **A, B** abdomen, ventral and lateral views; arrows in A point at unique epigynal pockets **C, D** cleared genitalia, ventral and dorsal views. Scale bars: 0.3 mm.

Figs 3B , 27 , 28 , 29 , 30

##### Type material.

***Holotype*.** Saudi Arabia – ‘**Asir** • ♂; SE of Al Harajah, ‘site 1’; 17.8681°N, 43.3943°E; 2370 m a.s.l.; 22 Mar. 2024; B.A. Huber leg.; KSMA. ***Paratypes*.** Saudi Arabia – ‘**Asir** • 2 ♂♂, 7 ♀♀; same collection data as for holotype; ZFMK Ar 24658 and 24659.

##### Other material.

Saudi Arabia – ‘**Asir** • 3 ♀♀, in pure ethanol; same collection data as for holotype; ZFMK SA114.

##### Diagnosis.

Distinguished from known congeners by unique shapes of bulbal processes, in particular distinctive prolateral sclerite (arrowed in Fig. 28D; similar only in *M.alfara* sp. nov.) and by unique subdistal conical projection on hinged process of procursus (arrowed in Fig. 28C); from most congeners (except *M.alfara* sp. nov. and *M.dhahran* sp. nov.) also by rectangular hinged process of procursus with obtuse tip and small ventral terminal pointed process and small proximal spine (in other Saudi Arabian species procursus wider and more curved dorsally, without proximal spine; in *M.fauroti* relatively longer and without spine); from most congeners (except for three species above and *M.jacominae* and *M.darbat* sp. nov.) also by long trochanter apophysis (Fig. 27C; longer than palpal femur). Female with unique pair of external epigynal pockets (arrowed in Fig. 30A); with distinct internal crescent shaped structures (Fig. 29C; similar only in *M.dhahran* sp. nov. and *M.alfara* sp. nov.), crescent-shaped structures farther apart than in *M.alfara* sp. nov., and anterior arc narrower and with different curvature than in *M.dhahran* sp. nov.

##### Description.

**Male** (holotype). ***Measurements*.** Total body length 2.7, carapace width 0.9. Distance PME-PME 200 µm; diameter PME 80 µm; distance PME-ALE 20 µm; distance AME-AME 25 µm; diameter AME 55 µm. Leg 1: 26.2 (6.5 + 0.5 + 6.4 + 11.6 + 1.2), tibia 2: 4.0, tibia 3: 2.3, tibia 4: 3.3; tibia 1 L/d: 75; diameters of leg femora (at half length) 0.10–0.11; of leg tibiae 0.085.

***Colour*** (in ethanol). Carapace pale ochre-yellow with large brown median mark connected posteriorly to series of small lateral marks, ocular area slightly darkened, clypeus without darker pattern; sternum mostly whitish, posteriorly slightly darkened; legs ochre-yellow, patellae brown, tibia-metatarsus joints with indistinct brown ring, femur 1 proximally slightly darkened; abdomen pale ochre-grey, dorsally and laterally with larger whitish internal marks.

***Body*.** Habitus as in *M.dhahran* sp. nov. (cf. Fig. 3A). Ocular area slightly raised (more distinct in frontal view). Carapace without thoracic groove. Clypeus unmodified. Sternum wider than long (0.66/0.50), unmodified. Abdomen oval, approximately twice as long as wide.

***Chelicerae*.** As in Fig. 29A, B; with pair of distal apophyses near laminae, each with two cone-shaped hairs; with pair of very low proximal frontal humps; with prominent pair of proximal lateral processes.

***Palps*.** As in Fig. 27; coxa unmodified; trochanter with very long ventral apophysis with small proximal retrolateral hump and modified hair on distal tip; femur small relative to tibia, distally widened, with distinct ventral hump; femur-patella joints shifted toward prolateral side; tibia-tarsus joints shifted toward retrolateral side; tarsus with large tarsal organ. Procursus (Fig. 28A–C) proximally with sclerotised prolateral hump; at half-length with prolateral-ventral sclerotised ridge, prolateral thick sclerotised ridge, and brush of dorsal hairs; distally with small retrolateral ridge and strong ventral apophysis, large membranous ventral-prolateral flap, and distinctive dorsal hinged process. Genital bulb (Fig. 28D, E) with strong proximal sclerite; two sclerotised processes of unclear homology: prolateral process with distinctive strong pointed cone directed towards bulbous part of genital bulb; retrolateral process originating from basis of embolus, heavily sclerotised with retrolateral row of four variably strong pointed processes; and mostly semi-transparent short embolus with distinct pointed process and subdistal row of transparent hair-like processes prolaterally.

***Legs*.** Without spines, without curved hairs, without sexually dimorphic short vertical hairs; retrolateral trichobothrium of tibia 1 not seen in holotype, in paratype at 7%; prolateral trichobothrium absent on tibia 1; tarsus 1 with > 25 pseudosegments, distally distinct.

***Variation*** (male). Tibia 1 in other male: 5.9; missing in third male.

**Female.** In general very similar to male. Tibia 1 in ten females: 4.3–5.9 (mean 4.9). Epigynum (Fig. 30A, B) variably protruding, anterior plate oval, with indistinct knob-shaped process posteriorly; with pair of lateral sclerites, each provided at posterior end with small pocket, and median internal dark structure visible through cuticle; posterior epigynal plate very short and indistinct. Internal genitalia (Figs 29C, 30C, D) with pair of elongated pore plates in transversal position; with pair of lateral sclerites, median crescent-shaped structures, and large membranous anterior arc.

##### Etymology.

The species name is derived from the type locality; noun in apposition.

##### Distribution.

Known from type locality only, in Saudi Arabia, ‘Asir Province (Fig. 13C).

##### Natural history.

The spiders were found sitting on the undersides of large boulders (Fig. 14A), in small cave-like spaces between boulders and between the ground and boulder. Two egg sacs contained ~ 30–35 eggs each, with an egg diameter of 0.58–0.60 mm.

#### ﻿﻿ Micropholcus alfara

Taxon classificationAnimaliaAraneaePholcidae

Huber sp. nov.

F4469F1D-2FD8-58FD-A939-9F2B79665886

https://zoobank.org/EC6070DD-193E-427D-B5A6-1354760EE20C

Figs 3C, D , 6A, B , 7A, B , 9A , 11D, E , 31 , 32 , 33 , 34

##### Type material.

***Holotype*.** Saudi Arabia – ‘**Asir** • ♂; S of Al Fara; 18.0487°N, 42.7096°E; 2250 m a.s.l.; 21 Mar. 2024; B.A. Huber leg.; KSMA. ***Paratypes*.** Saudi Arabia – ‘**Asir** • 4 ♂♂, 5 ♀♀ (1 ♂, 1 ♀ used for SEM); same collection data as for holotype; ZFMK Ar 24660 to 24661.

##### Other material.

Saudi Arabia – ‘**Asir** • 1 ♂, 1 ♀, in pure ethanol; same collection data as for holotype; ZFMK SA112.

##### Diagnosis.

**Figure 31.**
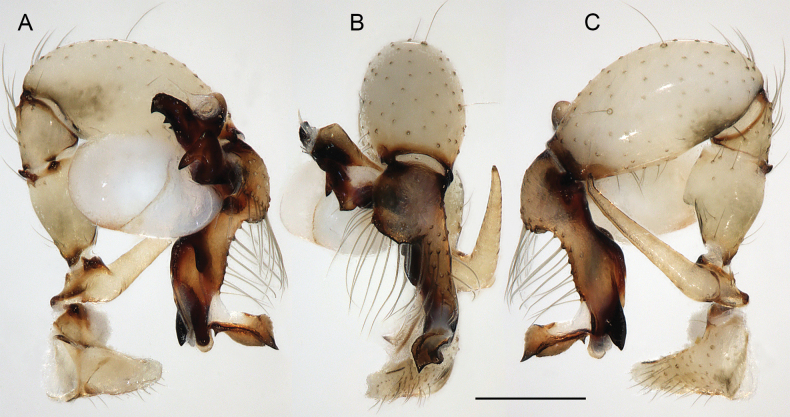
*Micropholcusalfara* Huber, sp. nov.; male from Saudi Arabia, ‘Asir, S of Al Fara (ZFMK Ar 24660). Left palp in prolateral (**A**), dorsal (**B**), and retrolateral (**C**) views. Scale bar: 0.3 mm.

**Figure 32.**
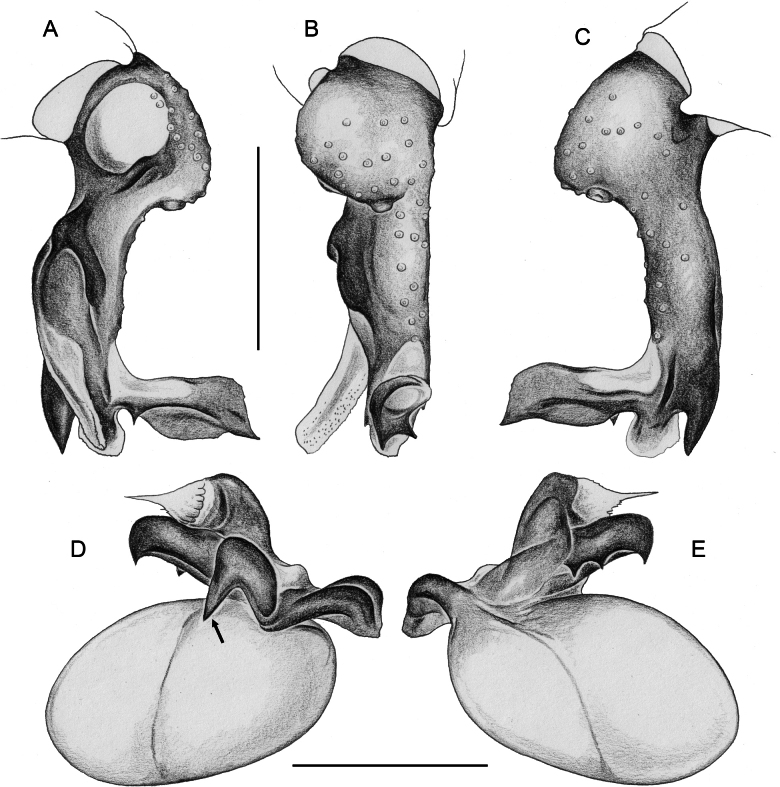
*Micropholcusalfara* Huber, sp. nov.; male from Saudi Arabia, ‘Asir, S of Al Fara (ZFMK Ar 24660) **A–C** left procursus in prolateral, dorsal, and retrolateral views **D, E** left genital bulb in prolateral and retrolateral views; arrow in D points at distinctive cone on prolateral bulbal process. Scale bars: 0.3 mm.

**Figure 33.**
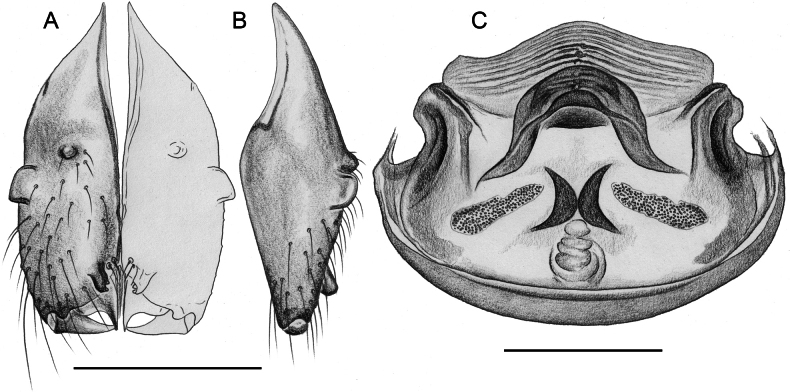
*Micropholcusalfara* Huber, sp. nov.; from Saudi Arabia, ‘Asir, S of Al Fara **A, B** male chelicerae, frontal and lateral views (ZFMK Ar 24660) **C** cleared female genitalia, dorsal view (ZFMK Ar 24661). Scale bars: 0.3 mm.

Distinguished from known congeners by unique shapes of bulbal processes, in particular distinctive prolateral sclerite (arrowed in Fig. 32D; similar only in *M.harajah* sp. nov., cf. Fig. 28D); from most congeners (except *M.harajah* sp. nov. and *M.dhahran* sp. nov.) also by rectangular hinged process of procursus with obtuse tip and small ventral terminal pointed process and small proximal spine (in other Saudi Arabian species procursus wider and more curved dorsally, without proximal spine; in *M.fauroti* relatively longer and without spine); from most congeners (except for three species above and *M.jacominae* and *M.darbat* sp. nov.) also by long trochanter apophysis (Fig. 31C; longer than palpal femur). Female with distinct internal crescent-shaped structures (Fig. 33C), similar only in *M.dhahran* sp. nov. and *M.harajah* sp. nov., but closer together; anterior arc narrow (Fig. 33C), similar only in *M.harajah* sp. nov. (cf. Fig. 29C), in *M.dhahran* sp. nov. wider and with different curvature. From *M.harajah* sp. nov. also distinguished by absence of subdistal conical projection on hinged process of procursus (cf. Fig. 28C) and by absence of pair of external epigynal pockets (cf. Fig. 30A).

##### Description.

**Male** (holotype). ***Measurements*.** Total body length 2.6, carapace width 1.0. Distance PME-PME 185 µm; diameter PME 80 µm; distance PME-ALE 25 µm; distance AME-AME 20 µm; diameter AME 55 µm. Leg 1: 28.4 (7.1 + 0.4 + 6.9 + 12.8 + 1.2), tibia 2: 4.1, tibia 3: 2.3, tibia 4: 3.3; tibia 1 L/d: 81; diameters of leg femora (at half length) 0.09–0.10; of leg tibiae 0.085.

***Colour*** (in ethanol). Carapace pale ochre-yellow with large brown median mark connected posteriorly to series of small lateral marks, ocular area slightly darkened, clypeus without darker pattern; sternum whitish; legs ochre-yellow, patellae brown, tibia-metatarsus joints with indistinct brown ring, femur 1 proximally slightly darkened (very indistinct); abdomen ochre-grey, dorsally and laterally with large whitish internal marks.

***Body*.** Habitus as in Fig. 3C. Ocular area slightly raised (more distinct in frontal view). Carapace without thoracic groove. Clypeus unmodified. Sternum wider than long (0.64/0.48), unmodified. Abdomen oval, approximately twice as long as wide.

***Chelicerae*.** As in Fig. 33A, B; with pair of distal apophyses near laminae, each with two cone-shaped hairs (Fig. 6A, B); with pair of proximal frontal processes; with prominent pair of proximal lateral processes.

***Palps*.** As in Fig. 31; coxa unmodified; trochanter with very long ventral apophysis with small proximal retrolateral hump and modified hair on distal tip (Fig. 9A); femur small relative to tibia, distally widened, with distinct ventral hump; femur-patella joints shifted toward prolateral side; tibia-tarsus joints shifted toward retrolateral side; tarsus with large tarsal organ. Procursus (Fig. 32A–C) proximally with sclerotised prolateral hump; at half-length with prolateral-ventral sclerotised ridge, prolateral thick sclerotised ridge, and brush of dorsal hairs; distally with small retrolateral ridge and strong ventral apophysis, membranous ventral-prolateral flap (Fig. 7A), and dorsal hinged process. Genital bulb (Figs 7B, 32D, E) with strong proximal sclerite; with two sclerotised processes of unclear homology: prolateral process with strong pointed cone directed towards bulbous part of genital bulb; retrolateral process originating from basis of embolus, heavily sclerotised with retrolateral row of pointed cones of similar sizes; and mostly semi-transparent short embolus with membranous pointed extension and subdistal row of transparent hair-like processes prolaterally.

***Legs*.** Without spines, without curved hairs, without sexually dimorphic short vertical hairs; retrolateral trichobothrium of tibia 1 at 6%; prolateral trichobothrium absent on tibia 1; tarsus 1 with > 20 pseudosegments, distally distinct.

***Variation*** (male). Tibia 1 in five males: 6.8–7.2 (mean 7.0).

**Figure 34.**
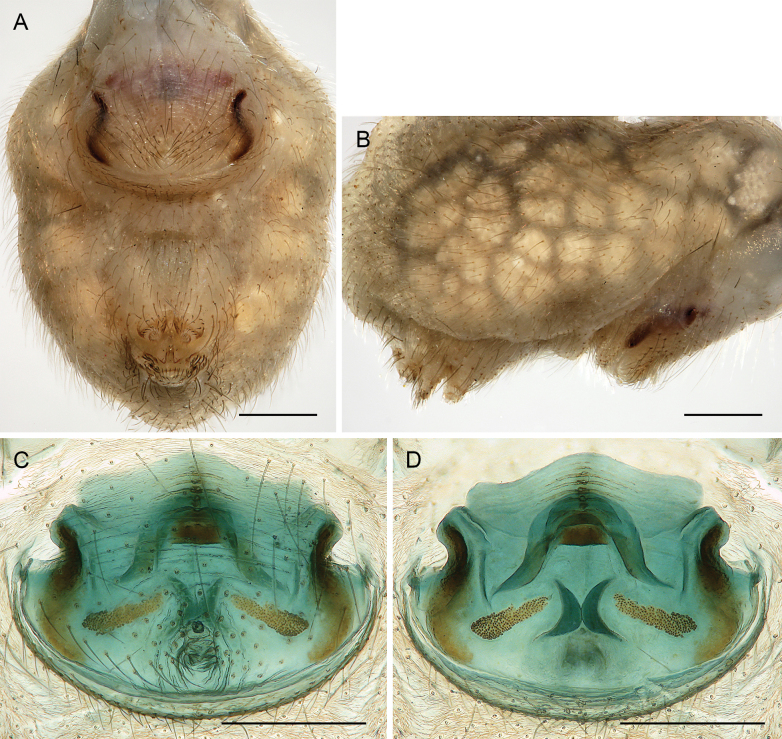
*Micropholcusalfara* Huber, sp. nov.; female from Saudi Arabia, ‘Asir, S of Al Fara (ZFMK Ar 24661) **A, B** abdomen, ventral and lateral views **C, D** cleared genitalia, ventral and dorsal views. Scale bars: 0.3 mm.

**Female.** In general very similar to male. Tibia 1 in five females: 4.6–5.4 (mean 5.0). Epigynum (Fig. 34A, B) variably protruding, anterior plate oval, with small and indistinct knob-shaped process posteriorly; with pair of lateral sclerites bent towards lateral anteriorly, without small pockets at posterior ends; and median internal dark structure visible through cuticle. Posterior epigynal plate very short and indistinct. Internal genitalia (Figs 33C, 34C, D) with pair of elongated pore plates in transversal position; with pair of lateral sclerites, median crescent-shaped structures, and large membranous anterior arc.

##### Etymology.

The species name is derived from the type locality; noun in apposition.

##### Distribution.

Known from type locality only, in Saudi Arabia, ‘Asir Province (Fig. 13C).

##### Natural history.

The spiders were found sitting on the undersides of large boulders (Fig. 14B), in small cave-like spaces between ground and boulder. Two egg sacs had diameters of 2.0–2.2 mm, and contained ~ 25–35 eggs each, with an egg diameter of 0.58–0.62 mm.

#### ﻿﻿ Micropholcus abha

Taxon classificationAnimaliaAraneaePholcidae

Huber sp. nov.

53FDD439-B686-552A-BF6B-6D664E5D6143

https://zoobank.org/60AE7E1A-D17E-48E5-9318-462D97404995

**Figure 35.**
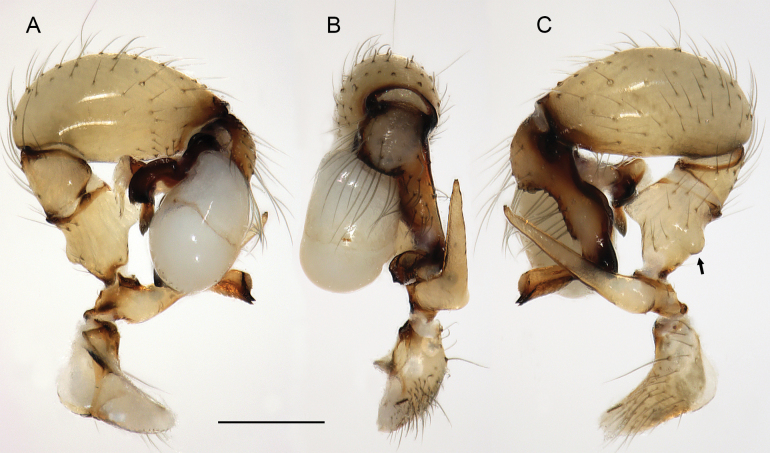
*Micropholcusabha* Huber, sp. nov.; male from Saudi Arabia, ‘Asir, N of Abha (ZFMK Ar 24662). Left palp in prolateral (**A**), dorsal (**B**), and retrolateral (**C**) views; arrow in C points at distinctive dorsal process on femur. Scale bar: 0.3 mm.

**Figure 36.**
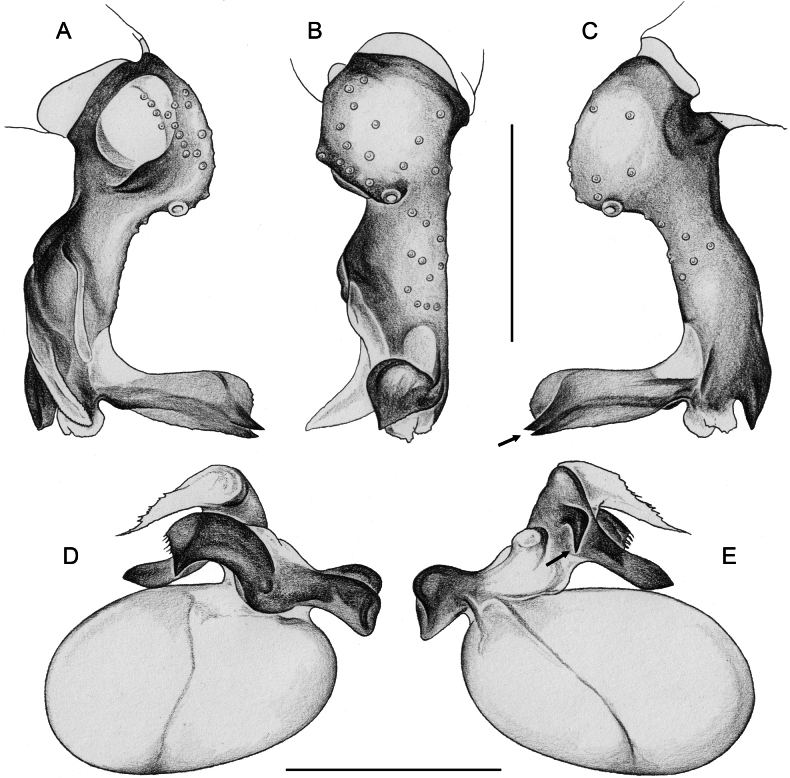
*Micropholcusabha* Huber, sp. nov.; male from Saudi Arabia, ‘Asir, N of Abha (ZFMK Ar 24662) **A–C** left procursus in prolateral, dorsal, and retrolateral views; arrow in C pointsat distinctive bipartite tip of hinged process **D, E** left genital bulb in prolateral and retrolateral views; arrow in E points at distinctive retrolateral pointed process. Scale bars: 0.3 mm.

**Figure 37.**
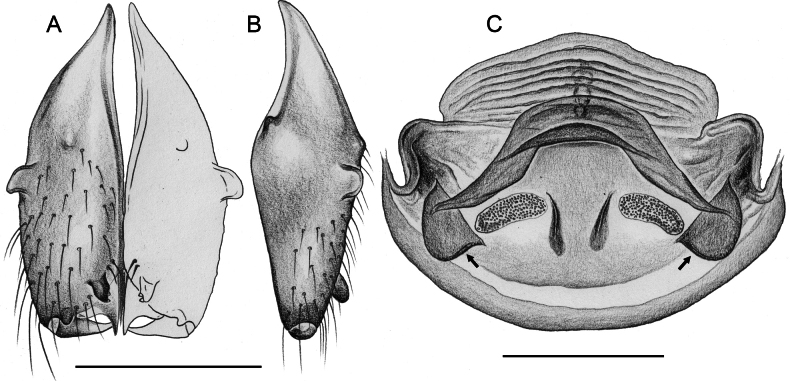
*Micropholcusabha* Huber, sp. nov.; from Saudi Arabia, ‘Asir, N of Abha (ZFMK Ar 24662) **A, B** male chelicerae, frontal and lateral views **C** cleared female genitalia, dorsal view; arrows point at distinctive lateral sacs. Scale bars: 0.3 mm.

**Figure 38.**
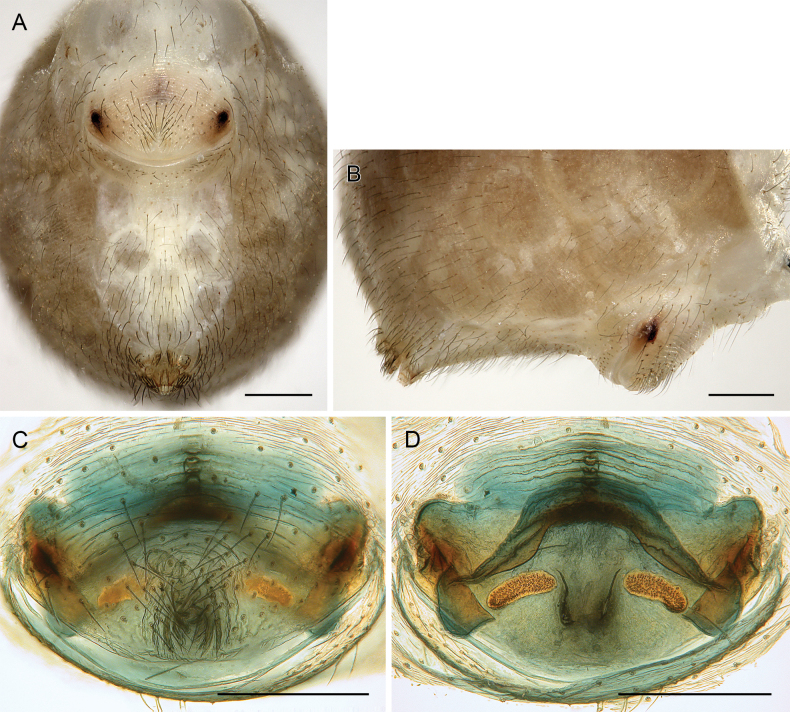
*Micropholcusabha* Huber, sp. nov.; female from Saudi Arabia, ‘Asir, N of Abha (ZFMK Ar 24662) **A, B** abdomen, ventral and lateral views **C, D** cleared genitalia, ventral and dorsal views. Scale bars: 0.3 mm.

Figs 3E , 35 , 36 , 37 , 38

##### Type material.

***Holotype*.** Saudi Arabia – ‘**Asir** • ♂; N of Abha; 18.4168°N, 42.4646°E; 2160 m a.s.l.; 21 Mar. 2024; B.A. Huber leg.; KSMA. ***Paratypes*.** Saudi Arabia – ‘**Asir** • 4 ♂♂, 1 ♀, and 1 cleared ♀ abdomen; same collection data as for holotype; ZFMK Ar 24662.

##### Other material.

Saudi Arabia – ‘**Asir** • 1 ♂, 3 ♀♀ (one abdomen transferred to ZFMK Ar 24662), in pure ethanol; same collection data as for holotype; ZFMK SA111.

##### Diagnosis.

Distinguished from known congeners by presence of dorsal process on palpal femur (arrowed in Fig. 35C; similar only in *M.jacominae*), by bipartite tip of dorsal hinged process of procursus (arrowed in Fig. 36C), and by unique shapes of bulbal processes (Fig. 36D, E; similar to *M.dhahran* sp. nov. but with unique retrolateral pointed process); from similar congeners of southern Saudi Arabia (*M.abha* sp. nov., *M.harajah* sp. nov., *M.dhahran* sp. nov.) also by hinged process of procursus without small proximal spine; from most congeners (except for three species above and *M.jacominae* and *M.darbat* sp. nov.) also by long trochanter apophysis (Fig. 35C; longer than palpal femur). Female with unique lateral sacs in internal genitalia (arrows in Fig. 37C); with distinct anterior arc as in similar congeners from southern Saudi Arabia (*M.alfara* sp. nov., *M.harajah* sp. nov., *M.dhahran* sp. nov.) but without distinct internal crescent-shaped structures (possible homologues visible between pore plates). From *M.harajah* sp. nov. also distinguished by absence of subdistal conical projection on hinged process of procursus (cf. Fig. 28C) and by absence of pair of external epigynal pockets (cf. Fig. 30A).

##### Description.

**Male** (holotype). ***Measurements*.** Total body length 2.4, carapace width 0.9. Distance PME-PME 195 µm; diameter PME 70 µm; distance PME-ALE 20 µm; distance AME-AME 25 µm; diameter AME 50 µm. Leg 1: 27.1 (6.8 + 0.4 + 6.8 + 11.9 + 1.2), tibia 2: 4.1, tibia 3: 2.5, tibia 4: 3.5; tibia 1 L/d: 85; diameters of leg femora (at half length) 0.085–0.095; of leg tibiae 0.080.

***Colour*** (in ethanol). Carapace pale ochre-yellow with large brown median mark divided medially, ocular area not darkened, clypeus slightly darkened; sternum monochromous whitish; legs pale ochre-yellow, patellae and tibia-metatarsus joints barely darkened, femur 1 proximally barely darkened; abdomen pale ochre-grey, dorsally and laterally with large whitish internal marks.

***Body*.** Habitus as in Fig. 3E. Ocular area slightly raised (distinct in frontal view). Carapace without thoracic groove. Clypeus unmodified. Sternum wider than long (0.66/0.54), unmodified. Abdomen oval, approximately twice as long as wide.

***Chelicerae*.** As in Fig. 37A, B; with pair of distal apophyses near laminae, each with two cone-shaped hairs; with pair of very low proximal frontal humps; with prominent pair of proximal lateral processes.

***Palps*.** As in Fig. 35; coxa unmodified; trochanter with very long ventral apophysis with distinct proximal bend and modified hair on distal tip; femur small relative to tibia, distally widened, with distinct ventral and dorsal humps; femur-patella joints shifted toward prolateral side; tibia-tarsus joints shifted toward retrolateral side; tarsus with large tarsal organ. Procursus (Fig. 36A–C) proximally with sclerotised prolateral hump; at half-length with prolateral-ventral sclerotised ridge transforming prolaterally into transparent membrane, and brush of dorsal hairs; distally with small retrolateral ridge and strong ventral apophysis, large membranous ventral-prolateral flap, and distinctive dorsal hinged process. Genital bulb (Fig. 36D, E) with strong proximal sclerite; with two sclerotised processes of unclear homology: prolateral process with strong pointed cone directed towards bulbous part of genital bulb and with some hair-like extensions; retrolateral process originating from basis of embolus, heavily sclerotised with strong retrolateral pointed process and flattened distal apophysis; and mostly semi-transparent short embolus with fringed membranous tip.

***Legs*.** Without spines, without curved hairs, without sexually dimorphic short vertical hairs; retrolateral trichobothrium of tibia 1 at 6%; prolateral trichobothrium absent on tibia 1; tarsus 1 with > 20 pseudosegments, distally distinct.

***Variation*** (male). Tibia 1 in five males (incl. holotype): 5.9–6.8 (mean 6.3); clypeus in other males not or barely darkened.

**Female.** In general very similar to male; sternum margins slightly darkened. Tibia 1 in two females: 4.7, 5.2 (missing in other females). Epigynum (Fig. 38A, B) slightly protruding, anterior plate oval, with long but transparent and indistinct knob-shaped process posteriorly; with pair of dark brown lateral sclerites, and median internal dark structure poorly visible through cuticle; posterior epigynal plate very short and indistinct. Internal genitalia (Figs 37C, 38C, D) with pair of elongated pore plates in transversal position; with pair of lateral sclerites, pair of large distinctive lateral sacs, median ridges, and large membranous anterior arc.

##### Etymology.

The species name is derived from the type locality; noun in apposition.

##### Distribution.

Known from type locality only, in Saudi Arabia, ‘Asir Province (Fig. 13C).

##### Natural history.

The spiders were found in small caverns at rock outcrops in an open environment (Fig. 14C), i.e., in less sheltered microhabitats than most other species.

#### ﻿﻿ Micropholcus tanomah

Taxon classificationAnimaliaAraneaePholcidae

Huber sp. nov.

933148E9-7DF3-57DC-8194-F5ED2DFB17B1

https://zoobank.org/6D679588-8300-427F-89A7-6CA93102C27C

Figs 3F , 5A, G , 6C , 7C–G , 9B, F, G , 10A, B , 11A, F , 12A–D , 39 , 40 , 41 , 42

##### Type material.

***Holotype*.** Saudi Arabia – ‘**Asir** • ♂; NW of Tanomah; 19.0220°N, 42.1247°E; 2250 m a.s.l.; 19 Mar. 2024; B.A. Huber leg.; KSMA. ***Paratypes*.** Saudi Arabia – ‘**Asir** • 21 ♂♂, 24 ♀♀, 1 juv. (1 ♂, 1 ♀ used for SEM); same collection data as for holotype; ZFMK Ar 24663 to 24664.

##### Other material.

Saudi Arabia – ‘**Asir** • 3 ♀♀, 4 juvs; in pure ethanol; same collection data as for holotype; ZFMK SA100.

##### Diagnosis.

**Figure 39.**
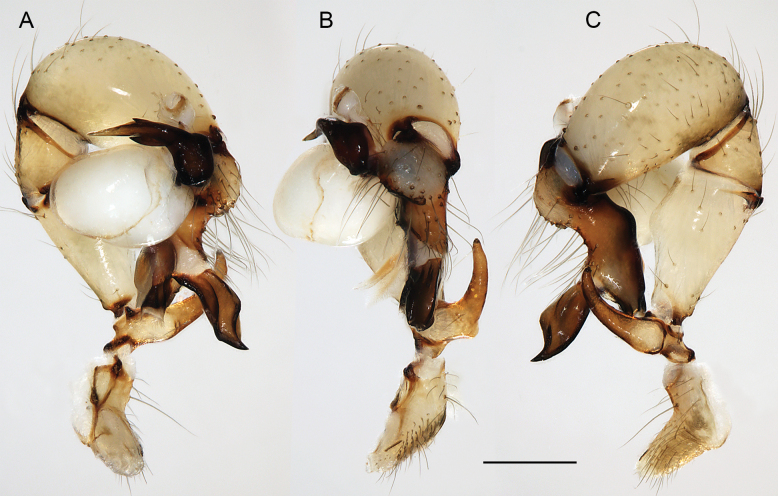
*Micropholcustanomah* Huber, sp. nov.; male from Saudi Arabia, ‘Asir, NW of Tanomah (ZFMK Ar 24663). Left palp in prolateral (**A**), dorsal (**B**), and retrolateral (**C**) views. Scale bar: 0.3 mm.

**Figure 40.**
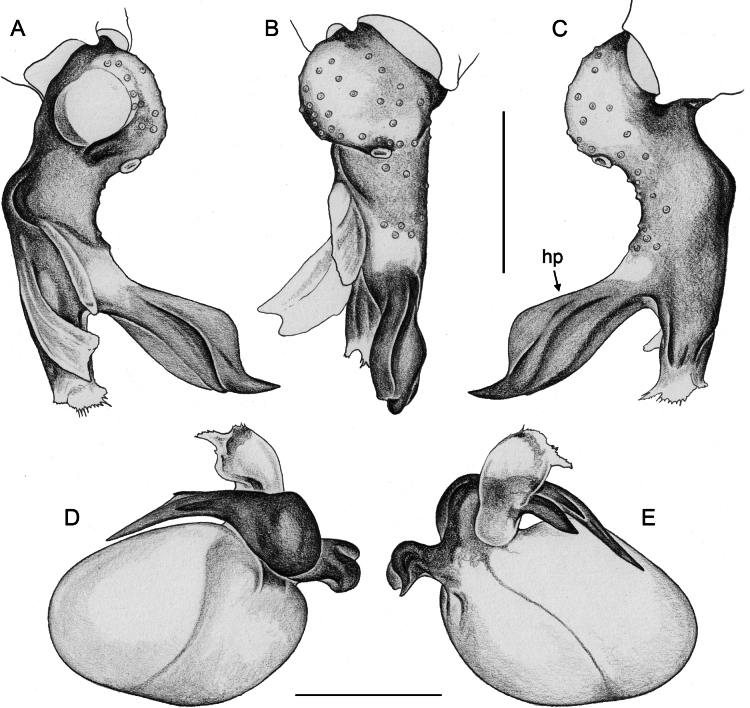
*Micropholcustanomah* Huber, sp. nov.; male from Saudi Arabia, ‘Asir, NW of Tanomah (ZFMK Ar 24663) **A–C** left procursus in prolateral, dorsal, and retrolateral views **D, E** left genital bulb in prolateral and retrolateral views. Abbreviation: hp, dorsal hinged process. Scale bars: 0.3 mm.

**Figure 41.**
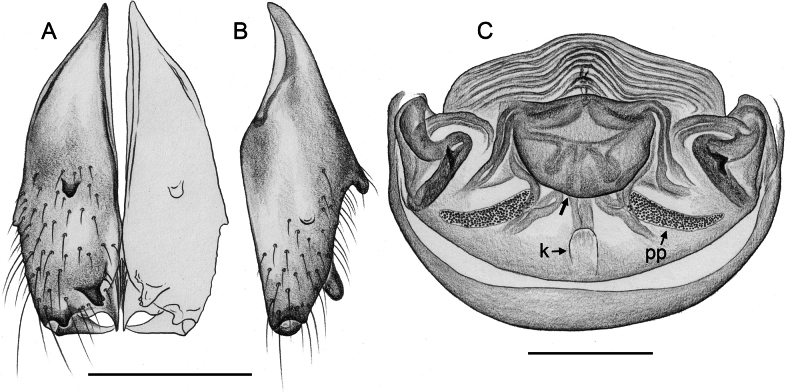
*Micropholcustanomah* Huber, sp. nov.; male from Saudi Arabia, ‘Asir, NW of Tanomah **A, B** male chelicerae, frontal and lateral views (ZFMK Ar 24663) **C** cleared female genitalia, dorsal view (ZFMK Ar 24664); bold arrow points at membranous central element. Abbreviations: k, epigynal ‘knob’; pp, pore plate. Scale bars: 0.3 mm.

**Figure 42.**
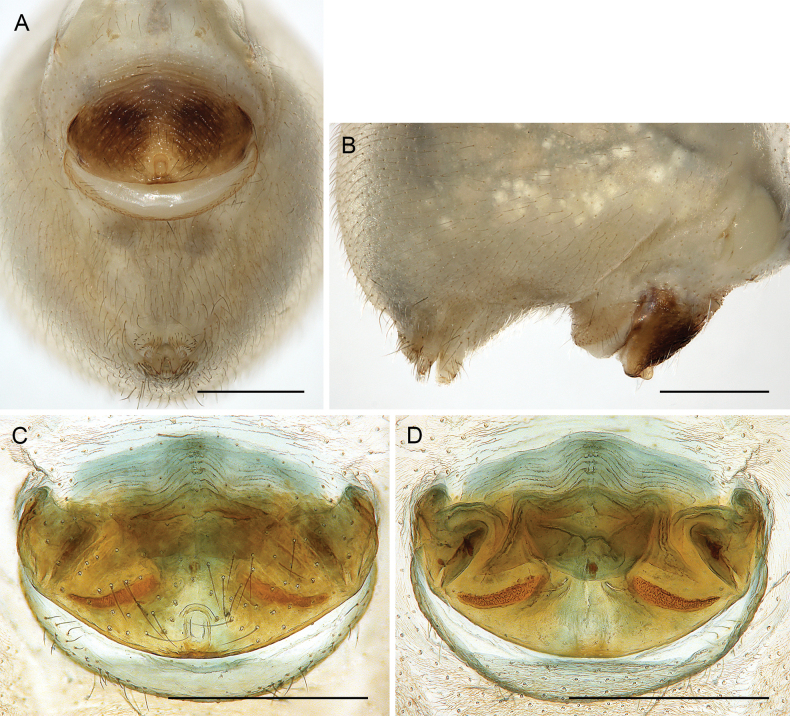
*Micropholcustanomah* Huber, sp. nov.; female from Saudi Arabia, ‘Asir, NW of Tanomah (ZFMK Ar 24664) **A, B** abdomen, ventral and lateral views **C, D** cleared genitalia, ventral and dorsal views. Scale bars: 0.5 mm.

Distinguished from similar species in the northern Saudi Arabian group (*M.bashayer* sp. nov., *M.maysaan* sp. nov.) by very slender main bulbal process (Fig. 40D; wider in other species), and by epigynal ‘knob’ in posterior rather than central position on epigynal plate (Fig. 42A, B); from *M.maysaan* sp. nov. also by less widened hinged process of procursus (Fig. 40C); from species of the southern Saudi Arabian group and *M.jacominae* by shorter male palpal trochanter apophysis (Fig. 39C), internal female genitalia with membranous central element rather than distinct arc (Fig. 41C), and without crescent-shaped structures.

##### Description.

**Male** (holotype). ***Measurements*.** Total body length 3.4, carapace width 1.2. Distance PME-PME 210 µm; diameter PME 80 µm; distance PME-ALE 20 µm; distance AME-AME 20 µm; diameter AME 45 µm. Leg 1: 28.7 (7.0 + 0.5 + 7.1 + 12.7 + 1.4), tibia 2: 4.5, tibia 3: 2.9, tibia 4: 4.0; tibia 1 L/d: 71; diameters of leg femora (at half length) 0.11–0.12; of leg tibiae 0.10.

***Colour*** (in ethanol). Carapace pale ochre-yellow with distinct brown mark, ocular area not darkened, clypeus with very indistinct darker pattern; sternum monochromous whitish; legs ochre-yellow to light brown, patella dark brown, tibia-metatarsus joints with small brown ring, femur 1 ventrally proximally brown (less distinct also femur 2); abdomen pale ochre-grey, with indistinct darker internal marks.

***Body*.** Habitus as in Fig. 3F. Ocular area slightly raised (distinct in frontal view). Carapace without thoracic groove. Clypeus unmodified. Sternum wider than long (0.74/0.58), unmodified. Abdomen oval, approximately twice as long as wide. Gonopore with four epiandrous spigots (Fig. 10A). Spinnerets as in Fig. 9F, G.

***Chelicerae*.** As in Fig. 41A, B; with pair of distal apophyses near laminae, each with two cone-shaped hairs (Fig. 6C); with pair of distinct proximal frontal apophyses; with pair of very low and indistinct lateral humps.

***Palps*.** As in Fig. 39; coxa unmodified; trochanter with long ventral apophysis with distinct proximal retrolateral hump and modified hair on distal tip (Fig. 9B); femur distally widened, with subdistal ventral hump; femur-patella joints shifted toward prolateral side; tibia-tarsus joints shifted toward retrolateral side; tarsus with large tarsal organ. Procursus (Fig. 40A–C) proximally with sclerotised prolateral hump; at half length with prolateral sclerotised ridge transforming distally into transparent membrane, and brush of dorsal hairs; distally with small retrolateral ridge, large bifid membranous ventral-prolateral flap (Fig. 7C, D), and dorsal hinged process. Genital bulb (Fig7F, G, 40D, E) with strong proximal sclerite; with two sclerotised processes of unclear homology: prolateral process long and slender, with small, pointed branch on retrolateral side; retrolateral process simple, originating from basis of embolus and directed parallel to prolateral process; and mostly semi-transparent short embolus with membranous extensions.

***Legs*.** Without spines, without curved hairs, without sexually dimorphic short vertical hairs; retrolateral trichobothrium of tibia 1 at 6%; prolateral trichobothrium absent on tibia 1; tarsus 1 with > 20 pseudosegments, distally distinct.

***Variation*** (male). Tibia 1 in 21 other males: 5.1–7.3 (mean 6.5). Carapace pattern very consistent. Abdomen usually with large white marks dorsally and laterally.

**Female.** In general very similar to male but anterior leg femora proximally not darkened; ocular area with large median and small lateral brown marks. Tibia 1 in 24 females: 4.4–5.9 (mean 5.2). Epigynum (Figs 10B, 42A, B) protruding, anterior plate oval, mostly dark brown except medially posteriorly, with small knob-shaped process (Fig. 11A) near posterior margin; posterior epigynal plate very short and indistinct, light brown. Internal genitalia (Figs 41C, 42C, D) with pair of elongated pore plates in transversal position; with pair of lateral sclerites and complex system of membranous structures.

##### Etymology.

The species name is derived from the type locality; noun in apposition.

##### Distribution.

Known from type locality only, in Saudi Arabia, ‘Asir Province (Fig. 13C).

##### Natural history.

The spiders were found in caverns among and under boulders, often together with a representative of *Smeringopus* Simon, 1890 (Araneae: Pholcidae). Both species sometimes occurred in very high densities. In one case, a ceiling of a cave was estimated to measure ~ 3 m^2^ and to contain ~ 250 large (adult and penultimate instar) specimens (i.e., with average distances between specimens of ~ 10 cm) (Fig. 15). One egg sac had a diameter of 2.5 mm, and contained ~ 35 eggs. Egg diameters ranged from 0.69 to 0.71 mm.

#### ﻿﻿ Micropholcus bashayer

Taxon classificationAnimaliaAraneaePholcidae

Huber sp. nov.

C76D47CD-DD4A-5269-90C1-285F36B5752E

https://zoobank.org/3B85B5E5-99F7-46EB-B1D0-0380D127FC33

**Figure 43.**
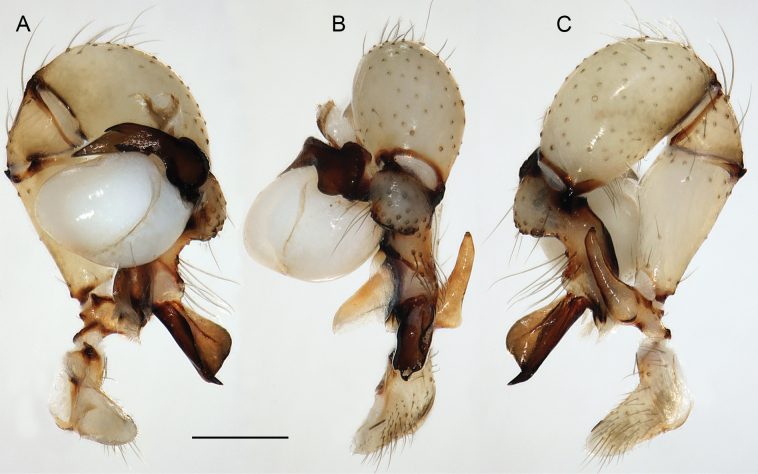
*Micropholcusbashayer* Huber, sp. nov.; male from Saudi Arabia, ‘Asir, NW of Al Bashayer (ZFMK Ar 24665). Left palp in prolateral (**A**), dorsal (**B**), and retrolateral (**C**) views. Scale bar: 0.3 mm.

**Figure 44.**
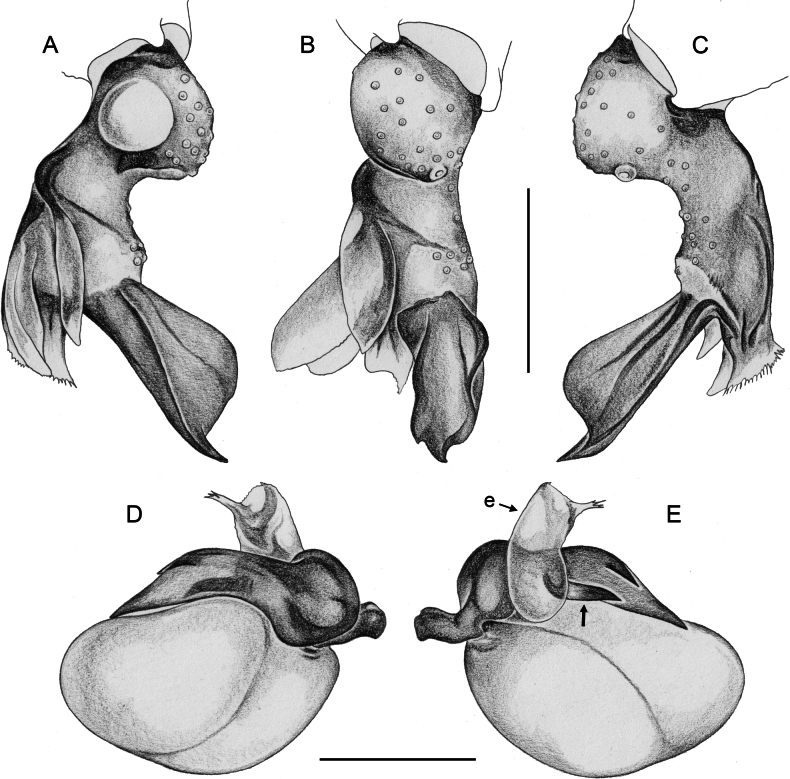
*Micropholcusbashayer* Huber, sp. nov.; male from Saudi Arabia, ‘Asir, NW of Al Bashayer (ZFMK Ar 24665) **A–C** left procursus in prolateral, dorsal, and retrolateral views **D, E** left genital bulb in prolateral and retrolateral views; bold arrow in E points at retrolateral process originating from embolus. Abbreviation: e, embolus. Scale bars: 0.3 mm.

**Figure 45.**
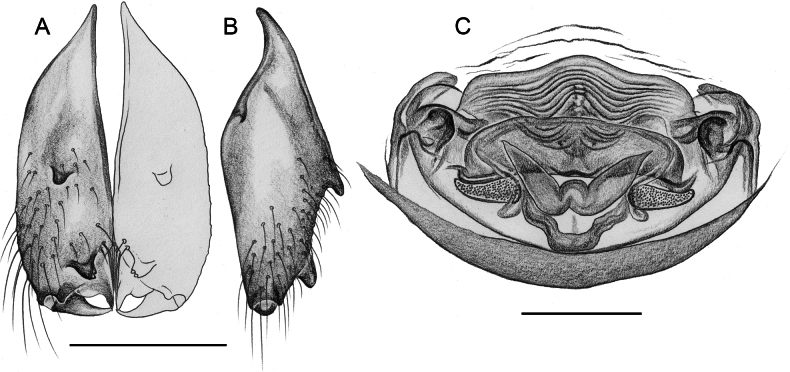
*Micropholcusbashayer* Huber, sp. nov.; from Saudi Arabia, ‘Asir, NW of Al Bashayer **A, B** male chelicerae, frontal and lateral views (ZFMK Ar 24665) **C** cleared female genitalia, dorsal view (ZFMK Ar 24666). Scale bars: 0.3 mm.

**Figure 46.**
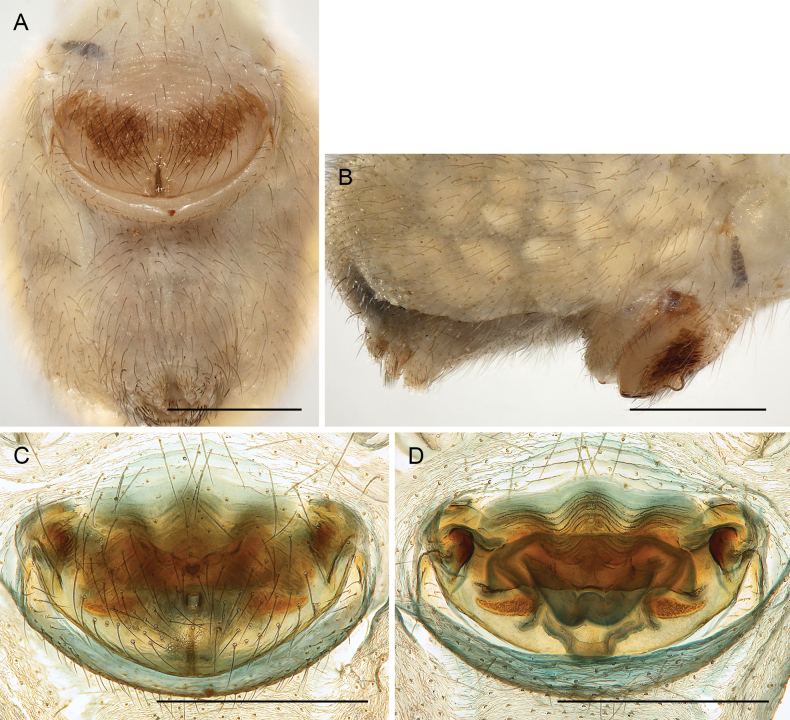
*Micropholcusbashayer* Huber, sp. nov.; female from Saudi Arabia, ‘Asir, NW of Al Bashayer (ZFMK Ar 24666) **A, B** abdomen, ventral and lateral views **C, D** cleared genitalia, ventral and dorsal views. Scale bars: 0.5 mm.

Figs 3G , 43 , 44 , 45 , 46

##### Type material.

***Holotype*.** Saudi Arabia – ‘**Asir** • ♂; NW of Al Bashayer; 19.8194°N, 41.8824°E; 1850 m a.s.l.; 19 Mar. 2024; B.A. Huber leg.; KSMA. ***Paratypes*.** Saudi Arabia – ‘**Asir** • 8 ♂♂, 10 ♀♀; same collection data as for holotype; ZFMK Ar 24665 to 24666.

##### Other material.

Saudi Arabia – ‘**Asir** • 2 ♂♂, 3 ♀♀; in pure ethanol; same collection data as for holotype; ZFMK SA96.

##### Diagnosis.

Distinguished from most similar known species (*M.maysaan* sp. nov.) by less widened dorsal hinged process of procursus (Fig. 44C), by pointed projection of bulbal process (arrowed in Fig. 44E) not directed towards bulbous part, and by darkened central area of epigynal plate (Fig. 46A; rather than anterior part, cf. Fig. 50A). From third species in northern Saudi Arabian group (*M.tanomah* sp. nov.) by wider main bulbal process (Fig. 44D), by epigynal ‘knob’ in central rather than posterior position on epigynal plate (Fig. 46A). From species of the southern Saudi Arabian group and *M.jacominae* by shorter male palpal trochanter apophysis (Fig. 43C), internal female genitalia with membranous central element rather than distinct arc (Fig. 45C) and without crescent-shaped structures.

##### Description.

**Male** (holotype). ***Measurements*.** Total body length 3.0, carapace width 1.0. Distance PME-PME 195 µm; diameter PME 80 µm; distance PME-ALE 20 µm; distance AME-AME 20 µm; diameter AME 50 µm. Leg 1: 23.7 (6.0 + 0.5 + 5.9 + 10.1 + 1.2), tibia 2: 3.6, tibia 3: 2.3, tibia 4: 3.4; tibia 1 L/d: 66; diameters of leg femora (at half length) 0.10–0.11; of leg tibiae 0.09.

***Colour*** (in ethanol). Carapace pale ochre-yellow with distinct brown mark, ocular area and clypeus also with indistinct darker pattern; sternum monochromous whitish; legs ochre-yellow to light brown, patella dark brown, tibia-metatarsus joints with small brown ring, femur 1 ventrally proximally brown (less distinct also femur 2); abdomen pale ochre-grey, dorsally and laterally with whitish internal marks.

***Body*.** Habitus as in *M.maysaan* sp. nov. (cf. Fig. 3H). Ocular area slightly raised. Carapace without thoracic groove. Clypeus unmodified. Sternum wider than long (0.68/0.48), unmodified. Abdomen oval, approximately twice as long as wide.

***Chelicerae*.** As in Fig. 45A, B; with pair of distal apophyses near laminae, each with two cone-shaped hairs; with pair of distinct proximal frontal apophyses; without proximal lateral processes.

***Palps*.** As in Fig. 43; coxa unmodified; trochanter with long ventral apophysis with distinct proximal retrolateral hump and modified hair on distal tip; femur distally widened, otherwise unmodified; femur-patella joints shifted toward prolateral side; tibia-tarsus joints shifted toward retrolateral side; tarsus with large tarsal organ. Procursus (Fig. 44A–C) proximally with sclerotised prolateral hump; at half length with prolateral sclerotised ridge transforming distally into transparent membrane, and brush of dorsal hairs; distally with small retrolateral ridge, large bifid membranous ventral-prolateral flap, and distinctive dorsal hinged process. Genital bulb (Fig. 44D, E) with strong proximal sclerite; with two sclerotised processes of unclear homology: prolateral process large but simple, with small, pointed side branch; retrolateral process very simple, originating from basis of embolus and directed parallel to prolateral process; and mostly semi-transparent short embolus with membranous extension.

***Legs*.** Without spines, without curved hairs, without sexually dimorphic short vertical hairs; retrolateral trichobothrium of tibia 1 at 6%; prolateral trichobothrium absent on tibia 1; tarsus 1 with > 20 pseudosegments, distally distinct.

***Variation*** (male). Tibia 1 in nine males (incl. holotype): 5.5–6.7 (mean 6.0).

**Female.** In general, very similar to male but anterior leg femora proximally not darkened. Tibia 1 in ten females: 4.4–5.4 (mean 4.9). Epigynum (Fig. 46A, B) protruding, anterior plate oval, with knob-shaped process medially; with large brown mark slightly divided medially, anteriorly light, posteriorly with small median dark line; posterior epigynal plate very short and indistinct. Internal genitalia (Figs 45C, 46C, D) with pair of pore plates in transversal position; with pair of lateral sclerites and complex system of membranous structures.

##### Etymology.

The species name is derived from the type locality; noun in apposition.

##### Distribution.

Known from type locality only, in Saudi Arabia, ‘Asir Province (Fig. 13C).

##### Natural history.

The spiders were found sitting on the undersides of large boulders, in small cave-like spaces between boulder and ground. One egg sac contained approximately 30 eggs, with an egg diameter of 0.60 mm. One female had an acrocerid larva in her book lung (Fig. 76A–D).

#### ﻿﻿ Micropholcus maysaan

Taxon classificationAnimaliaAraneaePholcidae

Huber sp. nov.

5A292739-2C17-5AA8-9FE1-E98C61C94477

https://zoobank.org/F5E999F5-8646-4083-9F4F-9B9943F2201F

**Figure 47.**
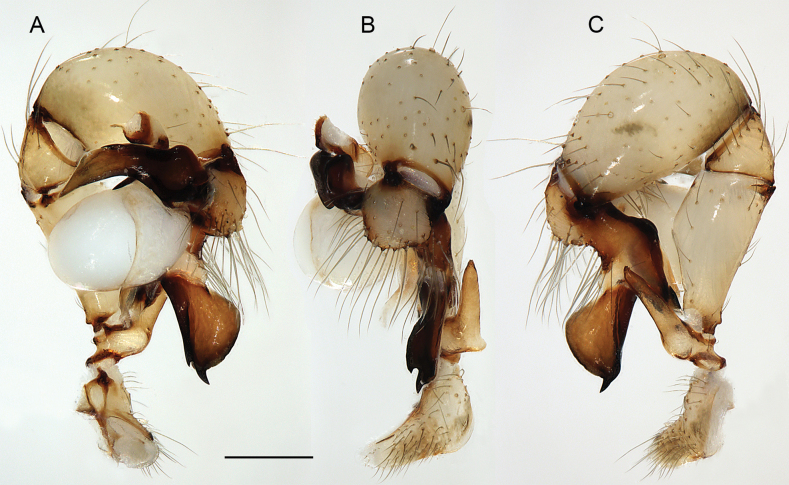
*Micropholcusmaysaan* Huber, sp. nov.; male from Saudi Arabia, Mecca, NW of Maysaan (ZFMK Ar 24667). Left palp in prolateral (**A**), dorsal (**B**), and retrolateral (**C**) views. Scale bar: 0.3 mm.

**Figure 48.**
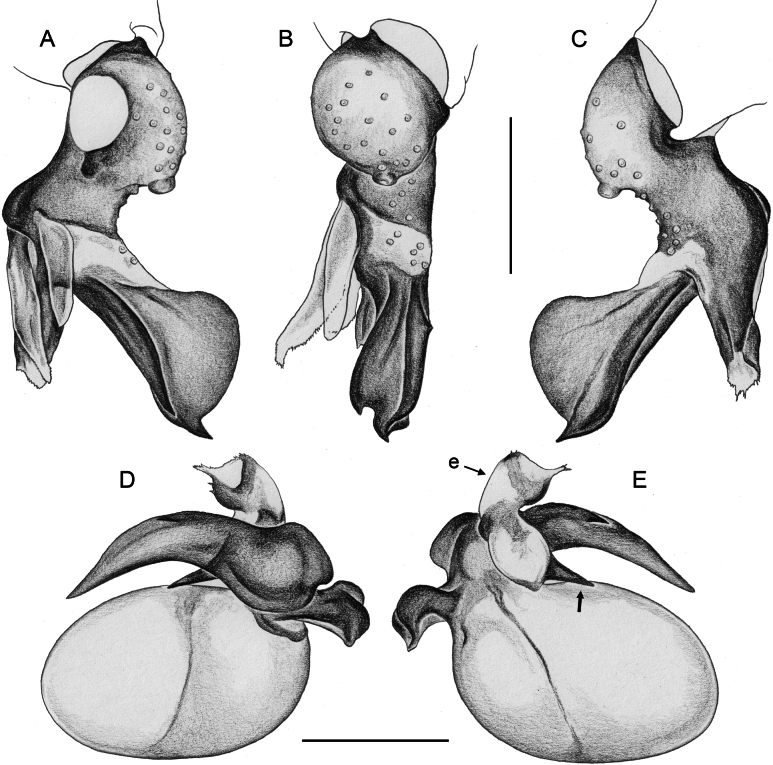
*Micropholcusmaysaan* Huber, sp. nov.; male from Saudi Arabia, Mecca, NW of Maysaan (ZFMK Ar 24667) **A–C** left procursus in prolateral, dorsal, and retrolateral views **D, E** left genital bulb in prolateral and retrolateral views; bold arrow in E points at retrolateral process originating from embolus. Abbreviation: e, embolus. Scale bars: 0.3 mm.

**Figure 49.**
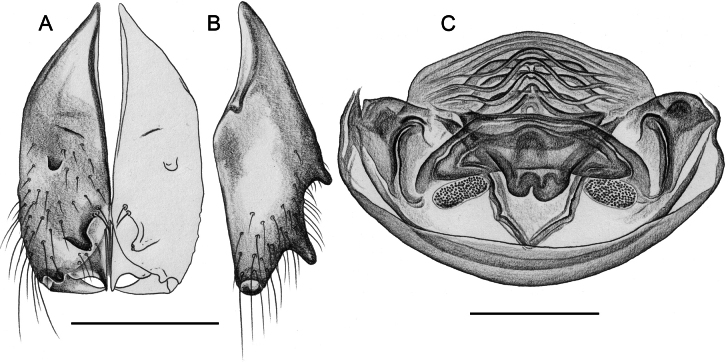
*Micropholcusmaysaan* Huber, sp. nov.; male from Saudi Arabia, Mecca, NW of Maysaan (ZFMK Ar 24667), female from Saudi Arabia, Al Bahah, NW of Al Bahah (ZFMK Ar 24669) **A, B** male chelicerae, frontal and lateral views **C** cleared female genitalia, dorsal view. Scale bars: 0.3 mm.

**Figure 50.**
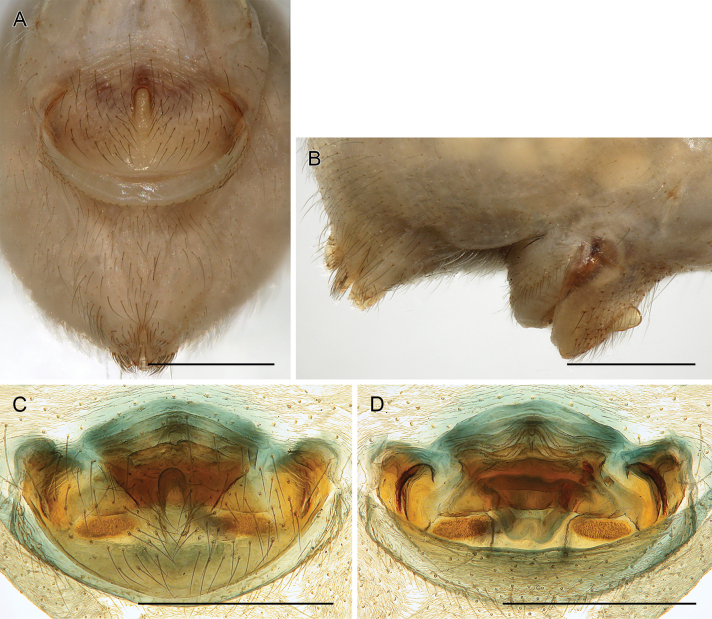
*Micropholcusmaysaan* Huber, sp. nov.; female from Saudi Arabia, Mecca, NW of Maysaan (ZFMK Ar 24668) **A, B** abdomen, ventral and lateral views **C, D** cleared genitalia, ventral and dorsal views. Scale bars. 0.5 mm.

**Figure 51.**
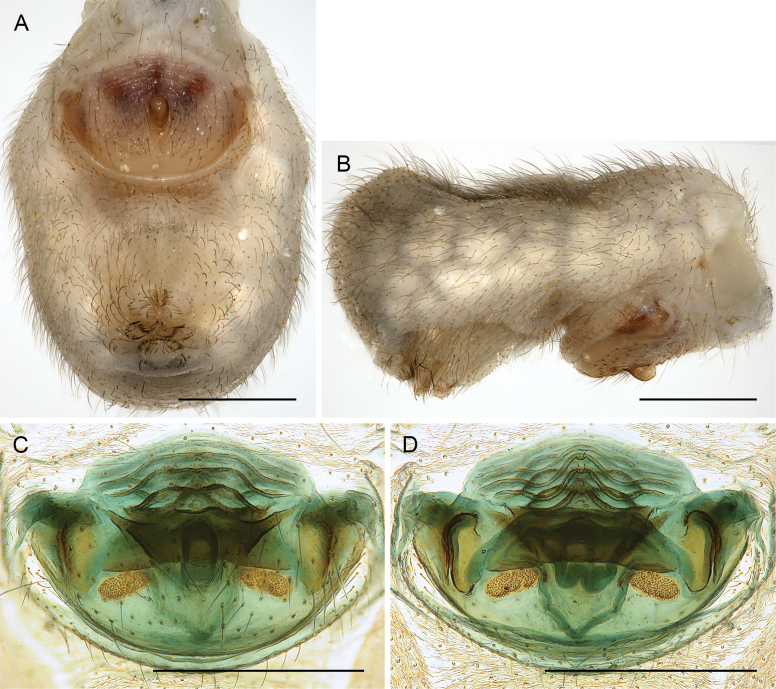
*Micropholcusmaysaan* Huber, sp. nov.; female from Saudi Arabia, Mecca, NW of Al Bahah (ZFMK Ar 24669) **A, B** abdomen, ventral and lateral views **C, D** cleared genitalia, ventral and dorsal views. Scale bars: 0.5 mm.

Figs 3H , 47 , 48 , 49 , 50 , 51

##### Type material.

***Holotype*.** Saudi Arabia – **Mecca** • ♂; NW of Maysaan; 20.7717°N, 40.7985°E; 2560 m a.s.l.; 29 Mar. 2024; B.A. Huber leg.; KSMA. ***Paratypes*.** Saudi Arabia – **Mecca** • 4 ♂♂, 5 ♀♀; same collection data as for holotype; ZFMK Ar 24667 to 24668.

##### Other material.

Saudi Arabia – **Mecca** • 2 ♀♀, 4 juvs; in pure ethanol; same collection data as for holotype; ZFMK SA140 – **Al Bahah** • 2 ♂♂, 6 ♀♀; NW of Al Bahah, ‘site 2’; 20.2095°N, 41.3700°E; 2250 m a.s.l.; 16 Mar. 2024; B.A. Huber leg.; ZFMK Ar 24669 • 1 ♂, 4 ♀♀; in pure ethanol; same collection data as for preceding; ZFMK SA88 • 1 ♂; S of Al Bahah, ‘site 2’; 19.9896°N, 41.4373°E; 1250 m a.s.l.; 17 Mar. 2024; B.A. Huber leg.; ZFMK Ar 24670.

##### Diagnosis.

Distinguished from other species in northern Saudi Arabian group (*M.bashayer* sp. nov., *M.tanomah* sp. nov.) by strongly widened dorsal hinged process of procursus (Fig. 48C), by pointed process originating from embolus directed towards bulbous part (arrowed in Fig. 48E); from *M.bashayer* sp. nov. also by darkened anterior area of epigynal plate (Fig. 50A; rather than central area, cf. Fig. 46A); from *M.tanomah* sp. nov. also by wider main bulbal process (Fig. 48D), and by epigynal ‘knob’ in central rather than posterior position on epigynal plate (Figs 50A, B, 51A, B). From species of the southern Saudi Arabian group and *M.jacominae* by shorter male palpal trochanter apophysis (Fig. 47C), internal female genitalia with membranous central element rather than distinct arc (Fig. 49C), and without crescent-shaped structures.

##### Description.

**Male** (holotype). ***Measurements*.** Total body length 3.1, carapace width 1.1. Distance PME-PME 200 µm; diameter PME 90 µm; distance PME-ALE 30 µm; distance AME-AME 40 µm; diameter AME 50 µm. Leg 1: 26.3 (6.6 + 0.5 + 6.6 + 11.3 + 1.3), tibia 2: 4.2, tibia 3: 2.7, tibia 4: 3.7; tibia 1 L/d: 69; diameters of leg femora (at half length) 0.10–0.11; of leg tibiae 0.09–0.10.

***Colour*** (in ethanol). Carapace pale ochre-yellow with distinct brown mark, clypeus also with indistinct darker pattern; sternum monochromous whitish; legs ochre-yellow to light brown, patella dark brown, tibia-metatarsus joints with small brown ring, femur 1 ventrally proximally brown (less distinct also femur 2); abdomen pale ochre-grey, dorsally and laterally with whitish internal marks.

***Body*.** Habitus as in Fig. 3H. Ocular area slightly raised. Carapace without thoracic groove. Clypeus unmodified. Sternum wider than long (0.72/0.58), unmodified. Abdomen oval, approximately twice as long as wide.

***Chelicerae*.** As in Fig. 49A, B; with pair of distal apophyses near laminae, each with two cone-shaped hairs; with pair of distinct proximal frontal apophyses; without proximal lateral processes.

***Palps*.** As in Fig. 47; coxa unmodified; trochanter with long ventral apophysis with distinct proximal retrolateral hump and modified hair on distal tip; femur distally widened, otherwise unmodified; femur-patella joints shifted toward prolateral side; tibia-tarsus joints shifted toward retrolateral side; tarsus with large tarsal organ. Procursus (Fig. 48A–C) proximally with sclerotised prolateral hump; at half length with prolateral sclerotised ridge transforming distally into transparent membrane, and brush of dorsal hairs; distally with small retrolateral ridge, large bifid membranous ventral-prolateral flap, and distinctively widened dorsal hinged process. Genital bulb (Fig. 48D, E) with strong proximal sclerite; with two sclerotised processes of unclear homology: prolateral process large but simple, with small, pointed side branch; retrolateral process very simple, originating from basis of embolus and pointing towards globular part of bulb; and mostly semi-transparent short embolus with membranous extensions.

***Legs*.** Without spines, without curved hairs, without sexually dimorphic short vertical hairs; retrolateral trichobothrium of tibia 1 at 6%; prolateral trichobothrium absent on tibia 1; tarsus 1 with ~ 20 pseudosegments, distally distinct.

***Variation*** (male). Tibia 1 in eight males (incl. holotype): 5.1–7.1 (mean 6.1). Males from NW of Maysaan and from NW of Al Bahah appear essentially identical. In the single male from S of Al Bahah, the shape of the dorsal hinged process of the procursus is slightly different: its widest point is at one third of its length rather than at half-length. Also, the retrolateral bulbal process in this specimen is (in prolateral view) less strongly protruding from behind the prolateral process.

**Female.** In general, very similar to male but anterior leg femora proximally not darkened. Tibia 1 in ten females: 4.6–5.5 (mean 4.9). Epigynum (Fig. 50A, B) protruding, anterior plate oval, with prominent knob-shaped process medially; posterior epigynal plate very short and indistinct. Internal genitalia (Figs 49C, 50C, D, 51C, D) with pair of oval pore plates in transversal position; with pair of lateral sclerites and complex system of membranous structures. Females from NW of Maysaan and from NW of Al Bahah appear essentially identical. No female is available from S of Al Bahah.

##### Etymology.

The species name is derived from the type locality; noun in apposition.

##### Distribution.

Known from three localities in Saudi Arabia, in Mecca and Al Bahah Provinces (Fig. 13C).

##### Natural history.

At the type locality, the spiders were found in small caverns under large boulders on a hill (Fig. 14D), where they were tightly pressed against the rock on the ceiling. Upon disturbance, they were very reluctant to move; only when the sparse sheet of silk covering the spider was removed, they started to run away over the rock. NW of Al Bahah most specimens were collected from a small hole under a flat rock; distances between specimens ranged from ~ 10–20 cm. One egg sac contained ~ 12 eggs, with an egg diameter of 0.62 mm.

#### ﻿﻿ Micropholcus darbat

Taxon classificationAnimaliaAraneaePholcidae

Huber sp. nov.

6B6E955B-D692-5045-8195-FAFE001AD780

https://zoobank.org/A7E37832-2C7E-43AA-B85F-DDFCCBA26009

Figs 4A, B , 5B , 6D, E , 7H , 8A–C , 9C , 10C, D , 11B, G, H , 52 , 53 , 54 , 55

Micropholcus sp. n. Om74 – Huber and Eberle 2021, Suppl. material 1.

##### Type material.

***Holotype*.** Oman – **Dhofar** • ♂; Wadi Darbat; between 17.086°N, 54.444°E and 17.095°N, 54.452°E; 200–230 m a.s.l., 23 Feb. 2018; B.A. Huber leg.; ZFMK Ar 24671.

##### Other material.

Oman – **Dhofar** • 9 ♂♂, 7 ♀♀, 1 juv. (1 ♂, 1 ♀ used for SEM); same collection data as for holotype; ZFMK Ar 24672, 24699 • 1 ♂, 3 ♀♀, in pure ethanol; same collection data as for holotype; ZFMK Om133 • 2 ♂♂, 2 ♀♀; Ain Athoom; 17.1185°N, 54.3667°E; 280 m a.s.l.; in small cave; 28 Feb. 2018; B.A. Huber leg.; ZFMK Ar 24673 • 1 ♂, 1 ♀, in pure ethanol; same collection data as for preceding; ZFMK Om147 • 3 ♂♂, 1 ♀; near Qairoon Hairitti; 17.2600°N, 54.0808°E; 845 m a.s.l.; in small cave; 27 Feb. 2018; B.A. Huber leg.; ZFMK Ar 24674 • 2 ♀♀, in pure ethanol; same collection data as for preceding; ZFMK Om146 • 3 ♂♂, 5 ♀♀, 1 juv.; Wadi Nahiz; 17.140°N, 54.123°E; 140 m a.s.l.; in small caverns; 26 Feb. 2018; B.A. Huber leg.; ZFMK Ar 24675 • 1 ♀, 3 juvs, in pure ethanol; same collection data as for preceding; ZFMK Om142.

##### Diagnosis.

**Figure 52.**
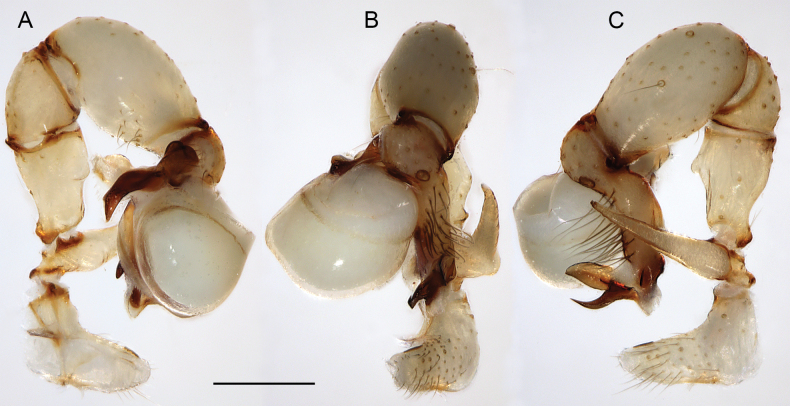
*Micropholcusdarbat* Huber, sp. nov.; male from Oman, Dhofar, Wadi Darbat (ZFMK Ar 24672). Left palp in prolateral (**A**), dorsal (**B**), and retrolateral (**C**) views. Scale bar: 0.3 mm.

**Figure 53.**
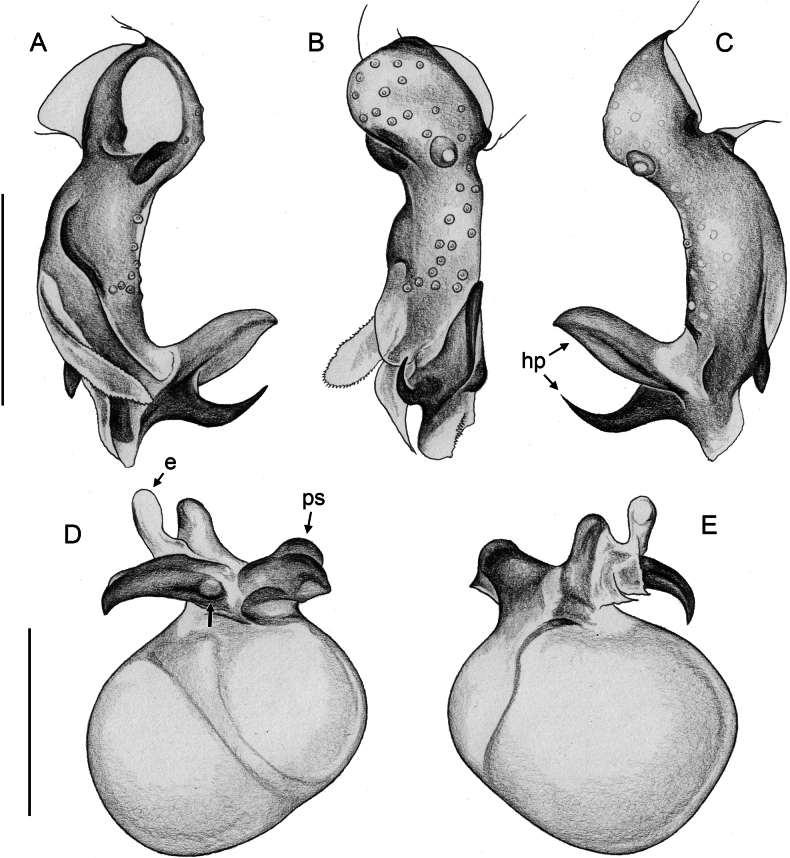
*Micropholcusdarbat* Huber, sp. nov.; male from Oman, Dhofar, Wadi Darbat (ZFMK Ar 24672) **A–C** left procursus in prolateral, dorsal, and retrolateral views **D, E** left genital bulb in prolateral and retrolateral views; bold arrow in D points at proximal hump on prolateral bulbal process. Abbreviations: e, embolus; hp, dorsal hinged process; ps, proximal bulbal sclerite. Scale bars: 0.3 mm.

**Figure 54.**
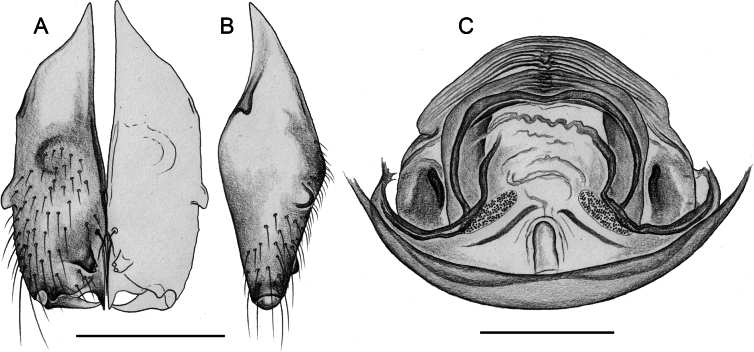
*Micropholcusdarbat* Huber, sp. nov.; from Oman, Dhofar, Wadi Darbat (ZFMK Ar 24672) **A, B** male chelicerae, frontal and lateral views **C** cleared female genitalia, dorsal view. Scale bars: 0.3 mm.

**Figure 55.**
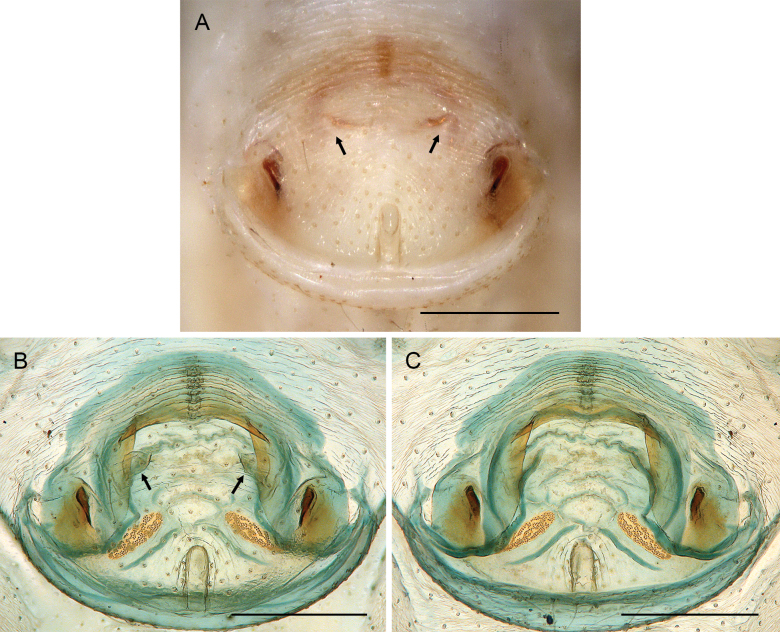
*Micropholcusdarbat* Huber, sp. nov.; female from Oman, Dhofar, Wadi Darbat (ZFMK Ar 24672) **A** epigynum, ventral view **B, C** cleared female genitalia, ventral and dorsal views. Arrows point at distinctive internal pockets. Scale bars: 0.3 mm.

Males are easily distinguished from known congeners by shape of procursus with distinctive dorsal hinged process split into two branches (Fig. 53C); also by shapes of bulbal processes (Fig. 53D, E; prolateral apophysis simple, with small proximal prolateral hump) and cheliceral processes (Fig. 54A, B; proximal frontal processes very low and indistinct); from geographically close *M.shaat* sp. nov. also by longer trochanter apophysis without distinct proximal process (Fig. 52C). Females differ from known congeners by pair of internal pockets visible also in uncleared specimens (arrows in Fig. 55); from geographically close *M.shaat* sp. nov. also by epigynum without median sclerotised band (Fig. 55A), pore plates oval and converging anteriorly (Fig. 55C), and internal genitalia without large membranous sac.

##### Description.

**Male** (holotype). ***Measurements*.** Total body length 3.2, carapace width 1.2. Distance PME-PME 250 µm; diameter PME 90 µm; distance PME-ALE 20 µm; distance AME-AME 15 µm; diameter AME 55 µm. Leg 1: 36.5 (9.1 + 0.6 + 9.1 + 16.1 + 1.6), tibia 2: 5.9, tibia 3: 3.8, tibia 4: 5.1; tibia 1 L/d: 83; diameters of leg femora (at half length) 0.12–0.13; of leg tibiae 0.11.

***Colour*** (in ethanol). Prosoma and legs pale ochre-yellow, carapace with brown median mark; legs with darkened patellae and tibia-metatarsus joints; abdomen pale grey to whitish.

***Body*.** Habitus as in Fig. 4A. Ocular area raised (distinct in frontal view; Fig. 5B). Carapace without thoracic groove. Clypeus unmodified. Sternum wider than long (0.75/0.60), unmodified. Abdomen oval, approximately twice as long as wide. Gonopore with four epiandrous spigots (Fig. 10C).

***Chelicerae*.** As in Figs 6D, 54A, B; with pair of distal apophyses near laminae, each with two cone-shaped hairs (Fig. 6E); pair of proximal lateral processes weakly sclerotised and directed towards distal; and pair of very low proximal frontal humps.

***Palps*.** As in Fig. 52; coxa unmodified; trochanter with long ventral apophysis with retrolateral hump at basis and modified hair at tip (Fig. 9C); femur proximally with low dorsal hump, distally with weakly sclerotised rounded process on prolateral-ventral side; femur-patella joints shifted toward prolateral side; tibia-tarsus joints shifted toward retrolateral side; tarsus with large tarsal organ. Procursus (Figs 7H, 53A–C) proximally with sclerotised prolateral hump; at half length with prolateral sclerotised ridge transforming distally into transparent membrane, and dense brush of dorsal hairs; distally with small retrolateral ridge, ventral apophysis directed towards prolateral, membranous ventral-prolateral flap (Fig. 8A, B), and distinctive dorsal hinged process split into two branches. Genital bulb (Figs 8C, 53D, E) with strong proximal sclerite, prolateral sclerite simple with small proximal prolateral hump, simple retrolateral sclerite, and mostly semi-transparent short embolus.

***Legs*.** Without spines, without curved hairs, without sexually dimorphic short vertical hairs (most hairs missing in holotype but confirmed in males from near Qairoon Hairitti); retrolateral trichobothrium of tibia 1 at 6%; prolateral trichobothrium absent on tibia 1; tarsus 1 with > 30 pseudosegments, distally distinct.

***Variation*** (male). Tibia 1 in 16 males (incl. holotype): 6.9–9.2 (mean 8.2). Distance between eye triads 190–250 µm. Some males with white marks dorsally on abdomen.

**Female.** In general very similar to male but abdomen often much wider, ocular area slightly less raised and triads closer together (PME-PME 180–190 µm). Tibia 1 in 13 females: 5.7–7.1 (mean 6.3). Epigynum (Figs 10D, 55A) anterior plate oval, protruding, with membranous knob (Fig. 11B) in posterior position and slightly directed towards anterior; lateral internal sclerites clearly visible in untreated specimens; posterior epigynal plate very short and indistinct. Internal genitalia (Fig. 54C, 55B, C) with pair of oval pore plates converging anteriorly, pair of lateral sclerites and pair of ventral pockets (arrows in Fig. 55); with sclerotised anterior arc.

##### Etymology.

The species name is derived from the type locality; noun in apposition.

##### Distribution.

Known from several localities in Dhofar, western Oman (Fig. 13C).

##### Natural history.

In Wadi Darbat and Wadi Nahiz, the spiders were abundant on the vertical rocks and rock shelters lining the valleys. They were tightly pressed against the rock surface, making them difficult to spot. Upon disturbance, they ran away or dropped to the ground. Near Qairoon Hairitti, the spiders were collected in a small and shallow cave. At Ain Athoom, most specimens were found in a small cave, but juveniles were also found under rocks in the neighbouring area. Two egg sacs contained 21 and 27 eggs, respectively, with an egg diameter of 0.59 mm (Huber and Eberle 2021). One male had an acrocerid larva in his book lung (Fig. 76E, F).

#### ﻿﻿ Micropholcus shaat

Taxon classificationAnimaliaAraneaePholcidae

Huber sp. nov.

2B518129-7C0F-56DF-AC13-C1E3E46B1846

https://zoobank.org/03BCF62A-51BC-46EF-9CA1-FC2B8720C36C

**Figure 56.**
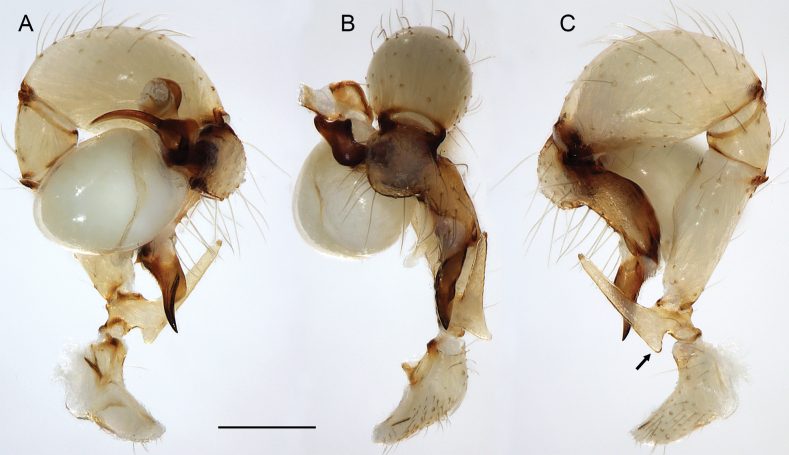
*Micropholcusshaat* Huber, sp. nov.; male from Oman, Dhofar, Shaat sinkhole (ZFMK Ar 24677). Left palp in prolateral (**A**), dorsal (**B**), and retrolateral (**C**) views; arrow in C points at distinctive process proximally on trochanter apophysis. Scale bar: 0.3 mm.

**Figure 57.**
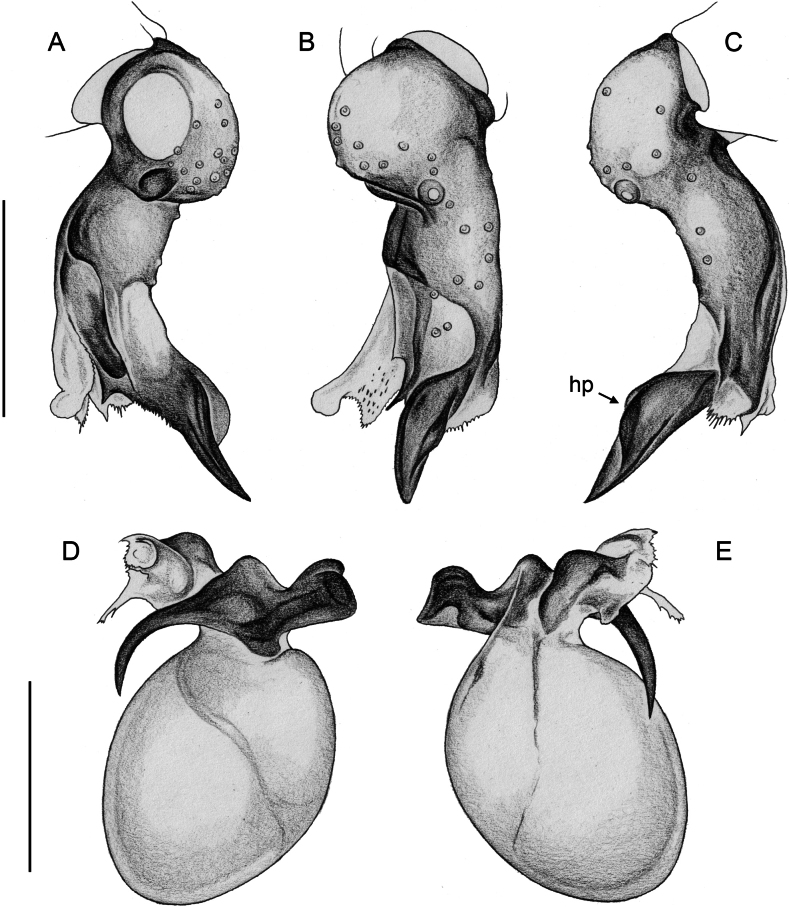
*Micropholcusshaat* Huber, sp. nov.; male from Oman, Dhofar, Shaat sinkhole (ZFMK Ar 24677) **A–C** left procursus in prolateral, dorsal, and retrolateral views **D, E** left genital bulb in prolateral and retrolateral views. Abbreviation: hp, dorsal hinged process. Scale bars: 0.3 mm.

**Figure 58.**
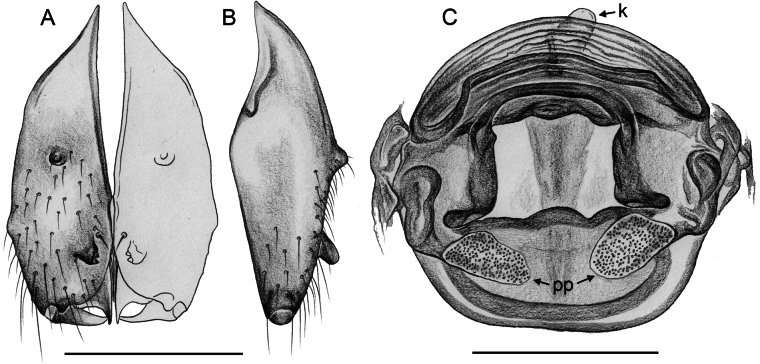
*Micropholcusshaat* Huber, sp. nov.; from Oman, Dhofar, Shaat sinkhole (ZFMK Ar 24677) **A, B** male chelicerae, frontal and lateral views **C** cleared female genitalia, dorsal view. Abbreviations: k, epigynal ‘knob’; pp, pore plates. Scale bars: 0.3 mm.

**Figure 59.**
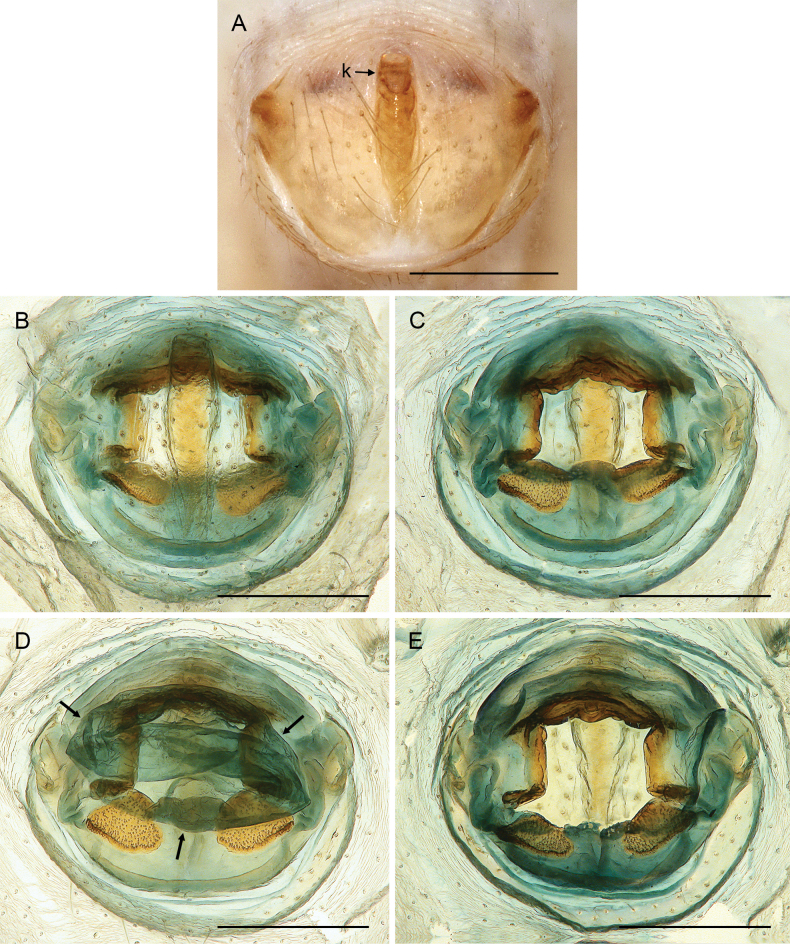
*Micropholcusshaat* Huber, sp. nov.; two females from Oman, Dhofar, Shaat sinkhole (ZFMK Ar 24677) **A** epigynum, ventral view **B, C** cleared female genitalia, ventral and dorsal views, same specimen as in A; membranous sac removed **D, E** cleared female genitalia of second female, dorsal views; membranous sac is shown in D (arrows), but was removed for E. Abbreviation: k, epigynal ‘knob’. Scale bars: 0.3 mm.

Figs 4C , 56 , 57 , 58 , 59

##### Type material.

***Holotype*.** Oman – **Dhofar** • ♂; Shaat sinkhole, in wadis leading to sinkhole; 16.774°N, 53.587°E; 850 m a.s.l.; 25 Feb. 2018; B.A. Huber leg.; ZFMK Ar 24676.

##### Other material.

Oman – **Dhofar** • 4 ♂♂, 2 ♀♀, 1 juv.; same collection data as for holotype; ZFMK Ar 24677 • 1 ♀, 1 juv.; same collection data as for holotype but 24 Feb. 2018; ZFMK Ar 24678 • 3 ♀♀, in pure ethanol; same collection data as for holotype but 24–25 Feb. 2018; ZFMK Om137.

##### Diagnosis.

Males are easily distinguished from known congeners by several details of male palp (Figs 56, 57; proximal process on trochanter; slender femur without distinct processes; procursus with simple dorsal hinged process; prolateral sclerite on genital bulb simple and slender). Females are easily distinguished from known congeners by anterior position of epigynal ‘knob’ (Fig. 58C), by distinctive sclerotised band medially on epigynal plate (Fig. 59A), and by presence of large membranous sac in internal genitalia (Fig. 59D); from geographically close *M.darbat* sp. nov. also by larger and wider pore plates (Fig. 58C).

##### Description.

**Male** (holotype). ***Measurements*.** Total body length 2.6, carapace width 0.9. Distance PME-PME 260 µm; diameter PME 85 µm; distance PME-ALE 15 µm; distance AME-AME 20 µm; diameter AME 45 µm. Leg 1: 27.3 (6.8 + 0.5 + 6.9 + 11.7 + 1.4), tibia 2: 4.3, tibia 3: 2.6, tibia 4: 3.6; tibia 1 L/d: 81; diameters of leg femora (at half length) 0.09–0.10; of leg tibiae 0.08–0.09.

***Colour*** (in ethanol). Prosoma and legs ochre-yellow, carapace with brown median mark; leg femora 1 and 2 proximally darkened; legs with darkened patellae and tibia-metatarsus joints; abdomen pale ochre-grey.

***Body*.** Habitus as in Fig. 4C. Ocular area raised (distinct in frontal view). Carapace without thoracic groove. Clypeus unmodified. Sternum wider than long (0.65/0.50), unmodified. Abdomen oval, approximately twice as long as wide.

***Chelicerae*.** As in Fig. 58A, B; with pair of distal apophyses near laminae, each with two cone-shaped hairs; pair of lateral, indistinct, weakly sclerotised humps; and pair of distinct proximal frontal apophyses.

***Palps*.** As in Fig. 56; coxa unmodified; trochanter with long ventral apophysis with distinct proximal process directed towards coxa; femur slender, distally widened, with indistinct ventral hump at half length; femur-patella joints shifted toward prolateral side; tibia-tarsus joints shifted toward retrolateral side; tarsus with large tarsal organ. Procursus (Fig. 57A–C) proximally with sclerotised prolateral hump; at half length with prolateral sclerotised ridge transforming distally into transparent membrane, and brush of dorsal hairs; distally with small retrolateral ridge, bifid membranous ventral-prolateral flap, and distinctive dorsal hinged process. Genital bulb (Fig. 57D, E) with strong proximal sclerite, prolateral process simple and slender, and short embolus proximally sclerotised, distally with transparent extensions.

***Legs*.** Without spines, without curved hairs, without sexually dimorphic short vertical hairs; retrolateral trichobothrium of tibia 1 at 6%; prolateral trichobothrium absent on tibia 1; tarsus 1 with > 25 pseudosegments, distally distinct.

***Variation*** (male). Tibia 1 in five males (incl. holotype): 6.2–7.8 (mean 6.8). Distance between eye triads 250–270 µm. Some males with white marks dorsally on abdomen.

**Female.** In general very similar to male but abdomen often wider, ocular area slightly less raised and triads closer together (PME-PME 200 µm). Tibia 1 in five females: 5.2–5.6 (mean 5.3). Epigynum (Fig. 59A) anterior plate roundish, anterior margin weakly curved, posterior margin strongly curved, with distinctive median sclerotised band, membranous knob at anterior end of sclerotised band, directed towards posterior; lateral internal sclerites clearly visible in untreated specimens; posterior epigynal plate very short and indistinct. Internal genitalia (Figs 58C, 59B–E) with pair of large sclerotised pore plates, with roughly square-shaped sclerotised opening leading into large round membranous sac (collapsed in Fig. 59D; removed in Fig. 59B, C, E); with complex system of lateral membranous structures.

##### Etymology.

The species name is derived from the type locality; noun in apposition.

##### Distribution.

Known from type locality only, in Dhofar, western Oman (Fig. 13C).

##### Natural history.

The spiders were found in niches and small caverns in the walls of the wadis leading to Shaat sinkhole (Fig. 14E). They rested in the apex of very fine and poorly visible dome-shaped webs directly on the rock surface.

#### ﻿﻿ Micropholcus agadir

Taxon classificationAnimaliaAraneaePholcidae

(Huber, 2011)

B3B8FA44-8A14-5E89-90ED-8DC1A7F0EAC0

**Figure 60.**
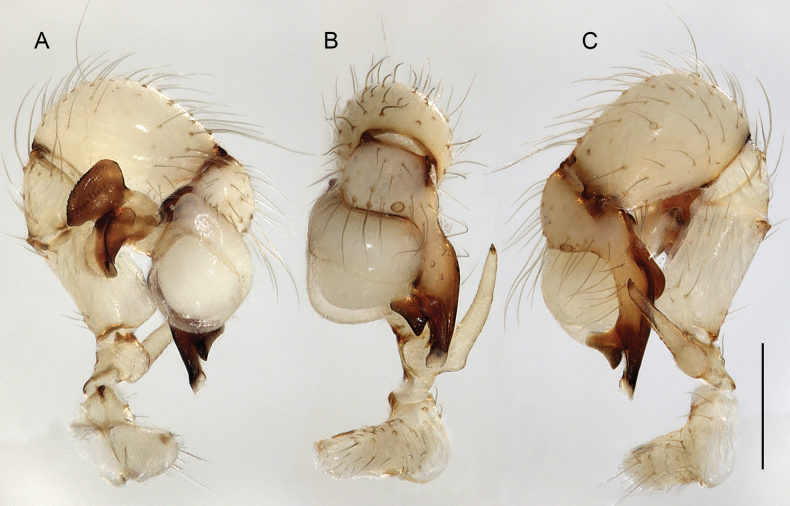
*Micropholcusagadir* (Huber, 2011); male from Morocco, Souss-Massa, Paradise Valley (ZFMK Ar 24679). Left palp in prolateral (**A**), dorsal (**B**), and retrolateral (**C**) views. Scale bar: 0.3 mm.

**Figure 61.**
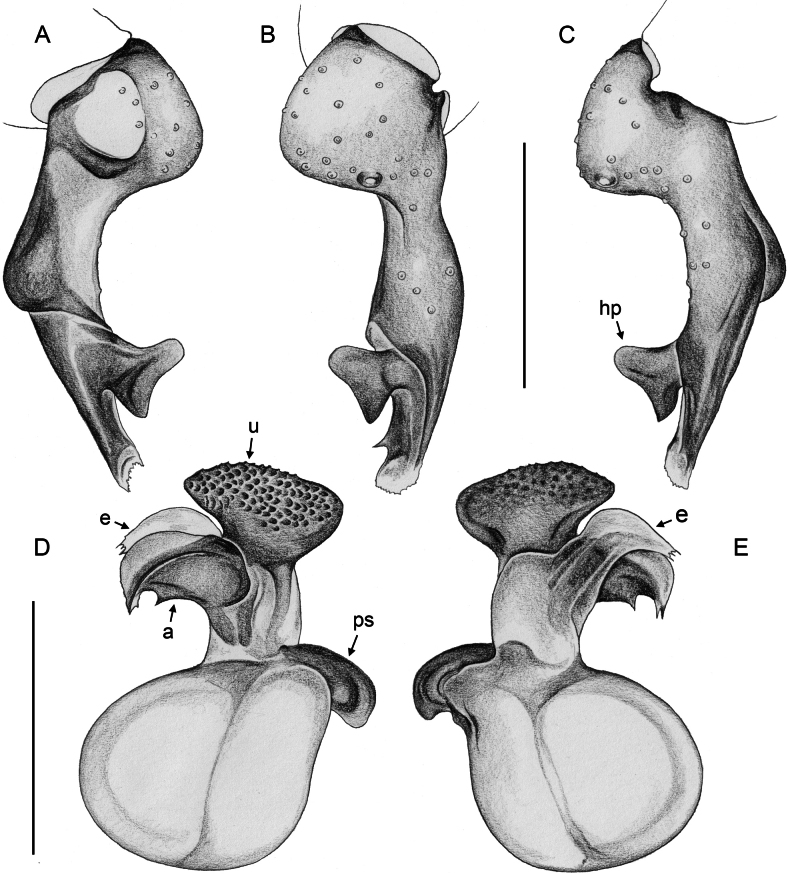
*Micropholcusagadir* (Huber, 2011); male from Morocco, Souss-Massa, Paradise Valley (ZFMK Ar 24679) **A–C** left procursus in prolateral, dorsal, and retrolateral views **D, E** left genital bulb in prolateral and retrolateral views. Abbreviations: a, putative appendix; e, embolus; hp, dorsal hinged process; ps, proximal bulbal sclerite; u, putative uncus. Scale bars: 0.3 mm.

**Figure 62.**
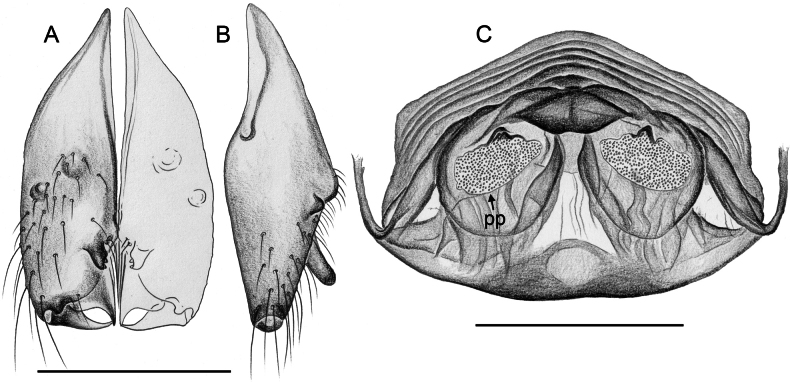
*Micropholcusagadir* (Huber, 2011); male from Morocco, Souss-Massa, Paradise Valley (ZFMK Ar 24679), female from Morocco, Souss-Massa, 7 km N Agadir (IRSB) **A, B** male chelicerae, frontal and lateral views **C** cleared female genitalia, dorsal view (from Huber 2011). Abbreviation: pp, pore plate. Scale bars: 0.3 mm.

**Figure 63.**
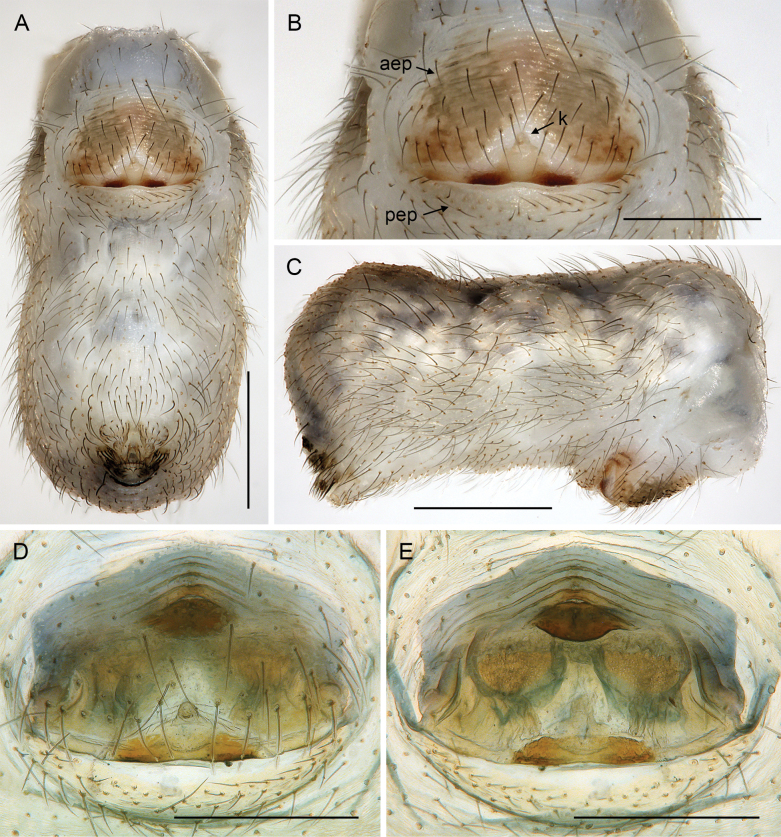
*Micropholcusagadir* (Huber, 2011); female from Morocco, Souss-Massa, Paradise Valley (ZFMK Ar 24679) **A, C** abdomen, ventral and lateral views **B** epigynum, ventral view **D, E** cleared genitalia, ventral and dorsal views. Abbreviations: aep, anterior epigynal plate; k, epigynal ‘knob’; pep, posterior epigynal plate. Scale bars: 0.5 mm (**A, C**); 0.3 mm (**B, D, E**).

Figs 4D, E , 60 , 61 , 62 , 63

Pholcus agadir Huber, 2011: 331, figs 1530–1531, 1553–1554, 1606–1611 (♂♀). Eberle et al. 2018 (molecular data).
Micropholcus agadir – Huber et al. 2018: 83. Huber and Eberle 2021, Suppl. material 1.

##### New records.

Morocco: **Souss-Massa** • 3 ♂♂, 2 ♀♀; Paradise Valley; 30.588°N, 9.528°W; 305 m a.s.l.; 13 Sep. 2018; B.A. Huber leg.; ZFMK Ar 24679 • 2 ♂♂, 2 ♀♀ (one abdomen transferred to ZFMK Ar 24679), in pure ethanol; same collection data as for preceding; ZFMK Mor76 • 2 ♀♀, in pure ethanol; Agadir, path to Kasbah Hill; 30.4289°N, 9.6186°W; 60 m a.s.l.; 28 Nov. 2016; S. Huber leg.; ZFMK Sieg27 • 4 ♀♀, in pure ethanol; Agadir, path to Kasbah Hill; 30.4297°N, 9.6189°W; 110 m a.s.l.; 7 Sep. 2014; S. Huber leg.; ZFMK Sieg11 • 1 ♂, in pure ethanol; Road Agadir-Alma; 30.4864°N, 9.5650°W; 440 m a.s.l.; 27 Nov. 2016; S. Huber leg.; ZFMK Sieg25. **Marrakech-Safi** • 1 ♀; NE of Tizi n’Test; 30.897°N, 8.339°W; 2075 m a.s.l.; 12 Sep. 2018; B.A. Huber leg.; ZFMK Ar 24683.

##### Diagnosis.

Distinguished from similar congeners (*M.tegulifer*, *M.ghar* sp. nov.) by short and distally widened dorsal hinged process of procursus (Fig. 61C), by rounded uncus with scales (Fig. 61D, E; similar only in *M.khenifra* sp. nov., cf. Fig. 69E), and by flat and oval appendix with small proximal spine and prolateral ridge (Fig. 61D); from *M.tegulifer* also by presence of two pairs of processes proximally on male chelicerae (Fig. 62A, B; absent in *M.tegulifer*), by lateral marks on carapace (Fig. 4D, E; absent in *M.tegulifer*), and by oval rather than elongate pore plates in female internal genitalia. From *M.ghar* sp. nov. also distinguished by smaller triangular plate posteriorly on epigynum (compare Fig. 63B with Fig. 67A).

**Description** (amendments; see also Huber 2011). Tibia 1 length in seven males (incl. holotype): 6.0–7.4 (mean 6.7); in 12 females (including those in Huber 2011): 4.8–5.9 (mean 5.6). The drawings in Huber (2011) are from the male holotype and from a topotypical female. Compared to the newly collected males, the procursus of the holotype was slightly twisted towards prolateral; thus, the dorsal hinged process was described as “prolateral branch”. The new material also shows that the dorsal process is connected to the main part of the procursus by slightly membranous cuticle, i.e., it is hinged, comparable with the dorsal processes of congeneric species. The female epigynal knob was originally said to be on the posterior plate (Huber 2011). Instead, it is situated on a small, slightly separate triangular part of the anterior plate. The posterior epigynal plate in *M.agadir* is indistinct (Fig. 63B); it was indicated by a row of hairs in Huber (2011: fig. 1610), but not explicitly drawn.

##### Distribution.

Known from several localities in southern Morocco, in Souss-Massa and Marrakech-Safi regions (Fig. 13D).

##### Natural history.

In Paradise Valley, the spiders were found on overhanging rock-surfaces, often in very close proximity to *Holocnemusreini* (C. Koch, 1873). While the latter had large and distinct webs, the webs of *Micropholcus* were barely visible. Two egg sacs had diameters of 1.9 and 2.4 mm, respectively, contained 23/31 eggs with an egg diameter of 0.60–0.63 (Huber and Eberle 2021).

#### ﻿﻿ Micropholcus ghar

Taxon classificationAnimaliaAraneaePholcidae

Huber sp. nov.

44851572-139E-528E-BF51-0328BFC80DF3

https://zoobank.org/7D4B69A6-ED3D-4AA2-B9C4-AAAEADCDC383

Figs 4F , 5C, D, H , 6F, G , 8D–F , 9D, H, I , 10E, F , 11C, I , 12E–G , 64 , 65 , 66 , 67

##### Type material.

***Holotype*.** Morocco – **Fès-Meknès** • ♂; Kef el Ghar (=Rhar); 34.4788°N, 4.2766°W; 620 m a.s.l.; 22 Sep. 2018; B.A. Huber leg.; ZFMK Ar 24684.

##### Other material examined.

Morocco – **Fès-Meknès** • 14 ♂♂, 12 ♀♀ (1 ♂, 1 ♀ used for SEM); same collection data as for holotype; ZFMK Ar 24685 to 24686 • 2 ♂♂, 2 ♀♀, 1 juv., in pure ethanol; same collection data as for holotype; ZFMK Mor100 • 1 ♂, 2 ♀♀; same locality as for holotype, 2 Jun. 1978; P. Strinati leg.; MHNG • 1 ♂, 4 ♀♀, 6 juvs; same locality as for holotype, 2 Jun. 1978; B. Hauser leg.; MHNG.

##### Diagnosis.

Distinguished from similar congeners (*M.agadir*, *M.tegulifer*) by unique shape of uncus (Fig. 65D, E; with sickle-shaped process and series of pointed processes along edge), by unique shape of appendix (Fig. 65D, E; two small pointed processes proximally, larger process distally, and membranous distal area), and by large sclerotised triangular plate on epigynum with whitish median area (Fig. 67A); also by pair of distinctive membranous structures laterally in female internal genitalia (arrows in Fig. 66C); from *M.tegulifer* also by presence of two pairs of processes proximally on male chelicerae (Fig. 66A, B; absent in *M.tegulifer*), by lateral marks on carapace (Fig. 4F; absent in *M.tegulifer*), and by roundish rather than elongate pore plates (Fig. 66C).

**Figure 64.**
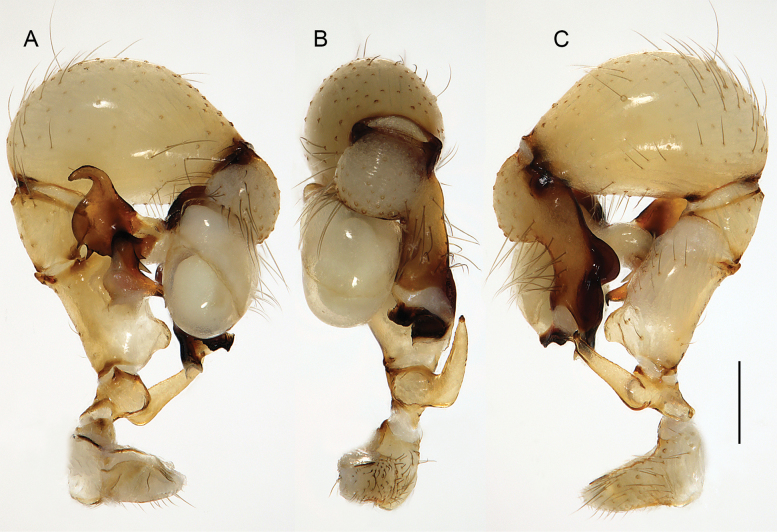
*Micropholcusghar* Huber, sp. nov.; male from Morocco, Fès-Meknès, Kef el Ghar (ZFMK Ar 24685). Left palp in prolateral (**A**), dorsal (**B**), and retrolateral (**C**) views. Scale bar: 0.3 mm.

**Figure 65.**
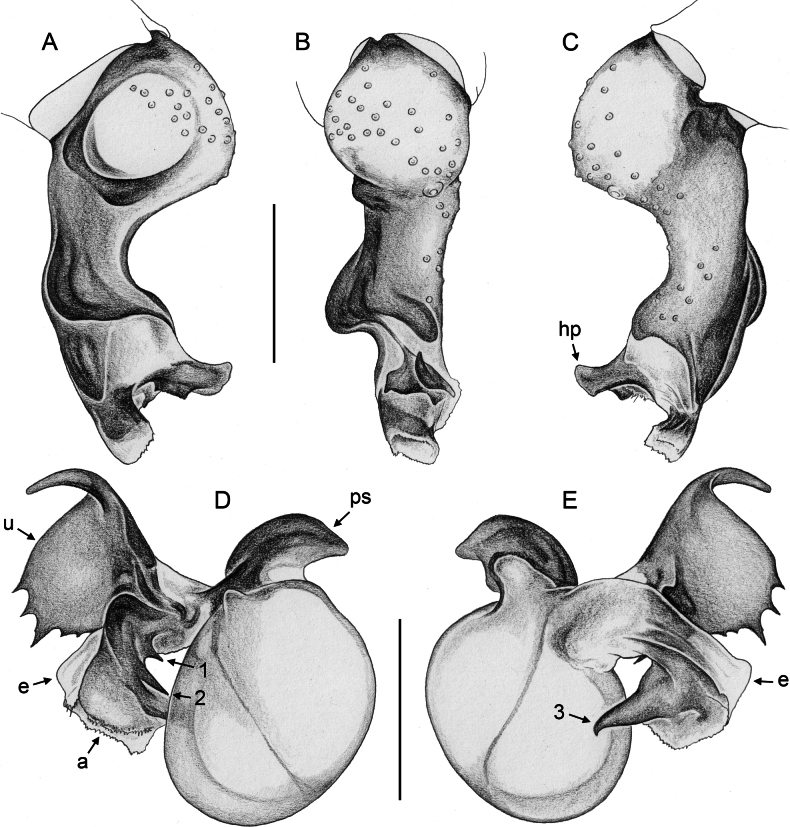
*Micropholcusghar* Huber, sp. nov.; male from Morocco, Fès-Meknès, Kef el Ghar (ZFMK Ar 24685) **A–C** left procursus in prolateral, dorsal, and retrolateral views **D, E** left genital bulb in prolateral and retrolateral views; numbers 1–3 denote pointed processes of appendix. Abbreviations: a, putative appendix; e, embolus; hp, dorsal hinged process; ps, proximal bulbal sclerite; u, putative uncus. Scale bars: 0.3 mm.

**Figure 66.**
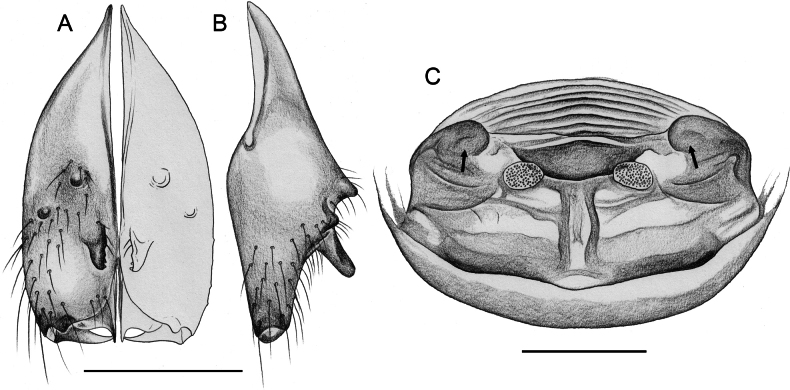
*Micropholcusghar* Huber, sp. nov.; from Morocco, Fès-Meknès, Kef el Ghar **A, B** male chelicerae, frontal and lateral views (ZFMK Ar 24685) **C** cleared female genitalia, dorsal view (ZFMK Ar 24686); arrows point at distinctive membranous structures. Scale bars: 0.3 mm.

**Figure 67.**
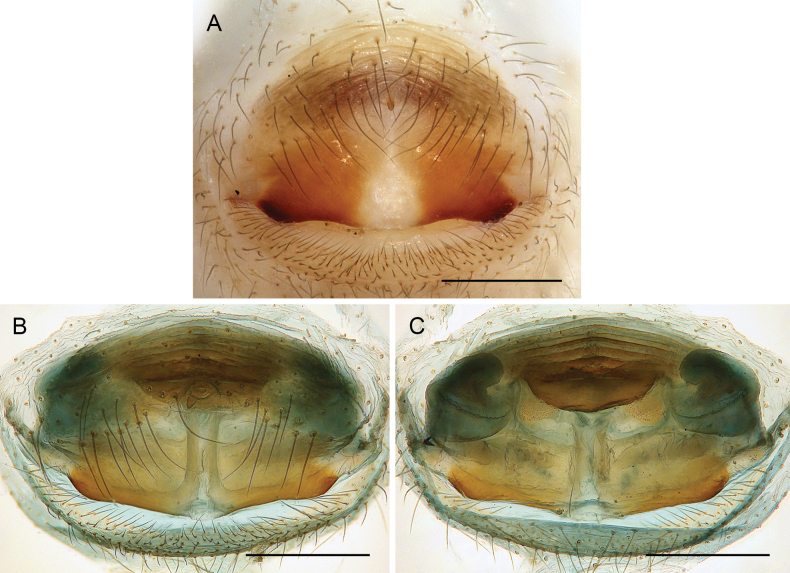
*Micropholcusghar* Huber, sp. nov.; female from Morocco, Fès-Meknès, Kef el Ghar (ZFMK Ar 24686) **A** epigynum, ventral view **B, C** cleared female genitalia, ventral and dorsal views. Scale bars: 0.3 mm.

##### Description.

**Male** (holotype). ***Measurements*.** Total body length 3.6, carapace width 1.2. Distance PME-PME 200 µm; diameter PME 85 µm; distance PME-ALE 25 µm; distance AME-AME 20 µm; diameter AME 45 µm. Leg 1: 37.6 (9.7 + 0.6 + 9.7 + 15.9 + 1.7), tibia 2: 6.8, tibia 3: 4.2, tibia 4: 5.7; tibia 1 L/d: 84; diameters of leg femora (at half length) ~ 0.13; of leg tibiae 0.11–0.12.

***Colour*** (in ethanol). Prosoma and legs mostly pale ochre-yellow, carapace with light brown marks, ocular area and clypeus without darker pattern, sternum with brown margins and three light brown marks posteriorly; legs with slightly darkened patellae, tibia-metatarsus joints not darkened; abdomen monochromous pale grey to whitish.

***Body*.** Habitus as in Fig. 4F. Ocular area raised (distinct in frontal view; Fig. 5C). Carapace without thoracic groove. Clypeus unmodified. Sternum wider than long (0.80/0.60), unmodified. Abdomen oval, approximately twice as long as wide. Gonopore with four epiandrous spigots (Fig. 10E). Spinnerets as in Fig. 9H, I.

***Chelicerae*.** As in Fig. 66A, B; with pair of long distal frontal apophyses, each with two cone-shaped hairs (Fig. 6F, G); and two pairs of smaller proximal processes.

***Palps*.** As in Fig. 64; coxa unmodified; trochanter with retrolateral-ventral apophysis provided with terminal modified hair (Fig. 9D); femur cylindrical with distinct ventral process proximally; femur-patella joints shifted toward prolateral side; tibia-tarsus joints slightly shifted toward retrolateral side. Procursus (Figs 8D, 65A–C) proximally with sclerotised prolateral ridge; at half length with strong prolateral-ventral sclerotised ridge or process; distally with dorsal hinged process; tip of procursus partly sclerotised and apparently also hinged against proximal part. Genital bulb (Figs 8E, F, 65D, E) with strong proximal sclerite; putative appendix with three pointed processes directed towards bulbous part, distally widened and membranous, with fringed membrane; putative uncus flat with series of pointed processes and one long curved process; and mostly semi-transparent embolus.

***Legs*.** Without spines, without curved hairs, without sexually dimorphic short vertical hairs (many hairs missing in holotype but confirmed in other males); retrolateral trichobothrium of tibia 1 at 5%; prolateral trichobothrium absent on tibia 1; tarsus 1 with > 20 pseudosegments, distally distinct.

***Variation*** (male). Tibia 1 in 18 males (incl. holotype): 6.2–10.2 (mean 8.1). While most elements of the bulbal processes (and procursus) appear to be very consistent, there is substantial variation in the row of pointed processes on the uncus. The number of larger processes ranges from two to four; the smaller processes may be absent or replaced by a single (sometimes larger) process; several males were asymmetric in this respect.

**Female.** In general very similar to male but abdomen often much wider. Tibia 1 in 14 females: 6.0–9.1 (mean 7.3). Epigynum (Figs 10F, 67A) anterior plate divided into two sections, anterior section weakly sclerotised, with curved ridges and hairs; posterior section smooth, medially whitish, laterally brown to black, i.e., heavily sclerotised; small knob-shaped process (Fig. 11C) between posterior and anterior parts; posterior epigynal plate short and very indistinct. Internal genitalia (Figs 66C, 67B, C) with pair of small oval pore plates, distinctive median sclerite, and pair of large membranous structures laterally.

##### Etymology.

The species name is derived from the type locality; noun in apposition.

##### Distribution.

Known from two localities in Morocco, both in Fès-Meknès Region (Fig. 13D). We could not examine the single male specimen mentioned in Lecigne et al. (2023: 71), originating from Tazekka National Park, Ghar Admam, 34.0278°N, 4.1509°W. However, photographs of the male palp kindly provided by S. Lecigne leave little doubt that this is the same species.

##### Natural history.

The spiders were very abundant within the first ~ 100 m of the cave; no specimens were seen outside the cave or in deeper sections. They built their fine and slightly domed webs close to the floor, often under small rock overhangs. They were hanging in the apex of the dome rather than sitting on the rock. At disturbance, they bounced slightly and walked towards the rock.

#### ﻿﻿ Micropholcus khenifra

Taxon classificationAnimaliaAraneaePholcidae

Huber, Lecigne & Lips sp. nov.

87FBD744-43E6-5566-AE7F-70C8835376F3

https://zoobank.org/A1FA9AD3-2DCE-4303-B128-9966534808DE

**Figure 68.**
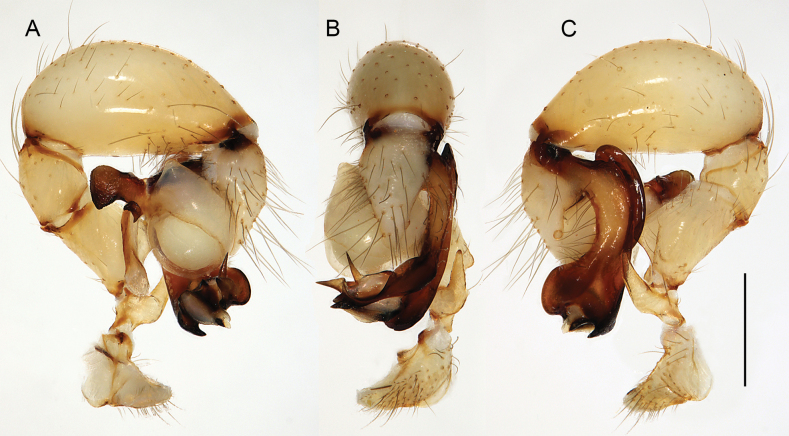
*Micropholcuskhenifra* Huber, Lecigne & Lips, sp. nov.; male from Morocco, Béni Mellal-Khénifra, Imi n’Ifri (ZFMK Ar 24688). Left palp in prolateral (**A**), dorsal (**B**), and retrolateral (**C**) views. Scale bar: 0.5 mm.

**Figure 69.**
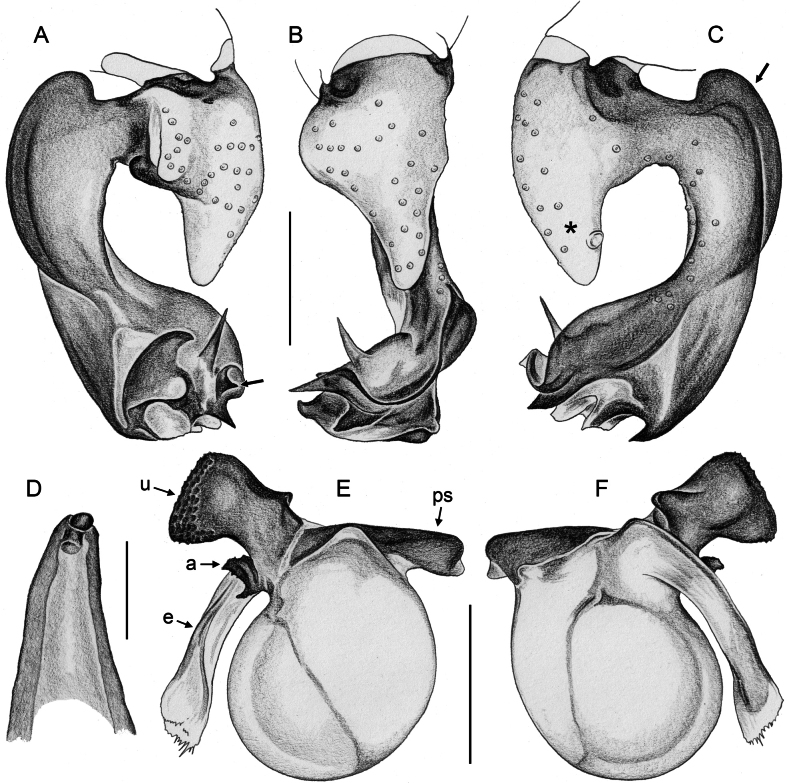
*Micropholcuskhenifra* Huber, Lecigne & Lips, sp. nov.; male from Morocco, Béni Mellal-Khénifra, Imi n’Ifri (ZFMK Ar 24688) **A–C** left procursus in prolateral, dorsal, and retrolateral views; bold arrow in A points at pointed process that is absent in males from near Sidi Ben Daoud and from W of El Ksiba; asterisk in C marks distinctive whitish process of tarsus; bold arrow in C points at flat ventral process of procursus **D** tip of palpal trochanter apophysis **E, F** left genital bulb in prolateral and retrolateral views. Abbreviations: a, putative appendix; e, embolus; ps, proximal bulbal sclerite; u, putative uncus. Scale bars: 0.3 mm (**A–C, E, F**); 0.05 mm (**D**).

**Figure 70.**
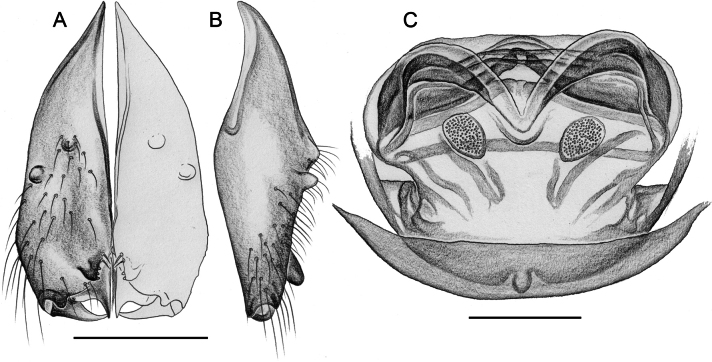
*Micropholcuskhenifra* Huber, Lecigne & Lips, sp. nov.; from Morocco, Béni Mellal-Khénifra, Imi n’Ifri **A, B** male chelicerae, frontal and lateral views (ZFMK Ar 24688) **C** cleared female genitalia, dorsal view (ZFMK Ar 24689). Scale bars: 0.3 mm.

**Figure 71.**
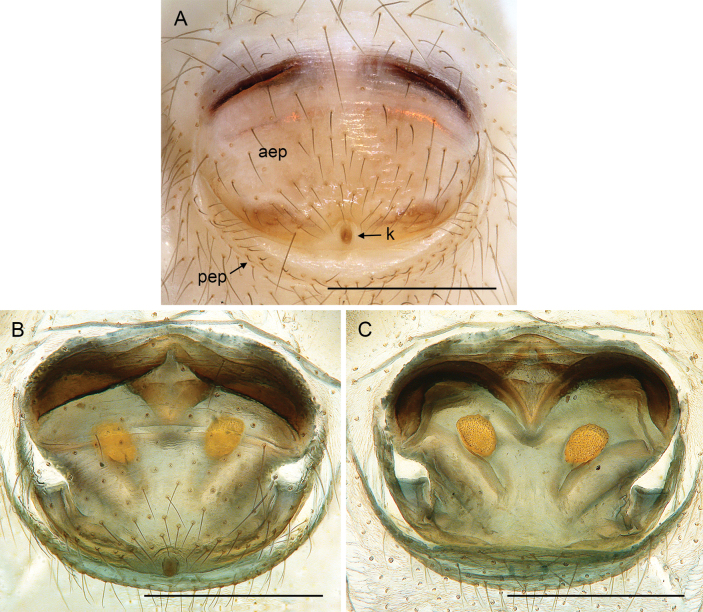
*Micropholcuskhenifra* Huber,Lecigne & Lips, sp. nov.; female from Morocco, Béni Mellal-Khénifra, near Sidi Ben Daoud (ZFMK Ar 24689) **A** epigynum, ventral view **B, C** cleared female genitalia, ventral and dorsal views. Abbreviations: aep, anterior epigynal plate; k, epigynal ‘knob’; pep, posterior epigynal plate. Scale bars: 0.5 mm.

Figs 4G , 68 , 69 , 70 , 71

##### Type material.

***Holotype*.** Morocco – **Béni Mellal-Khénifra** • ♂; Imi n’Ifri; 31.724°N, 6.971°W; 1050 m a.s.l.; 26 Sep. 2018; B.A. Huber leg.; ZFMK Ar 24687.

##### Other material.

Morocco – **Béni Mellal-Khénifra** • 5 ♂♂, 5 ♀♀; same collection data as for holotype; ZFMK Ar 24688 to 24689 • 2 ♂♂, 1 ♀, in pure ethanol; same collection data as for holotype; ZFMK Mor104 • 4 ♂♂, 4 ♀♀; near Sidi Ben Daoud; 32.5347°N, 6.1285°W; 700 m a.s.l.; 25 Sep. 2018; B.A. Huber leg.; ZFMK Ar 24690 to 24691 • 1 ♂, 1 ♀, 2 juvs, in pure ethanol; same collection data as for preceding; ZFMK Mor102 • 4 ♂♂, 3 ♀♀, 1 juv.; W of El Ksiba; 32.560°N, 6.053°W – 32.562°N, 6.050°W; 950 m a.s.l.; 25 Sep. 2018; B.A. Huber leg.; ZFMK Ar 24692 to 24693 • 1 ♀, 1 juv., in pure ethanol; same collection data as for preceding; ZFMK Mor103 • 1 ♀; Jbel Bou-Guergour, Ghar-el-Ghazi; 32.869°N, 5.689°W (?); 970 m a.s.l.; 26 May 2001; C. Ribera leg.; ZFMK Ar 24700.

##### Diagnosis.

Easily distinguished from known congeners by whitish dorsal process of male palpal tarsus (asterisk in Fig. 69C; absent in congeners), by large and prominent flat ventral process of procursus (arrowed in Fig. 69C; much smaller or absent in congeners), by complex tip of procursus with distinctive dorsal spine (Fig. 69A–C), by rounded uncus (Fig. 69E, F; similar only in *M.agadir*), by very small appendix (larger and more complex in Moroccan congeners), by long prominent embolus (Fig. 69E, F; in Moroccan congeners shorter and in prolateral view largely hidden behind uncus and appendix), by pair of dark internal structures visible at anterior margin of epigynum (Fig. 71A; absent in congeners), by distinctive m-shaped dorsal arc in female internal genitalia (Fig. 71C), and by very narrow (short) sclerotised band posteriorly on epigynum carrying epigynal ‘knob’ (Fig. 71A).

##### Description.

**Male** (holotype). ***Measurements*.** Total body length 3.9, carapace width 1.5. Distance PME-PME 205 µm; diameter PME 90 µm; distance PME-ALE 30 µm; distance AME-AME 20 µm; diameter AME 50 µm. Leg 1: 34.5 (8.7 + 0.6 + 9.0 + 14.4 + 1.8), tibia 2: 6.4, tibia 3: 4.0, tibia 4: 5.3; tibia 1 L/d: 75; diameters of leg femora (at half length) 0.14–0.15; of leg tibiae 0.12.

***Colour*** (in ethanol). Prosoma and legs mostly ochre-yellow, carapace with brown median mark, ocular area and clypeus without darker pattern, sternum with brown margins; legs with slightly darkened patellae, anterior femora ventrally only very slightly darkened, tibia-metatarsus joints not darkened; abdomen monochromous pale grey.

***Body*.** Habitus as in Fig. 4G. Ocular area raised (distinct in frontal view). Carapace without thoracic groove. Clypeus unmodified. Sternum wider than long (0.88/0.72), unmodified. Abdomen oval, approximately twice as long as wide.

***Chelicerae*.** As in Fig. 70A, B; with pair of strong distal frontal apophyses, each with two cone-shaped hairs; and two pairs of smaller proximal processes.

***Palps*.** As in Fig. 68; coxa unmodified; trochanter with ventral apophysis provided with terminal modified hair (Fig. 69D); femur cylindrical, proximally with small retrolateral process and larger prolateral-ventral process; femur-patella joints shifted toward prolateral side; tibia very large relative to femur; tibia-tarsus joints not shifted to one side; tarsus with cone-shaped light dorsal process carrying tarsal organ. Procursus (Fig. 69A–C) proximally with sclerotised prolateral ridge; proximal half with flat ventral process (arrowed in Fig. 69C), distally divided into dorsal and ventral parts and complex hinged structure between them (mostly on prolateral side), with pointed process originating from membranous connection between dorsal part and hinged process. Genital bulb (Fig. 69E, F) with strong proximal sclerite; putative appendix small; putative uncus flat with retrolateral process; and long, partly sclerotised embolus.

***Legs*.** Without spines, without curved hairs, without sexually dimorphic short vertical hairs; retrolateral trichobothrium of tibia 1 at 9%; prolateral trichobothrium absent on tibia 1; tarsus 1 with > 30 pseudosegments, distally distinct.

***Variation*** (male). Tibia 1 in 14 males (incl. holotype): 6.9–9.2 (mean 8.2). There was very slight variation in palpal structures among localities: in males from near Sidi Ben Daoud and from W of El Ksiba, the uncus was slightly rounder, the appendix slightly larger, and one small, pointed element of the dorsal part of the procursus (arrowed in Fig. 69A) was absent. The number of modified hairs on the frontal cheliceral apophyses was two or three, and was sometimes asymmetrical (as in Fig. 70A).

**Female.** In general very similar to male. Tibia 1 in 12 females: 7.1–8.4 (mean 7.7). Epigynum (Fig. 71A) anterior plate mostly light, anteriorly with pair of dark internal structures variably visible in untreated specimens, posteriorly with narrow darker transversal band and median ‘knob’; posterior epigynal plate short and very indistinct. Internal genitalia (Figs 70C, 71B, C) with pair of small oval pore plates and distinctive ventral and dorsal anterior arches and sclerites.

##### Etymology.

The species name is derived from Béni Mellal-Khénifra, the region in Morocco where all available specimens were collected; noun in apposition.

##### Distribution.

Known from several localities in Morocco, all in Béni Mellal-Khénifra Region (Fig. 13D).

##### Natural history.

At Imi N’ifri (Fig. 14G) and west of Ksiba, the spiders were found in small cavities of rocks, on the undersides of very large boulders, and in small caverns at ground level. Near Sidi Ben Daoud, the spiders were found in a small cave from which a brook emerged. The spiders sat flat on the rock and appeared very unwilling to leave the spot upon disturbance. At all localities, *M.khenifra* sp. nov. was found in close proximity with *Holocnemusreini*.

#### ﻿﻿ Micropholcus bukidnon

Taxon classificationAnimaliaAraneaePholcidae

Huber sp. nov.

536B720C-254D-5624-8E19-DCFB2F716252

https://zoobank.org/F99122C7-C6F3-40B3-81BB-1DED94C7718E

Figs 4H , 5E, F , 6H , 8G, H , 9E, J , 10G, H , 11J , 12H , 72 , 73 , 74 , 75

Micropholcus Phi114 – Eberle et al. 2018 (molecular data). Huber et al. 2018: fig. 11.
Micropholcus sp. n. Phi114 – Huber and Eberle 2021, Suppl. material 1.

##### Type material.

***Holotype*.** Philippines – **Mindanao** • ♂; Bukidnon Province, Central Mindanao University, Faculty Hill; 7.852°N, 125.048°E; 330 m a.s.l.; on rocks in degraded forest; 10 Feb. 2014; B.A. Huber leg.; ZFMK Ar 24694.

##### Other material.

Philippines – **Mindanao** • 7 ♂♂, 10 ♀♀, 1 juv. (1 ♂, 1 ♀ used for SEM); same collection data as for holotype; ZFMK Ar 24695 to 24696 • 2 ♂♂, 3 ♀♀, in pure ethanol; same collection data as for holotype; ZFMK Phi 273 • 3 ♂♂, 2 ♀♀; Barangay San Jose, Blue Water Cave; 7.705°N, 125.035°E; 230 m a.s.l.; at rocks at cave entrance; 16 Feb. 2014; B.A. Huber leg.; ZFMK Ar 24697 • 2 ♂♂, 2 ♀♀, 1 juv., in pure ethanol; same collection data as for preceding; ZFMK Phi 250 • 7 ♀♀; Barangay San Jose, Kabyaw Cave; ~ 7.704°N, 125.038°E; ~ 260 m a.s.l.; at rocks at cave entrance; 16 Feb. 2014; B.A. Huber leg.; ZFMK Ar 24698.

##### Diagnosis.

**Figure 72.**
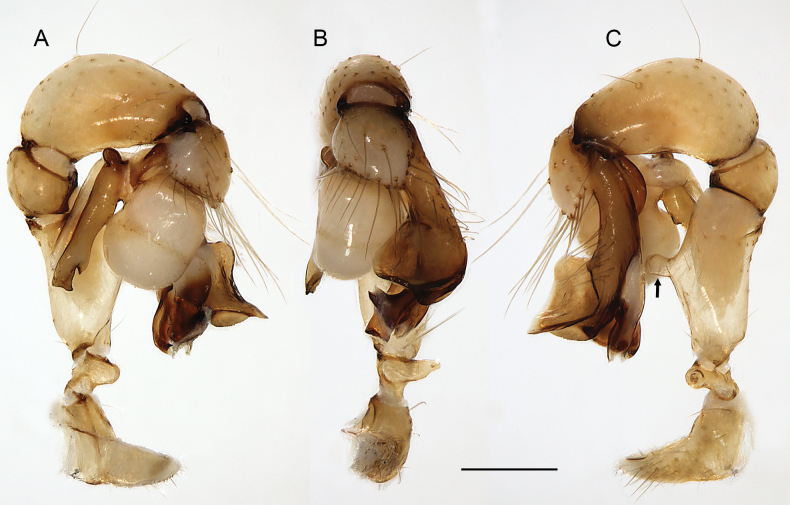
*Micropholcusbukidnon* Huber, sp. nov.; male from Philippines, Mindanao, Central Mindanao University (ZFMK Ar 24695); left palp in prolateral (**A**), dorsal (**B**), and retrolateral (**C**) views; arrow in C points at distinctive ventral process on femur. Scale bar: 0.3 mm.

**Figure 73.**
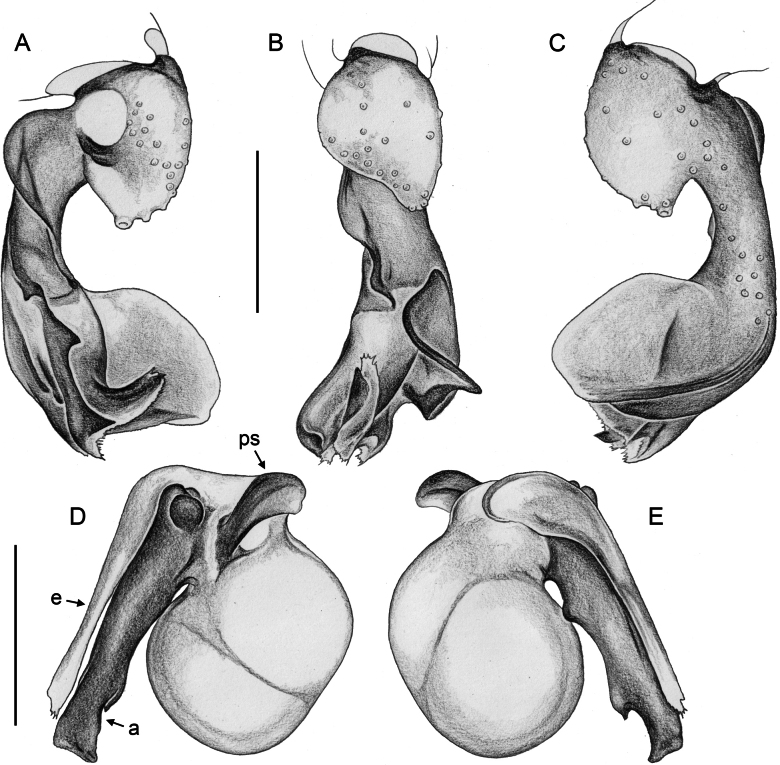
*Micropholcusbukidnon* Huber, sp. nov.; male from Philippines, Mindanao, Central Mindanao University (ZFMK Ar 24695) **A–C** left procursus in prolateral, dorsal, and retrolateral views **D, E** left genital bulb in prolateral and retrolateral views. Abbreviations: a, putative appendix; e, embolus; ps, proximal bulbal sclerite. Scale bars: 0.3 mm.

**Figure 74.**
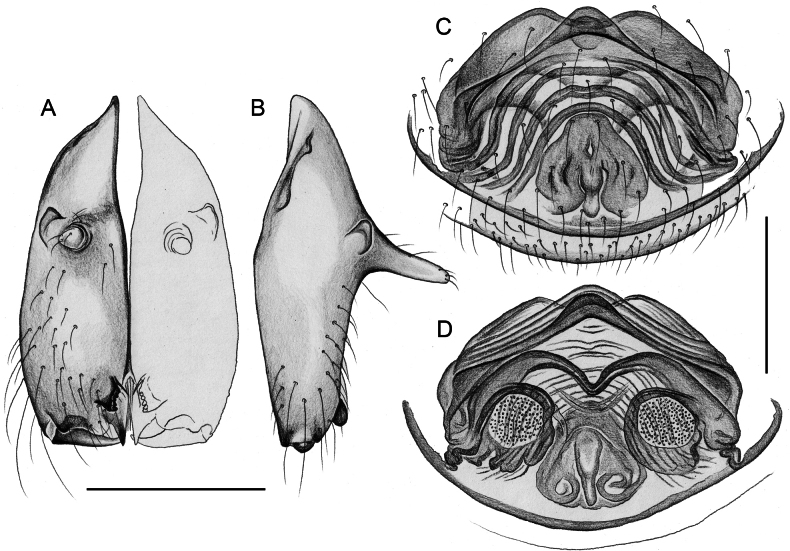
*Micropholcusbukidnon* Huber, sp. nov.; from Philippines, Mindanao, Central Mindanao University **A, B** male chelicerae, frontal and lateral views (ZFMK Ar 24695) **C, D** cleared female genitalia, ventral and dorsal views (ZFMK Ar 24696). Scale bars: 0.3 mm.

**Figure 75.**
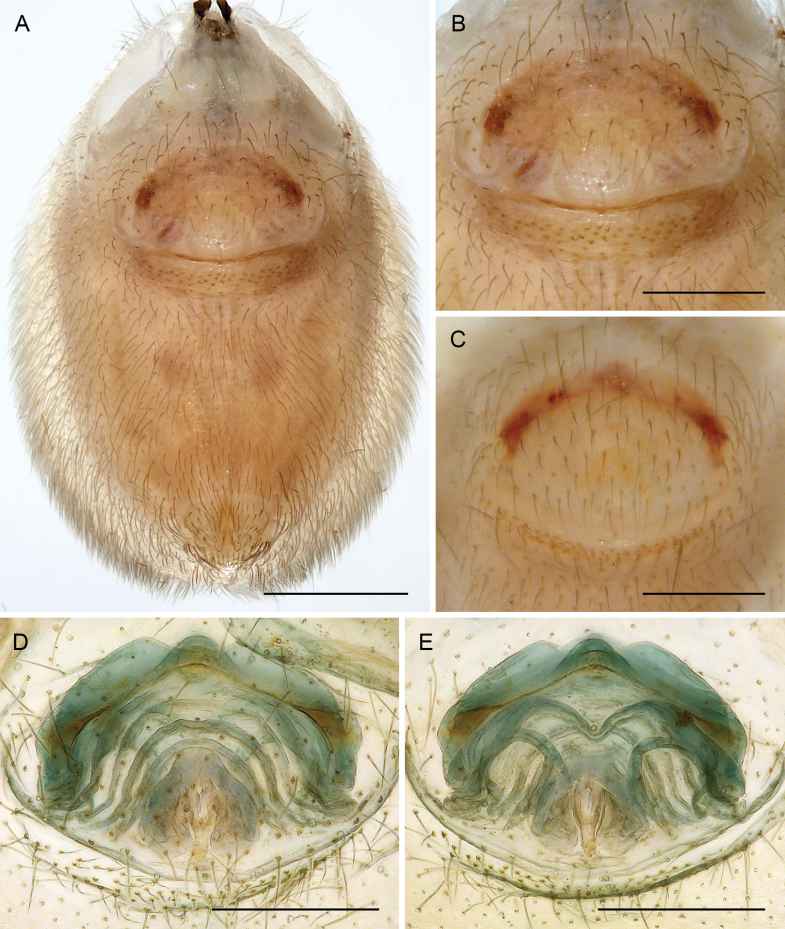
*Micropholcusbukidnon* Huber, sp. nov.; females from Philippines, Mindanao, Central Mindanao University. (ZFMK Ar 24696) **A** abdomen, ventral view **B, C** epigyna of two females, ventral views **D, E** cleared genitalia, ventral and dorsal views. Scale bars: 0.5 mm (**A**); 0.3 mm (**B–E**).

Easily distinguished from known congeners by unusually long proximal frontal apophyses on male chelicerae (Fig. 74B); by long ventral process of palpal femur (Fig. 72C); by long rod-shaped putative appendix (Fig. 73D, E), and by distinctive shapes of processes on procursus (Fig. 73A–C; in particular large flat dorsal process). Female genitalia very simple externally (Fig. 75A), distinguished from congeners by absence of external knob (Fig. 10H) and by distinctive internal structures (round pore plates; m-shaped dorsal arch; concentric ventral arches; distinctive median membranous structures posteriorly).

##### Description.

**Male** (holotype). ***Measurements*.** Total body length 2.8, carapace width 1.1. Leg 1: 28.1 (6.7 + 0.5 + 7.1 + 12.4 + 1.4), tibia 2: 4.5, tibia 3: 2.9, tibia 4: 3.8; tibia 1 L/d: 77. Distance PME-PME 190 µm, diameter PME 100 µm, distance PME-ALE ~ 30 µm; distance AME-AME 30 µm, diameter AME 15 µm.

***Colour*** (in ethanol). Carapace pale ochre with dark median band widening posteriorly, ocular area and clypeus only slightly darkened; sternum pale ochre with narrow dark margins; legs ochre to light brown, with dark brown patellae and tibia-metatarsus joints; abdomen monochromous pale grey.

***Body*.** Habitus as in Fig. 4H; ocular area slightly raised (Fig. 5E); carapace without median furrow; clypeus unmodified; sternum wider than long (0.65/0.55), unmodified. Gonopore of scanned male with five epiandrous spigots (Fig. 10G). Anterior lateral spinnerets with one strongly widened, one pointed, and six cylindrically shaped spigots (Fig. 9J).

***Chelicerae*.** As in Fig. 74A, B; proximally with pair of long frontal apophyses and pair of short lateral processes directed towards proximal, distally with pair of dark apophyses near laminae, each provided with five modified (globular) hairs (Fig. 6H).

***Palps*.** As in Fig. 72; coxa unmodified; trochanter with retrolateral apophysis, tip of apophysis without modified hair (Fig. 9E); femur with low retrolateral hump proximally and with distinctive ventral process; procursus (Figs 8G, 73A–C) very complex distally, with apparently hinged ventral structures and large dorsal flap; genital bulb (Figs 8H, 73D, E) with strong proximal sclerite, with long weakly sclerotised embolus and heavily sclerotised cylindrical putative appendix with proximal round protrusion and short subdistal branch.

***Legs*.** Without spines and curved hairs; without sexually dimorphic short vertical hairs; retrolateral trichobothrium on tibia 1 at 10%; prolateral trichobothrium absent on tibia 1, present on other tibiae; tarsus 1 with ~ 15 pseudosegments, only distally distinct. Tarsus 4 comb-hairs as in Fig. 12H.

***Variation*** (male). Tibia 1 in nine other males: 5.4–7.3 (mean 6.5); specimens from Barangay San Jose have consistently shorter legs than specimens from Faculty Hill (5.4–5.7 versus 6.6–7.3).

**Female.** In general similar to male; eye triads at almost same distance (PME-PME: 170 µm; Fig. 5F). Dark band on carapace in some females posteriorly not widened. Tibia 1 in 19 females: 4.3–6.0 (mean: 5.2). As in males, specimens from Barangay San Jose have consistently shorter legs than specimens from Faculty Hill (4.3–5.2 versus 5.4–6.0). Epigynum very simple (Figs 10H, 75A–C), weakly sclerotised, without external ‘knob’; internal genitalia (Figs 74C, D, 75D, E) with round pore plates, m-shaped dorsal arch, concentric ventral arches, and distinctive median membranous structures posteriorly.

##### Etymology.

The species name is derived from the type locality; noun in apposition.

##### Distribution.

Known from three localities (two of them very close to each other) in central Mindanao, Philippines (Fig. 13B).

##### Natural history.

The spiders were found on rocks, either on the undersides of large rocks with sufficient space to the ground, or in small depressions of near-vertical rock-surfaces (Fig. 14H). Two egg sacs contained 11 and 25 eggs, respectively, with an egg diameter of 0.54–0.57 mm (Huber and Eberle 2021).

## ﻿﻿Discussion

### ﻿Species limits

Our data on Saudi Arabian *Micropholcus* are difficult to interpret. From a morphological perspective, there are consistent differences among specimens assigned herein to different nominal species. These differences are at approximately the same level of distinctness as between congeners in many other Pholcidae genera. In addition, the respective traits are very homogeneous within putative species. From a molecular perspective, however, our data suggest different species limits, in particular among the southern group of Saudi Arabian species: *M.alfara* sp. nov., *M.dhahran* sp. nov., and *M.harajah* sp. nov. Among these, the genetic distances of 3.4–6.7% are clearly below the problematic range of overlap between intra- and interspecific distances reported for Pholcidae (usually ~ 8–12%; Astrin et al. 2006; Huber et al. 2023b, 2024a, 2024b). The ASAP analysis suggests that this group contains only one or two species, rather than three. We here give more weight to the morphological evidence, but acknowledge that this is in need of further research. At this point, almost every locality at higher elevations with suitable habitats visited in Saudi Arabia has its own ‘species’ of *Micropholcus*, and we predict that further collecting in this area and in neighbouring Yemen will dramatically increase the number of possible species.

### ﻿The geographic origin of *Micropholcusfauroti*

Synanthropic species, i.e., species ecologically associated with humans, have often attained their wide distributions long before they were studied in any detail (Baumann 2023), and their geographic origins and spreading histories are thus usually undocumented (e.g., Molero-Baltanás et al. 2024). Their ancestral areas can only be reconstructed by phylogenetic and geographic analyses of the most closely related taxa (e.g., García-Vázquez and Ribera 2016). In Pholcidae, a dozen species have attained worldwide or pan-tropical distributions, and a few more have probably extended their ranges with the aid of humans. Their spreading histories are mostly undocumented (but see Fürst and Blandenier 1993) and can only be reconstructed indirectly.

*Micropholcusfauroti* was first described from Djibouti (Simon 1887) but a few years later recorded from Myanmar (Thorell 1895, as *Pholcusinfirmus*), and in 1929 from the New World, from Brazil (Mello-Leitão 1929, as *Leptopholcusoccidentalis*) and from Puerto Rico (Petrunkevitch 1929, as *Pholcusunicolor*). It has long been thought to have an Old World origin, especially since the description of *M.jacominae* from Yemen (Deeleman-Reinhold and van Harten 2001), and the newly described species from Saudi Arabia support this idea. The very distinctive dorsal hinged process of *M.fauroti* resembles that of the Saudi Arabian species more than any other congener. However, *M.fauroti* lacks the distinctive membranous flap prolaterally on the procursus seen in Saudi Arabian species (cf. Figs 7A, D, 8B), and the bulbal processes appear easier to homologise with those of *M.jacominae* than with those of Saudi Arabian species. Our molecular analysis does not include *M.jacominae*, and it does not clearly associate *M.fauroti* with any sequenced congener (the sister group relationship with the Moroccan species in Fig. 1 is poorly supported and very probably an artifact). We thus hypothesise that further collecting in Yemen has good chances to find even closer relatives of *M.fauroti*.

### ﻿Acroceridae larvae in Pholcidae book lungs

Flies (Diptera) are known to attack spiders in a variety of ways, as predators, egg parasitoids and predators, kleptoparasites, and endoparasitoids (Gillung and Borkent 2017). The latter category is, among the Diptera, the domain of a single family, the Acroceridae Leach, 1815. Acroceridae larvae are thought to develop exclusively in true spiders (Schlinger 1987), with an apparent preference for cursorial and fossorial species. True web-building spiders are rarely attacked, which is probably due to the fact that the fly eggs are deposited on the substrate and the emerging larvae must actively search for their hosts (Schlinger 1987). Pholcidae are web-building spiders, and this may partly explain why Acroceridae have never been reported to parasitise representatives of this spider family. However, in some pholcids, the apex of the domed sheet component of the web is closely attached to the substrate, and this is the section of the web where the spider spends most of the day. This is also the case in most *Micropholcus*, where the spiders often appear to be sitting directly flat on the rock surface, due to the very delicate and sparse web (Fig. 15).

**Figure 76.**
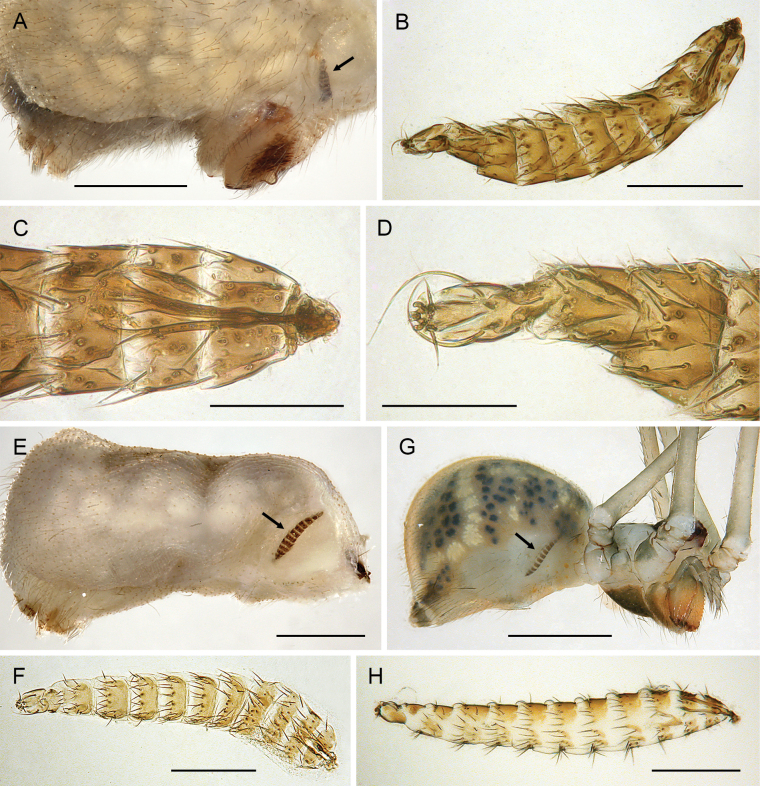
Acroceridae larvae in book lungs of Pholcidae; arrows point at larvae as seen in untreated abdomens **A** female abdomen of *Micropholcusbashayer* Huber, sp. nov., from Saudi Arabia, ‘Asir, NW of Al Bashayer (ZFMK Ar 24666) **B–D**Acroceridae larva extracted from A **E** male abdomen of *Micropholcusdarbat* Huber, sp. nov., from Oman, Dhofar, Wadi Darbat (ZFMK Ar 24672) **F**Acroceridae larva extracted from E **G***Mesabolivareberhardi* Huber, 2000, from Colombia, Magdalena, at Cascada Valencia (ZFMK Col138) **H**Acroceridae larva extracted from G. Scale bars: 0.5 mm (**A, E, G**); 0.1 mm (**B, F, H**); 0.05 mm (**C, D**).

Acroceridae larvae are here reported from two species of *Micropholcus* from Saudi Arabia (Fig. 76A–F), and, for the sake of completeness, from a juvenile of *Mesabolivareberhardi* from Colombia (Fig. 76G, H). The first author has seen a probable fourth case, but the larva was not extracted and the material has been returned to the California Academy of Sciences: a female of *Paramicromerysrothorum* Huber, 2003 from Madagascar (Antsiranana, Montagne d’Ambre). This suggests that Pholcidae may in fact be fairly common hosts for Acroceridae worldwide. Future collectors should try to keep spiders alive in which larvae can be seen through the book lung covers (Fig. 76A, E, G). Rearing of spiders is the most prolific source of information of spider-fly relationships (Kehlmaier et al. 2024). Rearing infected pholcids is particularly worthwhile because we cannot exclude that the pholcids are attacked accidentally, as is suspected to be the case at least in Acari (Schlinger 1987; Gillung and Borkent 2017).

## ﻿﻿Conclusions

*Micropholcus* in the Old World has a wide geographic range but seems to be largely restricted to semiarid regions, where the spiders lead reclusive lives in caves, in cave-like spaces under rocks, and in rock depressions. The genus is species-rich both on the Arabian Peninsula and in Morocco, suggesting that it should also be present in suitable habitats in the large but poorly sampled area in-between. The Philippine *M.bukidnon* sp. nov. extends the distribution of the genus far to the east, but this species is morphologically exceptional and its assignment to the genus rests on molecular evidence only.

*Micropholcus* appears to be exceptionally diverse in southwestern Saudi Arabia, which is generally considered as one of the richest biodiversity areas on the Arabian Peninsula (Abuzinada et al. 2005; Al-Namazi et al. 2021). We predict that a similar high species-richness will also be found in neighbouring Yemen. Saudi Arabian species seem to be restricted to high altitudes (above 1200 m a.s.l.), and most species are known from a single locality. Species from neighbouring localities are often morphologically distinct but genetically (CO1 distance) very close.

Acroceridae flies mainly attack cursorial and fossorial spider species (Gillung and Borkent 2017), probably due to the larval strategy to find a spider host (Schlinger 1987). Here we document the first cases of Acroceridae larvae developing in the book lungs of web-building Pholcidae. We suggest that *Micropholcus* spiders are accessible to Acroceridae larvae because they spend the day in the apex of the domed web that is closely attached to the rock surface. Rearing of infected spiders will be necessary to show if the larvae can actually develop in the spiders, or if pholcids are attacked accidentally.

## Supplementary Material

XML Treatment for Micropholcus

XML Treatment for Micropholcus fauroti

XML Treatment for Micropholcus jacominae

XML Treatment for Micropholcus dhahran

XML Treatment for Micropholcus harajah

XML Treatment for Micropholcus alfara

XML Treatment for Micropholcus abha

XML Treatment for Micropholcus tanomah

XML Treatment for Micropholcus bashayer

XML Treatment for Micropholcus maysaan

XML Treatment for Micropholcus darbat

XML Treatment for Micropholcus shaat

XML Treatment for Micropholcus agadir

XML Treatment for Micropholcus ghar

XML Treatment for Micropholcus khenifra

XML Treatment for Micropholcus bukidnon

## ﻿Additional information

### Author ORCIDs

Bernhard A. Huber https://orcid.org/0000-0002-7566-5424

Guanliang Meng https://orcid.org/0000-0002-6488-1527

### Data availability

All of the data that support the findings of this study are available in the main text or Supplementary Information.

## Supplementary materials

Supplementary material 1 CO1 K2P distances of the sequenced specimens Data type xlsx https://binary.pensoft.net/file/1141487This dataset is made available under the Open Database License (http://opendatacommons.org/licenses/odbl/1.0/). The Open Database License (ODbL) is a license agreement intended to allow users to freely share, modify, and use this Dataset while maintaining this same freedom for others, provided that the original source and author(s) are credited.Bernhard A. Huber, Guanliang Meng
